# PRC2 and EHMT1 regulate H3K27me2 and H3K27me3 establishment across the zygote genome

**DOI:** 10.1038/s41467-020-20242-9

**Published:** 2020-12-11

**Authors:** Tie-Gang Meng, Qian Zhou, Xue-Shan Ma, Xiao-Yu Liu, Qing-Ren Meng, Xian-Ju Huang, Hong-Lin Liu, Wen-Long Lei, Zheng-Hui Zhao, Ying-Chun Ouyang, Yi Hou, Heide Schatten, Xiang-Hong Ou, Zhen-Bo Wang, Shao-Rong Gao, Qing-Yuan Sun

**Affiliations:** 1 State Key Laboratory of Stem Cell and Reproductive Biology, Institute of Zoology, Chinese Academy of Sciences, Beijing, 100101 China; 2 Fertility Preservation Lab, Reproductive Medicine Center, Guangdong Second Provincial General Hospital, Guangzhou, 510317 China; 3 University of Chinese Academy of Sciences, Beijing, 100101 China; 4 Center for Reproductive Medicine, Henan Key Laboratory of Reproduction and Genetics, The First Affiliated Hospital of Zhengzhou University, Zhengzhou, Henan, 450052 China; 5 Clinical and Translational Research Center of Shanghai First Maternity and Infant Hospital, Shanghai Key Laboratory of Signaling and Disease Research, School of Life Sciences and Technology, Tongji University, Shanghai, 200092 China; 6 CAS Key Laboratory of Genome Sciences and Information, Beijing Institute of Genomics, Chinese Academy of Sciences, 100101 Beijing, China; 7 Department of Animal Genetics, Breeding and Reproduction, College of Animal Science and Technology, Nanjing Agricultural University, Nanjing, 210095 China; 8 Department of Veterinary Pathobiology, University of Missouri, Columbia, MO 65211 USA

**Keywords:** Oogenesis, Epigenetics, Histone post-translational modifications

## Abstract

The formation of zygote is the beginning of mammalian life, and dynamic epigenetic modifications are essential for mammalian normal development. H3K27 di-methylation (H3K27me2) and H3K27 tri-methylation (H3K27me3) are marks of facultative heterochromatin which maintains transcriptional repression established during early development in many eukaryotes. However, the mechanism underlying establishment and regulation of epigenetic asymmetry in the zygote remains obscure. Here we show that maternal EZH2 is required for the establishment of H3K27me3 in mouse zygotes. However, combined immunostaining with ULI-NChIP-seq (ultra-low-input micrococcal nuclease-based native ChIP-seq) shows that EZH1 could partially safeguard the role of EZH2 in the formation of H3K27me2. Meanwhile, we identify that EHMT1 is involved in the establishment of H3K27me2, and that H3K27me2 might be an essential prerequisite for the following de novo H3K27me3 modification on the male pronucleus. In this work, we clarify the establishment and regulatory mechanisms of H3K27me2 and H3K27me3 in mouse zygotes.

## Abstract (web summary)

Dynamic arrangement of epigenetic modifications such as repressive H3K27 methylation is essential for zygote development. Here the authors show that establishment of genome-wide H3K27me3 in zygotes requires EZH2, that EZH1 partially compensates for EZH2 loss, and that EHMT1 is involved in H3K27me2 establishment.

## Introduction

Life begins with the union of a sperm and an oocyte. Understanding how epigenetic modifications are regulated has been a major challenge in developmental biology, and, so far, the regulatory mechanisms of histone modification changes in zygotes is far from clear. H3K27me3 is a hallmark of facultative heterochromatin in numerous organisms, which is catalyzed by PRC2 (polycomb repressive complex 2) comprised of EED (embryonic ectoderm development), EZH1/2 (enhancer of zeste 1/2), and SUZ12 (suppressor of zeste 12). It is interesting that the phenotypes of *Ezh2* and *Eed* maternal knockout are inconsistent. The former displays a severe growth retardation^1^, while the latter shows significant reduced litter size and a significant overgrowth^2,3^. Unlike EZH2, which is indispensable, EZH1 is not necessary for the development of mouse^4^. *Ezh1* depletion neither reduced global H3K27me2/3 levels nor impacted viability and fertility in mice^4,5^. However, it is also reported that PRC2-EZH1 directly and robustly represses transcription from chromatinized templates and compacts chromatin in the absence of the methyltransferase cofactor SAM^5^. Interestingly, PRC2 displays post-translational modifications of automethylation in its own subunits to modulate its histone methyltransferase activity^6^, which then is demonstrated through its EZH1/2-mediated automethylation activity^7^.

H3K27me3 is a mark of facultative heterochromatin and gene silencing. Maternal H3K27me3 can function as an imprinting mark. Specifically, Inoue et al.^8^ proved that H3K27me3 modification on differential histones in the parent genome was a new regulatory mechanism for gene imprinting independent of DNA methylation. Erhardt et al.^1^ reported that X-inactivation (XCI) was stably propagated thereafter in zygotes lacking maternal EZH2. In a follow-up study, Inoue et al.^3^ recently demonstrated that loss of maternal H3K27me3 induced maternal *Xist* expression and maternal XCI in preimplantation embryos by injecting mRNA coding an H3K27me3-specific demethylase, *Kdm6b*. Furthermore, Inoue also showed that *Xist* was derepressed from the maternal X chromosome (Xm), resulting in biallelic XCI in females and XCI in males at the morula embryos in the absence of maternal *Eed*. Their results also indicated that XmCI was aberrant in Eed matKO pre-implantation embryos. Consistent with this, loss of EED from growing oocytes resulted in a significant overgrowth phenotype, which is the opposite of the ablation of EZH2 from growing oocytes.

Optimized ChIP-seq technologies such as STAR ChIP-seq^9^ and ULI-NChIP^10,11^ by reducing the reaction volume to prevent chromatin loss has contributed to great achievements. Using STAR ChIP-seq, Zheng et al.^12^ found that the sperm H3K27me3 was erased across the genome after fertilization. Surprisingly, H3K27me3 in oocytes is selectively retained in zygotes. Among them, H3K27me3 in the promoter region of development-related genes was rapidly deliberately erased after fertilization, while H3K27me3 in the non-promoter region was retained^12^.

However, the distribution pattern of H3K27me2 in the male and female pronuclear genomes after fertilization in mice and whether there is also a reprogramming process similar to H3K27me3 remain still unknown. The interaction between histone H3K27me2 and H3K27me3 in mouse zygotes, especially in the male pronucleus, has not been confirmed. It was shown that EHMT1 and EHMT2 could catalyze H3K27me1 and H3K27me2 directly in vitro^13^. Besides, it was also demonstrated that EZH2 and EHMT2/EHMT1 shared an important number of common genomic targets, encoding developmental and neuronal regulators^14^. Furthermore, EHMT2/EHMT1 complex could recruit PRC2 to the corresponding site for H3K27 methylation modification in mouse ES cells^14^.

In this work, through a series of in vitro experiments, conditional knockout mouse models, and new microsequencing technology ULI-NChIP, we show that EZH1 could partially safeguard the role of EZH2 in the formation of H3K27me2 but not for H3K27me3 in mouse zygotes, and we identify EHMT1 cooperates with PRC2 to regulate the establishment of H3K27me2. In addition, H3K27me2, which is broadly distributed throughout the genome, might be an essential prerequisite for the subsequent de novo H3K27me3 modification in the male pronucleus in a cell-cycle-dependent manner.

## Results

### Maternal EZH2 is indispensable for H3K27me3 rather than H3K27me2 in mouse zygotes

**Fig. 1:**
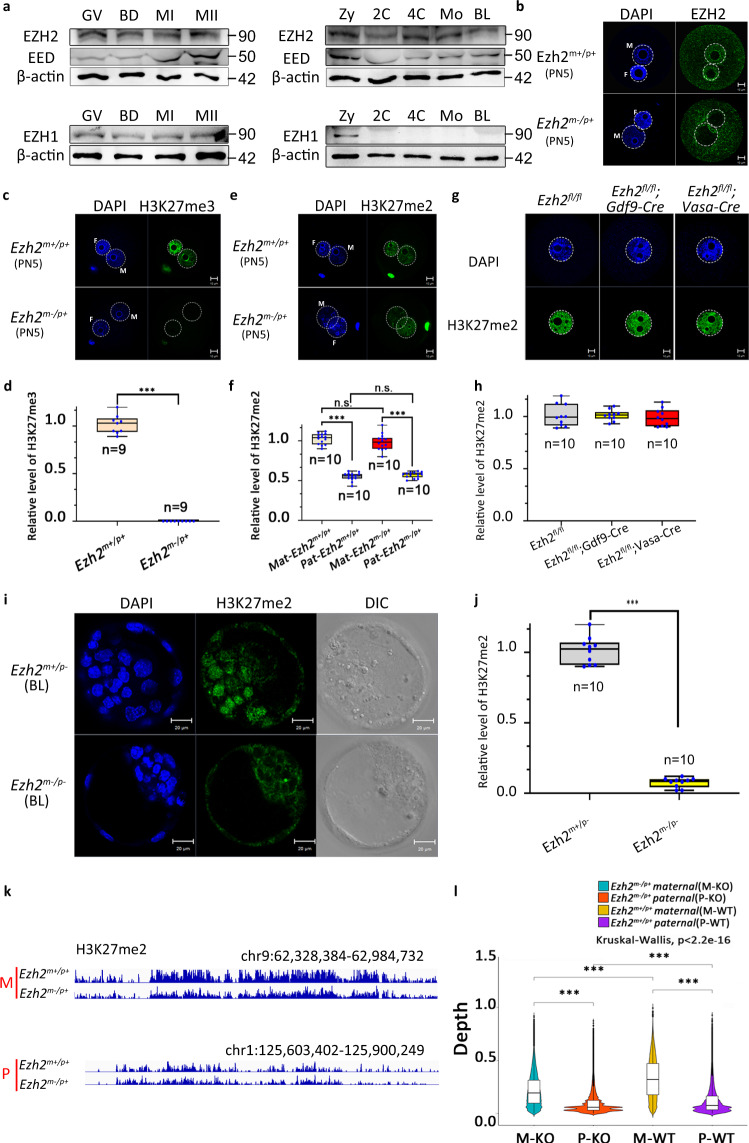
Maternal deletion of EZH2 prevents the establishment of H3K27me3, while its effect on the establishment of H3K27me2 is limited. **a** The expression pattern of several PRC2 components during oocyte maturation and early embryonic development. Left: Expression pattern of EZH2, EED, and EZH1 during oocyte maturation. Right: Expression pattern of EZH2, EED, and EZH1 during early embryonic development.) The signal of EZH2 in control and *Ezh2*^*m−/p+*^ zygotes at 13 h of IVF. M indicates male pronucleus and F indicates female pronucleus in the zygote. Scale bar, 10 μm. **c** The H3K27me3 state of zygotes at 13 h of IVF after the maternal loss of EZH2. M indicates male pronucleus and F indicates female pronucleus in the zygote. Scale bar, 10 μm. **d** Relative fluorescence intensity of H3K27me3 at PN5 stage *Ezh2*^*m+/p+*^ and *Ezh2*^*m−/p+*^ zygote pronucleus. Error bars, S.E.M. ***P < 8.81832E−16 by two-tailed Student’s *t* tests. **e** The H3K27me2 state of zygotes at 13 h of IVF after the maternal loss of EZH2. M indicates male pronucleus and F indicates female pronucleus in the zygote. Scale bar, 10 μm. **f** Relative fluorescence intensity of H3K27me2 at PN5 stage *Ezh2*^*m+/p+*^ and *Ezh2*^*m−/p+*^ zygote pronucleus. Error bars, S.E.M. ***P < 9.32605E-13 and *P* < 2.27984E−09, n.s. represents the nonsignificant difference. *P* > 0.3452 and *P* > 0.4783 by two-tailed Student’s *t* tests. Source data are provided as a Source data profile. **g** The H3K27me2 state of GV oocytes derived from *Ezh2*^*fl/fl*^*; Gdf9-cre* and *Ezh2*^*fl/fl*^*; Vasa-cre* female mice. Scale bar, 10 μm. **h** Relative fluorescence intensity of H3K27me2 in GV oocytes derived from *Ezh2*^*fl/fl*^*; Gdf9-cre* and *Ezh2*^*fl/fl*^*; Vasa-cre* female mice. Error bars, S.E.M. *P* > 0.9579 and *P* > 0.6631 by two-tailed Student’s *t* tests. n.s. represents a nonsignificant difference. Source data are provided as a Source data profile. **i** The H3K27me2 signal of *Ezh2*^*m+/p+*^ and *Ezh2*^*m−/p−*^ blastocysts. Embryos lacking EZH2 (*Ezh2*^*m−/p−*^) exhibited loss of H3K27me2 in ICM nuclei. Scale bar, 20 μm. **j** Relative fluorescence intensity of H3K27me2 in *Ezh2*^*m+/p+*^ and *Ezh2*^*m−/p−*^ blastocysts. Error bars, S.E.M. ***P < 1.61636E−16 by two-tailed Student’s *t* tests. Source data are provided as a Source data profile. **k** Global view of H3K27me2 distribution on the maternal and paternal genomes in Control and *Ezh2*^*m−/p+*^ zygotes. **l** Violin plot shows the depth of coverage of H3K27me2 signals on the maternal and paternal genomes in Control and *Ezh2*^*m−/p+*^ zygotes, respectively. For multiple comparisons, A Benjamini–Hochberg false-discovery rate-corrected *P* value (q value) was estimated, FDR < 0.05 was considered statistically significant. Kruskal–Wallis test, a nonparametric (distribution-free) test. Significance was determined by a pairwise comparison using two-sided Mann–Whitely *U* tests. A Benjamini–Hochberg false-discovery rate-corrected *P* value (*q* value) was estimated, FDR < 0.05 was considered statistically significant. *P*-values are: <2.2e−16, <2.2e−16, <2.2e−16, <2.2e−16 respectively. The median line of box plot represents the median, and the top and bottom of the box represent the upper and lower quartile, respectively.

First, we detected the expression pattern of PRC2 components during mouse oocyte maturation and early embryonic development (Fig. 1a, Supplementary Fig. 1a, b). Similar to EHMT1 level as revealed in our previous study^15^, EZH2 and EED were constantly expressed during oocyte maturation and early embryo development (Fig. 1a). Immunofluorescent staining of EZH2 also showed a constant nuclear localization (Supplementary Fig. 1b). However, EZH1 was not detectable in early embryos except for the zygotic stage. This difference in expression patterns suggests the possible stage-specific function of PRC2 components. Next, we investigated the functional significance of maternal EZH2 in vivo. We adopted a conditional deficiency approach in which the exon 4 allele of *Ezh2* was flanked by loxP sites^16^. We crossed conditional *Ezh2* mice with a *Gdf9-Cre* transgenic line to generate embryos that were deficient for maternal EZH2 and lacked detectable EZH2 protein in zygotes (*Ezh2*^*m−/p+*^) (Fig. 1b). Interestingly, although zygotes lacking maternal EZH2 showed loss of H3K27me3 in both pronuclei (Fig. 1c, d), the H3K27me2 level was still normal in the maternal pronucleus and de novo paternal pronucleus (Fig. 1e, f, Supplementary Fig. 1c–f). This surprising result that EZH2 was not responsible for H3K27me2 in zygotes led us to question whether H3K27me2 at other developmental stages was dependent on the catalytic activity of EZH2, such as stages prior to primordial follicle formation and blastocyst stage. Given that H3K27me2 exists in oocytes at E14.5 (Supplementary Fig. 2a), we crossed *Ezh2*^*fl/fl*^ mice with *Vasa-Cre* transgenic line for ablation of EZH2 at E13.5 to identify whether EZH2 was required for modification of H3K27me2 on maternal chromatin before primordial follicle formation. Surprisingly, the immunofluorescent staining and confocal analysis showed that there is no obvious change in the H3K27me2 relative fluorescence intensity compared to the control group in the GV oocytes (Fig. 1g, h). These results suggested that other factors might be involved in H3K27me2. However, in *Ezh2* null embryos, the situation was different. We crossed *Ezh2*^*fl/fl*^*;Gdf9-Cre* female mice with *Ezh2*^*fl/fl*^*;Vasa-Cre* male mice to generate *Ezh2*^*m−/p−*^ embryos, and found that these embryos began to show significant decrease of H3K27me2 from the morula stage (Fig. 1I, j, Supplementary Fig. 2b), which indicated that there might be a switch from EZH2/EED-independent H3K27me2 to EZH2/EED-dependent H3K27me2.

Considering the low resolution of immunofluorescence detection of H3K27me2, we conducted ULI-NChIP to show the distribution of H3K27me2 more clearly in mouse zygotes and to verify the redundancy of EZH2 on H3K27me2 modification. To distinguish the maternal and the paternal alleles, we collected zygotes by in vitro fertilization using two distinct parental strains, PWK/PhJ (male) and *Ezh2*^*fl/fl*^ (female, C57BL/6J) or *Ezh2*^*fl/fl*^*; Gdf9-Cre* (female, C57BL/6J), containing ~20 million different SNPs between their genome sequences. H3K27me2 possesses distinct features of sequence preference compared with H3K27me3 in mouse zygotes. H3K27me2 was broadly distributed throughout the genome (Fig. 1k, Supplementary Fig. 3a). The level of de novo H3K27me2 on the paternal pronucleus was lower than that on the maternal pronucleus (Fig. 1l, Supplementary Fig. 4). Compared with the DNA methylation pattern, neither the paternal nor maternal genome coincide with 5mC in the enrichment region such as imprinting genes at the PN5 zygotic stage (Supplementary Fig. 3b). The landscape of H3K27me2 was not changed dramatically after maternal EZH2 deletion, and the results are illustrated in Supplementary Fig. 3a, c–f, which shows the distribution of H3K27me2 in different regions of the genome. After EZH2 knockout, H3K27me2 was still mainly distributed in the distal intergenic region without obvious pattern change, which possesses distinct features of sequence preference compared with H3K27me3 in mouse zygotes (Supplementary Fig. 3g, h). However, when comparing the distribution of H3K27me2 at each region, we found that in the maternal genome, the H2K37me2 signal at the promoter and the distal intergenic region was slightly decreased, while at UTR and exon regions, H3K27me2 was slightly increased. In the paternal genome, the H2K37me2 signal at the promoter (≤1 kb), UTR, exon, and distal intergenic region was slightly decreased, while at intron regions, H3K27me2 was slightly increased. This suggests that EZH2 knockout has different effects on different regions of the parental genome.

### Maternal EZH1 is indispensable for H3K27me2 in the paternal pronucleus of Ezh2^m−/p+^ mouse zygotes

**Fig. 2:**
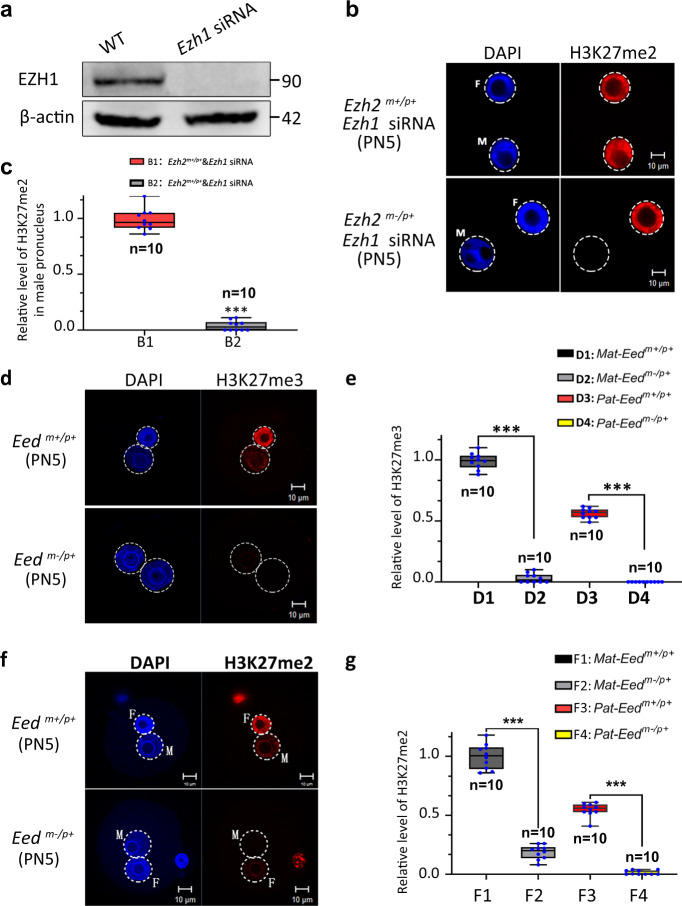
The synergistic effect of Ezh1 and EZH2 on the establishment of de novo H3K27me2 in the male pronucleus. **a** Knockdown efficiency of Ezh1 siRNA microinjected oocytes. Proteins from a total of 100 GV oocytes were loaded for each sample. β-Actin was used as an internal control. **b** Ezh1 siRNA was microinjected before PN2 stage in *Ezh2*^*m−/p+*^ and *Ezh2*^*m+/p+*^ zygotes, and zygotes were used for H3K27me2 immunofluorescent staining at the PN5 stage. M indicates male pronucleus and F indicates female pronucleus in zygote. Scale bar, 10 μm. **c** Relative fluorescence intensity of H3K27me2 in PN5 stage male pronucleus. B1: *Ezh2*^*m+/p+*^ zygotes microinjected with EZH1 siRNA; B2: *Ezh2*^*m−/p+*^ zygotes microinjected with EZH1 siRNA. Error bars, S.E.M. ***P < 2.22745E−16 by two-tailed Student’s *t* tests. Source data are provided as a Source data profile. **d** Maternal loss of EED showed significant loss of H3K27me3 in PN5 stage zygote. Scale bar, 10 μm. **e** Relative fluorescence intensity of H3K27me3 in PN5 stage pronucleus. D1: *Eed*^*m+/p+*^ female pronucleus; D2: *Eed*^*m−/p+*^ female pronucleus D3: *Eed*^*m+/p+*^ male pronucleus; D4: *Eed*^*m−/p+*^ male pronucleus. Error bars, S.E.M. ***P < 4.3406E−19 and *P* < 1.33006E−19 by two-tailed Student’s *t* tests. Source data are provided as a Source data profile. **f** Maternal loss of EED showed significant loss of H3K27me2 in the PN5 stage zygote. M indicates male pronucleus and F indicates female pronucleus in zygote. Scale bar, 10 μm. **g** Relative fluorescence intensity of H3K27me2 in PN5 stage pronucleus. D1: *Eed*^*m+/p+*^ female pronucleus; D2: *Eed*^*m−/p+*^ female pronucleus D3: *Eed*^*m+/p+*^ male pronucleus; D4: *Eed*^*m−/p+*^ male pronucleus. Error bars, S.E.M. ***P < 7.8886E−13 and *P* < 2.92294E−14 by two-tailed Student’s *t* tests. Source data are provided as a Source data profile.The median line of the box plot represents the median, and the top and bottom of the box represent the upper and lower quartile, respectively.

Given that the PRC2 complex is comprised of EED, SUZ12, RbAp48, and a catalytic subunit, either EZH1 or EZH2, we speculated that EZH1 may be indispensable for H3K27me2 in the absence of maternal EZH2. We first investigated the role of EZH1 in mouse zygotes by microinjection of *Ezh1* siRNA. To determine the interference efficiency of *Ezh1* siRNA, we conducted Western blot analysis and real-time PCR. *Ezh1* siRNA significantly reduced the expression of EZH1 (Fig. 2a, Supplementary Fig. 5a). Consistent with our speculation, immunostaining showed that there was complete absence of de novo H3K27me2 in paternal pronuclei of zygotes lacking maternal EZH2 rather than normal zygotes after *Ezh1* siRNA microinjection (Fig. 2b, c). Combining the data of ULI-NChIP, these results suggest that EZH1 might partially safeguard the role of EZH2 to establish de novo H3K27me2 on the paternal pronucleus at the PN5 zygotic stage.

Next, we investigated the possible relationship between EZH1 and EZH2 on H3K27me2 in mouse zygotes through conditional inactivation of maternal EED, the essential core subunit of PRC2, in oocytes. We tested the protein level of EED in oocytes of maternal knockout EZH2 and found that it did not significantly decrease (Supplementary Fig. 5b). Thereafter, we crossed conditional *Eed*^*fl/fl*^ mice with *Gdf9-Cre* transgenic lines to generate embryos that were maternally deficient for EED in oocytes (*Eed*^*fl/fl*^*; Gdf9-Cre*) and lacked detectable EED protein in zygotes (*Eed*^*m−/p+*^) (Supplementary Fig. 5c). Zygotes lacking maternal EED showed significant loss of H3K27me3 and H3K27me2 in the male pronucleus (Fig. 2d–g). Meanwhile, *Eed*^*fl/fl*^*; Gdf9-Cre* female mice showed reduced litter size in comparison to *Eed*^*fl/fl*^ female mice (Supplementary Table 1). Combined with our Western blot analysis result that EZH1 was only expressed at stages from GV oocytes to zygotes (Fig. 1a), we propose that Ezh1 might partially indispensable to the establishment of de novo H3K27me2 in the paternal pronucleus, and this effect is stage-specific.

### H3K27 methylation modification is cell cycle-dependent

**Fig. 3:**
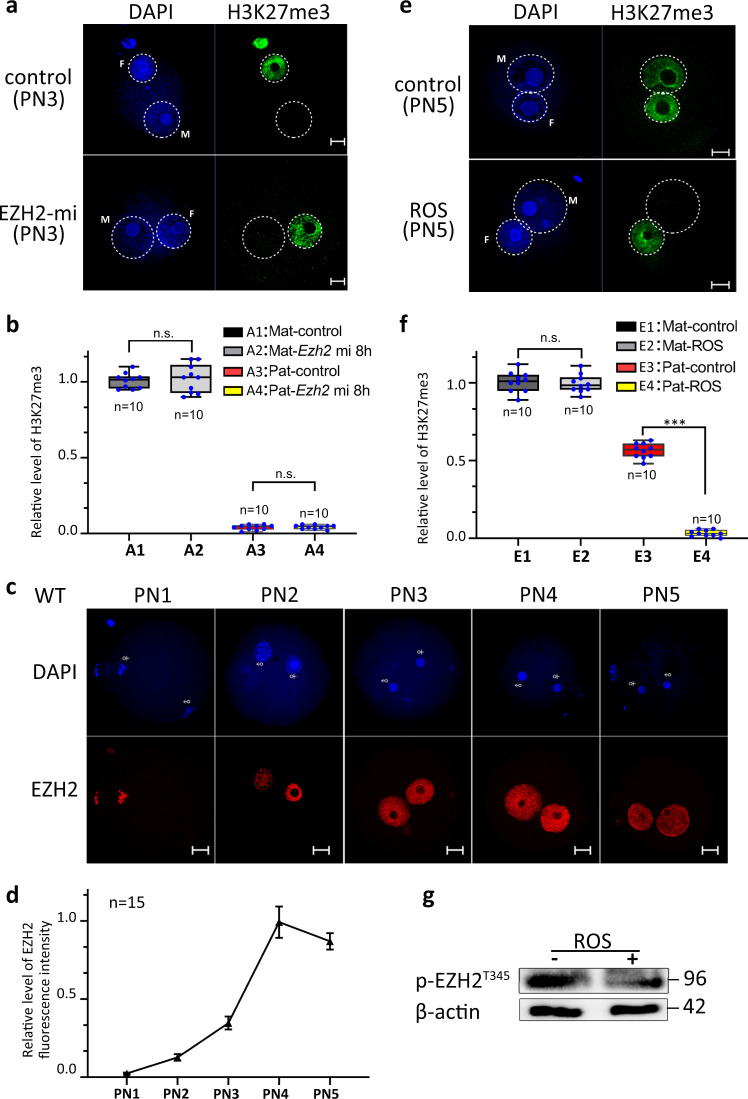
Cell cycle-dependent establishment of H3K27me3 in the male pronucleus. **a** The H3K27me3 state in pronucleus at 8 h of IVF after *Ezh2* mRNA microinjection. M indicates male pronucleus and F indicates female pronucleus in zygote. Scale bar, 10 μm. **b** Relative fluorescence intensity of H3K27me3 in PN3 stage pronucleus after *Ezh2* mRNA microinjection. A1: Female pronucleus in control zygote; A2: Female pronucleus in *Ezh2* mRNA microinjected zygote; A3: Male pronucleus in control zygote; A4: Male pronucleus in *Ezh2* mRNA microinjected zygote. Error bars, S.E.M. *P* > 0.6796 and *P* > 0.6780 by two-tailed Student’s *t* tests. n.s. represents nonsignificant difference. Source data are provided as a Source data profile. **c** The localization of EZH2 in zygotes from PN1 to PN5 stages. Scale bar, 10 μm. **d** The relative level of EZH2 fluorescence intensity from PN1 to PN5 stages. *n* = 15 embryos at each stage examined over three independent experiments. Error bars, mean ± S.E.M. Source data are provided as a Source data profile. **e** The H3K27me3 state in pronucleus at 13 h of IVF after roscovitine treatment. The addition of roscovitine inhibited the establishment of H3K27me3 in the PN5 stage male pronucleus. M indicates male pronucleus and F indicates female pronucleus in zygote. Scale bar, 10 μm. **f** Relative fluorescence intensity of H3K27me3 in PN5 stage pronucleus after roscovitine treatment. E1: Female pronucleus in control zygote; E2: Female pronucleus in roscovitine treated zygote; E3: Male pronucleus in control zygote; E4: Male pronucleus in roscovitine treated zygote. Error bars, S.E.M. ***P < 2.05216E−17. n.s. represents a nonsignificant difference. *P* > 0.6963 by two-tailed Student’s *t* tests. Source data are provided as a Source data profile. **g** Western blot shows that the protein level of p-EZH2^T345^ was decreased after roscovitine treatment. The median line of the box plot represents the median, and the top and bottom of the box represent the upper and lower quartile, respectively.

Since there was no H3K27me2 and H3K27me3 before the PN5 stage in the paternal pronucleus of mouse zygote, we asked whether overexpression of EZH2 in mouse zygote would cause H3K27me3 modification in the paternal pronucleus before the PN4 stage. Overexpression of EZH2 (Supplementary Fig. 5d, e) had no effect on de novo H3K27me3 in the paternal pronucleus at the PN3 stage (Fig. 3a, b). It was shown that EZH2 was localized to both maternal and paternal pronuclei at the PN3 stage (Fig. 3c, d), suggesting that the absence of H3K27me3 before PN4 is not caused by lacking EZH2 on the paternal genome. Since PN4-PN5 represents the G2/M transition in mouse zygotes, we asked whether de novo H3K27 methylation is regulated by the cell cycle. Previous studies have reported that CDK1 can phosphorylate EZH2, while CDK1 is responsible for the G2/M transition. To verify this, zygotes were treated with roscovitine, a CDK1 inhibitor, and we found that this treatment resulted in almost complete disappearance of H3K27me3 in the paternal pronucleus (Fig. 3e, f). Immunoblotting showed that inhibition of CDK1 resulted in a significant decrease in the EZH2 phosphorylation level (Fig. 3g).

### EHMT1, but not EHMT2, is indispensable for H3K27me2 in mouse zygotes

**Fig. 4:**
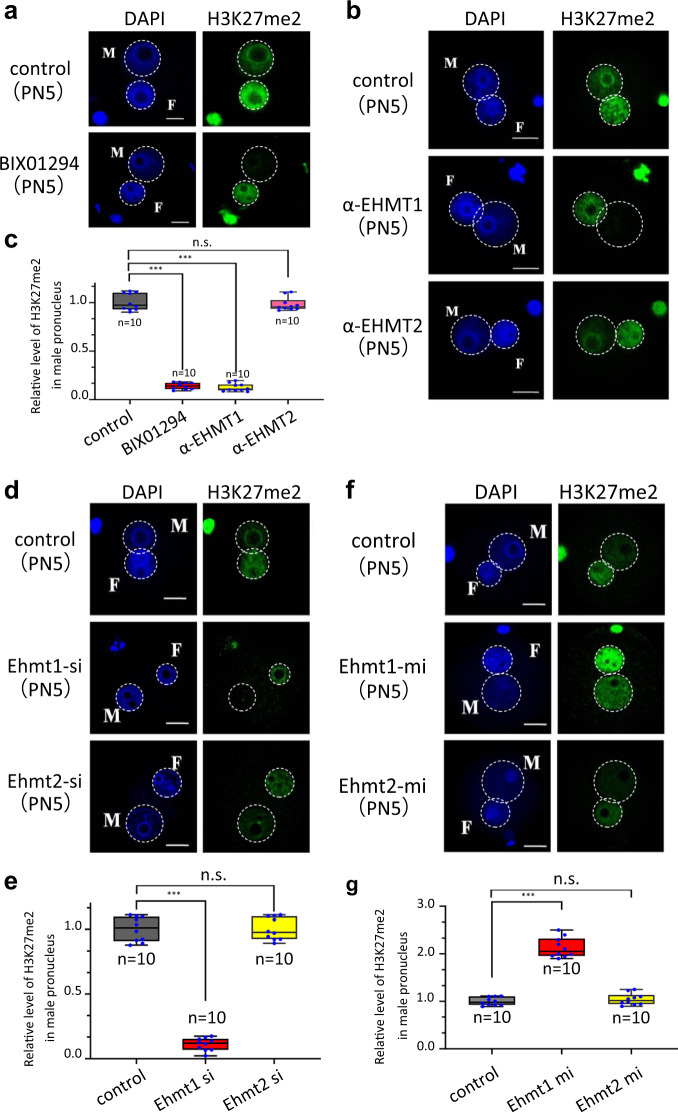
The H3K27me2 state in PN5 zygotes treated with different agents at 13 h of IVF. Confocal micrographs show the immunostained H3K27me2 (green) and DNA (DAPI, blue) in mouse zygotes. **a** Zygotes were cultured in KSOM medium containing 10 μM BIX01294. Control group was cultured in KSOM medium containing DMSO. M indicates male pronucleus and F indicates female pronucleus in zygote. Scale bar, 10 μm. **b** The H3K27me2 state in zygotes at 13 h of IVF after EHMT1 or EHMT2 antibody microinjection. α-EHMT1 and α-EHMT2 indicate mouse zygotes cultured in KSOM medium after antibody microinjection before PN3 stage. M indicates male pronucleus and F indicates female pronucleus in zygote. Scale bar, 10 μm. **c** Relative fluorescence intensity of H3K27me2 in PN5 male pronucleus after treatment with 10 μM BIX01294, α-EHMT1, and α-EHMT2. Error bars, S.E.M. ***P < 1.36095E−16 and *P* < 1.49664E−16. n.s. represents nonsignificant difference. *P* > 0.4948 by two-tailed Student’s *t* tests. Source data are provided as a Source data profile. **d** The H3K27me2 state of zygotes at 13 h of IVF after EHMT1 or EHMT2 siRNA microinjection. Control group was microinjected with scrambled control siRNA. M indicates male pronucleus and F indicates female pronucleus in zygote. Scale bar, 10 μm. **e** Relative fluorescence intensity of H3K27me2 in PN5 male pronucleus after microinjected with *Ehmt1* or *Ehmt2* siRNA. Error bars, S.E.M. ***P < 7.10683E−16. n.s. represents a nonsignificant difference. *P* > 0.9651 by two-tailed Student’s *t* tests. Source data are provided as a Source data profile. **f** The H3K27me2 state in zygotes at 13 h of IVF after *Ehmt1* or *Ehmt2* mRNA microinjection. M indicates male pronucleus and F indicates female pronucleus in zygote. Scale bar, 10 μm. **g** Relative fluorescence intensity of H3K27me2 in PN5 male pronucleus after microinjected with *Ehmt1* or *Ehmt2* mRNA. Error bars, S.E.M. ***P < 5.508E-−12. n.s. represents a nonsignificant difference. *P* > 0.3577 by two-tailed Student’s *t* tests. Source data are provided as a Source data profile. The median line of the box plot represents the median, and the top and bottom of the box represent the upper and lower quartile, respectively.

Given that the EHMT1/EHMT2 complex shares an important number of common genomic loci with PRC2 and that it can recruit PRC2 to genomic loci to catalyze H3K27 di-methylation and trimethylation (H3K27me2/3) in mESCs^14^, we chose BIX01294 or UNC0638, selective inhibitors of EHMT1 or EHMT2 histone methyltransferase without effect on SUV39H1 and PRMT1, to analyze the function of EHMT1/EHMT2 on paternal pronucleus H3K27me2. We found that there was nearly an absence of de novo H3K27me2 in the paternal pronucleus after inhibitor treatment, which demonstrates that EHMT1 or/and EHMT2 is involved in de novo H3K27me2 in the paternal pronucleus (Fig. 4a, c, Supplementary Fig. 6a, b). To further confirm whether EHMT1 or EHMT2 is responsible for this, EHMT1 or EHMT2 antibody microinjection assay was conducted. The paternal pronuclear H3K27me2 was nearly lost after injection of anti-EHMT1 antibody (α-EHMT1) in mouse zygotes. On the contrary, the injection of anti-EHMT2 antibody (α-EHMT2) had no effect on paternal pronuclear H3K27me2 (Fig. 4b, c). To further investigate the role of EHMT1 and EHMT2 in de novo H3K27me2 in mouse zygotes, we knocked down EHMT1 or EHMT2 by injection of their specific siRNA. The interference efficiency of *Ehmt1* and *Ehmt2* siRNA was detected by Western blot (Supplementary Fig. 6c, d). In the *Ehmt2* knockdown group, the H3K27me2 level was similar to that of the control group, while in the *Ehm1* knockdown group, H3K27me2 was nearly lost in the paternal pronucleus (Fig. 4d, e). Consistent with this, overexpression of EHMT1 rather than EHMT2 significantly increased the H3K27me2 level in the paternal pronucleus (Fig. 4f, g). Consistent with the above in vitro results, maternal deficiency of *Ehmt2* had no effect on the H3K27me2 (Supplementary Fig. 6e, f). These comprehensive in vivo and in vitro results demonstrate that EHMT1 instead of EHMT2 is required for H3K27me2 in the paternal pronucleus of mouse zygotes.

### H3K27me2 might be the substrate of de novo H3K27me3 formation through EHZ2 in the male pronucleus of mouse zygotes

**Fig. 5:**
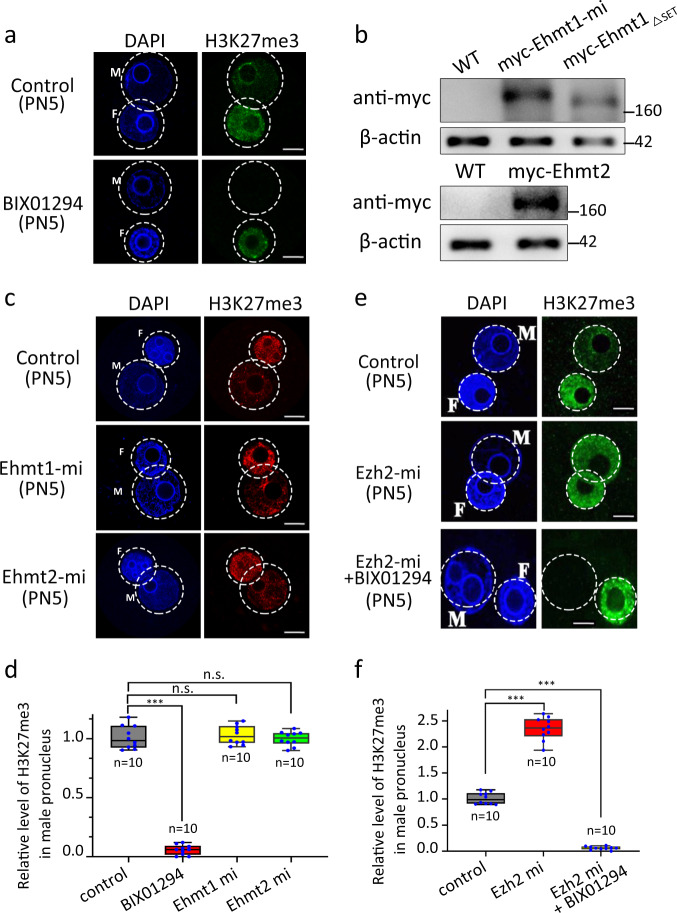
The H3K27me3 signal in PN5 zygotes treated with different agents at 13 h of IVF. **a** Zygotes were cultured in KSOM medium containing 10 μM BIX01294. Control group was cultured in KSOM medium containing DMSO. M indicates male pronucleus and F indicates female pronucleus in zygote. Scale bar, 10 μm. **b** The upper figure shows the protein levels of EHMT1 in zygotes at 13 h of IVF after EHMT1 full-length (*Ehmt1*) and SET domain deleted EHMT1 truncated mRNA (*Ehmt1*_*ΔSET*_) microinjection, respectively. The bottom figure shows the protein levels of EHMT2 in zygotes at 13 h of IVF after EHMT2 full-length (*Ehmt2*) mRNA microinjection. **c** The H3K27me3 state in zygotes at 13 h of IVF after *Ehmt1* or *Ehmt2* mRNA microinjection. M indicates male pronucleus and F indicates female pronucleus in zygote. Scale bar, 10 μm. **d** Relative fluorescence intensity of H3K27me3 in PN5 male pronucleus after treatment with BIX01294, *Ehmt1,* and *Ehmt2* mRNA. Error bars, S.E.M. ***P < 2.10148E−16. n.s. represents a nonsignificant difference. *P* > 0.6153 and *P* > 0.7802 by two-tailed Student’s *t* tests. Source data are provided as a Source data profile. **e** The H3K27me3 state in zygotes at 13 h of IVF after Ezh2 mRNA microinjection. Ezh2-mi +BIX01294 indicates zygotes cultured in KSOM medium containing BIX01294 after ezh2 mRNA microinjection. White dashed circles indicate the male pronucleus (M) and female (F) pronucleus. Scale bar, 10 μm. **f** Relative fluorescence intensity of H3K27me3 in PN5 male pronucleus after different treatment strategies. Error bars, S.E.M. ***P < 1.25007E−12 and *P* < 1.10945E−15 by two-tailed Student’s *t* tests. Source data are provided as a Source data profile. The median line of the box plot represents the median, and the top and bottom of the box represent the upper and lower quartile, respectively.

Next, we investigated the function of EHMT2 and EHMT1 in paternal pronuclear H3K27me3 establishment. Firstly, mouse zygotes treated with BIX01294 showed loss of de novo H3K27me3 in the paternal pronucleus, suggesting that EHMT2 and/or EHMT1 may participate in the de novo H3K27me3 formation (Fig. 5a, d). Secondly, to clarify either or both of EHMT2 and EHMT1 functions, we used a “gain-of-function” approach to examine the effect of mRNA microinjection. The protein level of EHMT2 and EHMT1 significantly increased at 6 h of microinjection as detected by Western blotting (Fig. 5b). Overexpression of either EHMT1 or EHMT2 did not obviously increase the immunostaining signal of H3K27me3 in mouse zygotes (Fig. 5c, d). On the other hand, overexpression of EZH2 leads to increased H3K27me3 modification (Fig. 5e, f). BIX01294 treatment, however, could prevent this process (Fig. 5e, f). Collectively, these results indicate that de novo H3K27me3 modification in the paternal pronucleus should use H3K27me2 as a substrate.

### The SET domain of EHMT1 is indispensable for de novo H3K27me2 in mouse zygotes

**Fig. 6:**
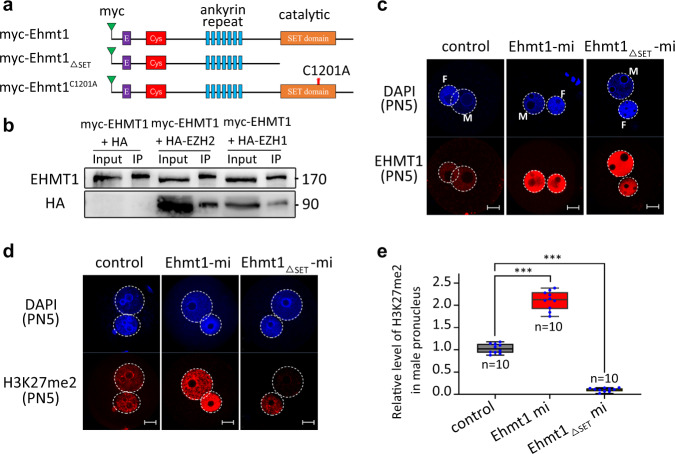
Overexpression of SET domain deleted EHMT1 truncated mRNA inhibits the establishment of H3K27me2 in the male pronucleus. **a** Schematic representation of myc-tagged mEHMT full-length, myc-tagged mEHMT SET domain deleted protein, and myc-tagged mEHMT containing the point mutant (C1201A) in SET domain without catalytic activity. **b** The interaction of EHMT1 with EZH1 and EZH2 proteins were examined by Co-IP. EHMT1-HA was co-transfected into HEK293T cells with EZH1-Myc, EZH2-Myc or Myc-Vector, respectively. Anti-HA was used to precipitate EHMT1 and associated proteins. Immunoblots were probed with EHMT1 and HA antibodies to detect the pull-down proteins (EZH1, EZH2) and EHMT1-HA (positive control), respectively. **c** Immunostaining for the EHMT1 in zygotes at 13 h of IVF after EHMT1 full-length (*Ehmt1*) and SET domain deleted EHMT1 truncated mRNA (*Ehmt1*_*ΔSET*_) microinjection. DNA was counterstained with DAPI. M indicates male pronucleus and F indicates female pronucleus in zygote. Scale bar, 10 μm. **d** The H3K27me2 state of zygotes at 13 h of IVF after EHMT1 full-length (*Ehmt1*) and SET domain deleted mRNA (*Ehmt1*_Δ*SET*_) microinjection. Scale bar, 10 μm. **e** Relative fluorescence intensity of H3K27me2 in PN5 male pronucleus after different treatment strategies. *Ehmt1*mi indicates zygotes microinjected with *Ehmt1* full-length mRNA; *Ehmt1*_Δ*SET*_ mi indicates zygotes microinjected with *Ehmt1* SET domain deleted mRNA. *n* = 10 zygotes at each group examined over three independent experiments. Error bars, S.E.M. ***P < 2.40369E−11and *P* < 1.20055E−15 by two-tailed Student’s *t* tests. Source data are provided as a Source data profile. The median line of the box plot represents the median, and the top and bottom of the box represent the upper and lower quartile, respectively.

The results so far indicated that EHMT1 but not EHMT2 was required for H3K27me2. We next set out to investigate a possible interplay between EHMT1 and EZH proteins. To investigate this question, we constructed two plasmids expressing myc-EHMT1 fusion protein, myc-EHMT1_△SET_ lacking their C-terminal catalytic domains, respectively (Fig. 6a).

We detected the interaction between EHMT1 and EZH proteins by Co-immunoprecipitation (Co-IP) in cells co-expressing the two proteins (EHMT1 and EZH1 or EHMT1 and EZH2), confirming that EHMT1 does interact with PRC2-EZH1 and/or PRC2-EZH2 (Fig. 6b). Then we examined whether the SET domain of EHMT1 was required for de novo H3K27me2 in mouse zygotes. Through Co-IP experiments, we found that EHMT1_△ SET_ could still interplay with EZH proteins (Supplementary Fig. 7a). However, in the zygotes of EHMT1_△SET_ (Fig. 6c), EZH2 could not localize to the paternal pronucleus normally (Supplementary Fig. 7b). In addition, there was a virtually complete disappearance of de novo H3K27me2 and H3K27me3 in the paternal pronucleus after EHMT1_△SET_ overexpression (Fig. 6d, e, Supplementary Fig. 7c, d). In addition, in order to further analyze whether the catalytic activity of EHMT1 was necessary for the de novo H3K27me2 in zygotes, we constructed a point mutation EHMT1^C1201A^ (Fig. 6a), which contains the mutant SET-domain (C1201A) showing no enzymatic activity, but it still can form a heterodimeric complex with EHMT2^17^. Consistent with the full-length EHMT1 zygotes, the level of H3K27me2 in the zygotes of EHMT1^C1201A^ increased significantly (Supplementary Fig. 7g, h), while H3K27me3 did not change significantly (Supplementary Fig. 7d, f). Therefore, we suggest that SET domain of EHMT1 rather than its catalytic activity is necessary for de novo H3K27me2 in mouse zygotes.

## Discussion

In mice, the oocyte is fertilized within one hour, and the paternal genome undergoes intense chromosomal reprogramming. During the last few years, intensive researches have shed light on the genomic landscapes of representative epigenetic modifications. However, the establishment and regulation of epigenetic modifications remain poorly understood.

The depletion of *Ezh2* in highly proliferating fetal stem cells resulted in the failure of hematopoiesis and cardiogenesis even in the presence of EZH1^18^, indicating the complementation between EZH1 and EZH2 is cellular context-dependent. It is still not clear how EZH1 and EZH2 coordinate to regulate H3K27 methylation and transcription during development. Consistent with the phenotypic difference between *Ezh2* and *Eed* maternal knockout mice^1–3^, we proved that maternal ablation of EZH2 in zygotes leads to loss of H3K27me3, but not so for H3K27me2. Besides, our study revealed that EZH1 could partially safeguard the function of EZH2 on the establishment of de novo H3K27me2 rather than H3K27me3 in the paternal pronucleus of mouse zygotes. Future studies should address the reason. Previous studies showed that PRC2-EZH1 exhibited an obviously lower HMT activity than that of PRC2–EZH2^5,19,20^. Lee et al.^19^ then showed that automethylation of EZH1 was distinctly weaker compared to that of EHZ2. Our present study revealed that EZH2 is phosphorylated by the cyclin-dependent kinase CDK1 in mouse zygotes. Whether there is a corresponding phosphorylation modification in EZH1 to affect H3K27 methylation and how these two post-translational modifications interact in mouse zygotes are very interesting topics for future study.

A previous study hypothesized H3K27me2 as a substrate for subsequent H3K27me3 formation^21^. However, it remains unclear what is the relationship between H3K27me2 and H3K27me3 in mouse zygotes. In this study, we revealed that H3K27me2 was broadly distributed throughout the genome, which showed distinct features of sequence preference compared with H3K27me3. Meanwhile, our findings clearly documented that H3K27me2 might be an essential prerequisite for the subsequent de novo H3K27me3 modification in the male pronucleus. Notably, the latest report by Samuel demonstrates that some parental-specific early domains coincide with H3K27me3, which are maintained up to the eight-cell stage^22^. Based on our study, there was a transition from EZH2/EED-independent H3K27me2 to EZH2/EED-dependent H3K27me2 during preimplantation development. Future investigations of H3K27 methylation during pre-implantation will likely elucidate the accurate time undergoing this switch and the mechanisms controlling this important physiological event through optimized technologies. Furthermore, future studies need to address whether there is a possible connection between these two switches.

Another interesting aspect of our study is that only EHMT1 but not EHMT2 participates in the regulation of H3K27me2 in mouse zygotes. It was shown that the EHMT2/EHMT1/WIZ complex can recruit PRC2 (which is responsible for catalyzing H3K27 methylation) to the corresponding site for H3K27 methylation modification in mouse ES cells^14^, suggesting that the EHMT2/EHMT1/WIZ complex may be involved in the regulation of H3K27me2 and H3K27me3 modification in mouse zygotes. WIZ is essential for the stability of the EHMT2/EHMT1 complex. How the genomic regions targeted by PRC2 are methylated remains poorly understood. Our previous results showed that there was no WIZ protein in mouse oocytes and zygotes^23^, which not only suggests that EHMT2 and EHMT1 do not function as complexes in mouse zygotes, but also that H3K27me2 is likely to have a very unique modification pattern and regulatory mechanism in mouse zygotes. Although further studies can address how EHMT1 recruits PRC2 to specific target sites in the genome using other approaches, it is worth noting that EHMT1_△SET_ demonstrated the importance of the SET domain, and EHMT1^C1201A^ point mutant (unable binding cofactor SAM) revealed that the SAM binding site of EHMT1 is not necessary for recruiting PRC2. In addition, UNC0638 and BIX01294 compete with the peptide substrate but not with the cofactor SAM. Kubicek et al.^24^ reported that EHMT1 is capable of catalyzing formation of H3K9me3 in an in vitro methyltransferase assay. Lu et al.^25^ recently reported that EHMT1 catalyzes H4K16me1 in response to DNA damage in an ATM-dependent manner, which suggests EHMT1 has a possible role in regulating H3K27me3 through other functions. Therefore, it remains possible that the effect for H3K27me3 is not through direct modification of H3K27me2 but through other functions of EHMT1, like the H3K9me1, H3K9me2 or H3K9me3. Future studies are needed to place emphasis on this open question to verify our conclusions.

**Fig. 7:**
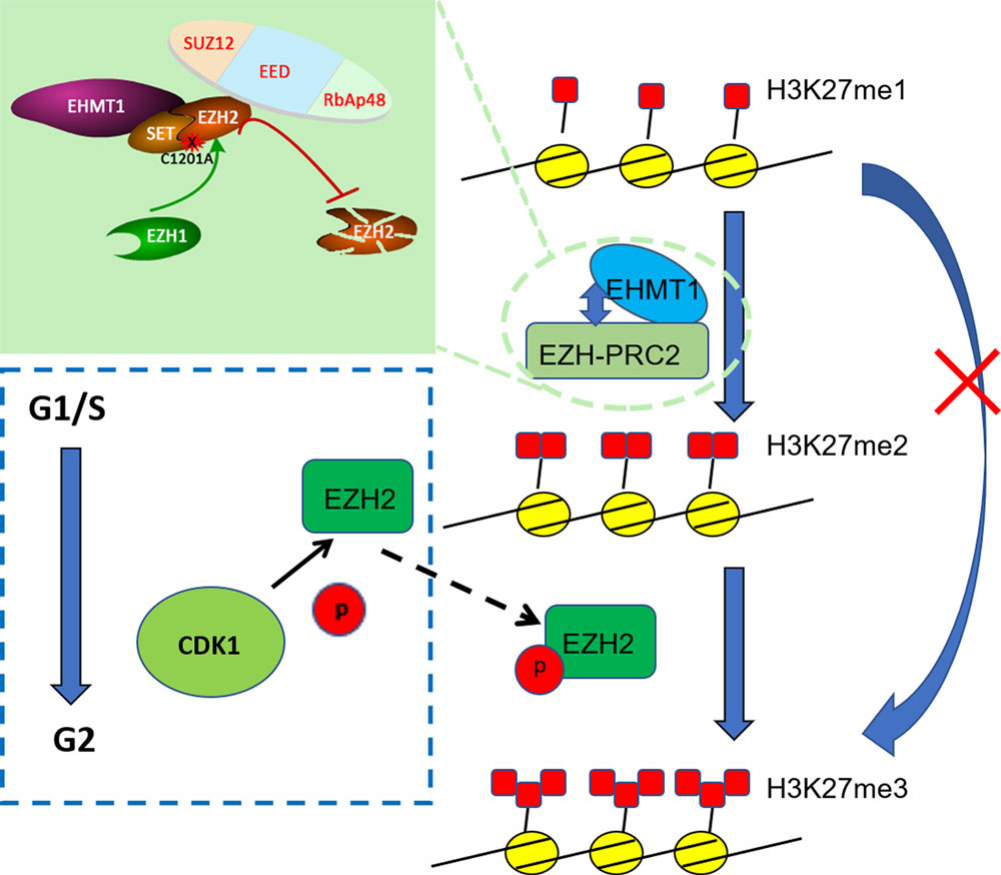
Schematic figure showing possible mechanism of H3K27me2 and H3K27me3 establishment in mouse zygotes. We propose that EHMT1 is a new contributor to the formation of H3K27me2, and H3K27me2 might be a necessary prerequisite for the re-modification of H3K27me3 in the male pronucleus. The SET domain rather than the catalytic activity of EHMT1 is indispensable for de novo H3K27me2 in mouse zygotes. In addition, EZH2 is indispensable for the formation of H3K27me3 in mouse zygotes, and EZH2 is regulated by CDK1 in a cell cycle-dependent manner. However, Ezh1 and EZH2 have a synergistic effect on the establishment of de novo H3K27me2 in the paternal pronucleus of mouse zygote.

Our work points to distinct mechanisms on the establishment and regulation of de novo H2K27me2 and H2K27me3 in zygotes (Fig. 7), with broad implications for our understanding of epigenetic reprogramming in general.

## Methods

### Ethics statement

All experiments and methods in this study were conducted in accordance with the guidelines of the Ethics and Experimental Animal Committee of the Institute of Zoology, Chinese Academy of Sciences, China.

### Mice

The oocyte-specific mutant mice with the deletion of *Ezh2* exon 4 were generated by crossing *Ezh2*^*flox/flox*^ mice^16,26^ with transgenic mice expressing *Gdf9* promoter mediated Cre recombinase. The resulting *Ezh2*^*flox/+*^*; Gdf9-Cre*+ male mice were then crossed with female mice homozygous for the *Ezh2* conditional allele, *Ezh2*^*Flox/Flox*^. *Ezh2*^*flox/flox*^*; Gdf9-Cre* female mice were used as the experimental group, while *Ezh2*^*Flox/Flox*^ female mice were used as the control group. For simplicity, they are referred to as *Ezh2* knockout and control mice hereafter. Genotypes were determined by PCR amplification of mouse tail DNA samples (Supplementary Table 2). qRT-PCR and Western blot analyses were used to confirm the complete elimination of *Ezh2* transcript and protein in *Ezh2* KO GV oocytes. Primers used in this paper are listed in supplemental materials. Mice were maintained on a C57Bl/6 J genetic background. Conditional G9a (*G9a*^*fl/fl*^) mice^27^ and conditional Eed (*Eed*^*fl/fl*^) mice^28^ were also used. The mating strategy for maternal knockout of G9a and EED is the same as that of EZH2. Mice were housed in controlled environmental conditions with 12-h alternating light/dark cycles, with the temperature controlled at 23~25 °C, the humidity controlled at 40~65%, and free access to water and food supplies, as approved by the ethics committee for animal care of the Institute of Zoology, Chinese Academy of Sciences.

### Collection and in vitro maturation of oocytes

Fully grown GV stage oocytes were physically isolated from ovaries of 6- to 8-week-old female ICR mice in pre-warmed M2 culture medium (Sigma) supplemented with 200 μM of 3-isobutyl-1-methylxanthine (IBMX, Sigma) to prevent them from undergoing GVBD. Following specific experimental treatment, oocytes were washed thoroughly, and cultured in pre-warmed M2 medium to different stages.

### In vitro fertilization

Six- to eight-week-old mice were superovulated by injecting 5 IU pregnant mare serum gonadotropin (PMSG) and 5 IU human chorionic gonadotropin (HCG) after 48 h. Superovulated metaphase II-arrested (M II) oocytes were harvested from the ampullae of the oviducts in human tubal fluid (HTF) medium at 14 h post-HCG. The oocytes were inseminated with capacitated spermatozoa, which were collected from the caudal epididymis of 10-week old ICR males, followed by incubation in HTF medium for 30 min at 37 °C in an atmosphere of 5% CO_2_. Three hours later, cumulus cells were dispersed by sperm, and zygotes (1-cell embryos) were thoroughly washed and transferred in KSOM medium.

### Collection and in vitro development of early embryos

To synchronize in vitro embryo development, zygotes and cleavage stage embryos were collected at the indicated hours: PN5 zygotes were collected at 27 h post-hCG injection, 2-, 4-, 8-cell embryos, and blastocysts were harvested at 48, 60, 72, 120 h post hCG injection.

### Construction of plasmids for *Ehmt1* and in vitro transcription of mRNA

For the production of mRNAs, *Ehmt1* full-length, *Ehmt1*_*△SET,*_ and *Ehmt1*^*C1201A*^ sequences were cloned to the pCS2+ vector, including 6 × myc epitopic tags. Coding sequences were PCR-amplified from the constructed plasmids with primers containing the T7 promoters, and the DNA products were used as templates to generate mRNA with mMESSAGE mMACHINE T7 Transcription Kit (Ambion; Am1344). Poly (A) Tailing Kit (Ambion) was used for the production of capped and tailed mRNA. All mRNA products were purified by the RNeasy Mini Kit (Qiagen) according to the provided protocol. The concentration of *Ehmt1* full-length and SET-delete mRNA was determined with Nanodrop Spectrophotometer and then diluted to a final concentration of 1 μg/μL for mRNA over-expression experiments.

### Cytoplasmic injection of siRNA, antibodies or mRNA

For *Ezh1* knockdown in mouse oocytes, *Ezh1* stealth siRNA 5′–3′ (synthesized by Thermo fisher) (Supplementary Table 3) was diluted to a final concentration of 20 μM. The same amount of scrambled siRNA was used as control. Each oocyte was microinjected with 10 pg of siRNA. All siRNAs were diluted with nuclease-free water (Invitrogen) and stored in −20 °C. The final concentration of oligonucleotides was 20 μM. After microinjection, the GV oocytes were arrested in M2 medium containing 200 μM IBMX for 24 h for the depletion of *Ezh1* transcript. To conduct the following ICSI experiments, microinjected GV oocytes were inhibited for 12 h, and then washed in IBMX-free M2 medium for at least four times to thoroughly remove the inhibitor, and then cultured for 12 h following intracytoplasmic sperm injection (ICSI). Zygotes were collected and cultured in KSOM medium to different developmental stages.

For antibody microinjection, rabbit anti-Ezh1 (Abcam, ab176115) antibody was microinjected into cytoplasm of in vitro zygotes at 2~4 h. The boiled antibody was used as a negative control.

For zygote mRNA overexpression, 1 μg/μL mRNA solution was microinjected into the cytoplasm of PN2 stage zygotes. Microinjected zygotes were cultured in M2 medium and collected at the PN5 stage for specific experiments. For GV oocyte mRNA overexpression, mRNA was microinjected into the cytoplasm of fully grown GV oocytes in M2 medium containing 200 μM IBMX, and cultured for 4 h followed by immunofluorescence.

### Antibodies and peptide

The following primary antibodies were used, respectively: Mouse anti-Ezh2 antibody (BD Bioscience; 1:200), rabbit anti-Ezh1 antibody (Abcam; ab176115), rabbit anti-EHMT1 antibody (Abcam; ab41969), rabbit anti-H3K27me2 antibody (for IF, Cell Signaling, 9728S), another rabbit anti-H3K27me2 antibody (for IF, Mybiosource, MBS126234), rabbit anti-H3K27me3 antibody (for IF, Cell Signaling, 9733S), antibodies were diluted by 1% BSA/PBS. anti-H3K27me2 (for ULI-NChIP, Actif Motif; 61435). H3K27me2 peptide (Diagenode, C16000046-50) Accordingly, the following secondary antibodies were used: goat anti-mouse IgG(H + L) Alexa Fluor 488 (Invitrogen; A-11001; 1:1000); goat anti-rabbit IgG(H + L) Alexa Fluor 488 (Invitrogen; A-11008; 1:1000); goat anti-mouse IgG(H + L) Alexa Fluor 594 (Invitrogen; R37121; 1:1000); goat anti-rabbit IgG(H + L) Alexa Fluor 594 (Invitrogen; A-11012; 1:1000). Antibodies were diluted by 1% BSA/PBS. Each experiment was repeated at least three times.

### Inhibitor treatment

Inhibitors were prepared as 50 mM stock solutions in DMSO and stored at −20 °C. Zygotes were collected and transferred into KSOM medium containing BIX 01294 (5 μM, Sigma, B9311), unc0638 (10 μM, Sigma, U4885) and Roscovitine (ROS, 200 μM, Sigma, R7772). Zygotes cultured in KSOM medium containing equivalent DMSO were used as control.

### Western blot

Western blot analysis of GV oocytes or early embryos was performed using standard procedures. In brief, a total of 150 GV oocytes or early embryos were collected and boiled in sodium dodecyl sulfate (SDS) sample buffer for 5 min. The boiled proteins were separated by SDS-PAGE and then electrically transferred to PVDF membranes. The blots were probed with respective primary antibodies at an appropriate dilution by overnight incubation at 4 °C, followed by 1-h incubation with appropriate HRP-conjugated secondary antibodies at room temperature.

### Immunofluorescence and imaging

Oocytes and embryos were washed in M2 medium and fixed in 4% paraformaldehyde in PBS for 30 min, permeabilized for 20 min in 0.1% Triton X-100 in PBS, and then blocked in PBS containing 1 mg/ml BSA(PBS/BSA) for 1 h at room temperature. After blocking, the cells were stained with respective primary antibodies overnight at 4 °C. After washing three times with PBS/BSA, the cells were incubated for 1 h with specific fluorescent secondary antibodies at room temperature, followed by incubation with Hoechst 33342 for 20 min. These cells were mounted on glass slides and examined with a Zeiss LSM 780 confocal laser-scanning microscope.

### ULI-NChIP-seq library generation and sequencing

A step-by-step protocol describing the differentiation protocol can be found at Protocol Exchange^29^. For H3K27me2 ULI-NChIP, 300 zygotes without polar body were collected in Nuclei Extraction Buffer directly and sheared by micrococcal nuclease (MNase) at 25 °C for 5 min. Then the samples were incubated with H3K27me2 antibody overnight under constant rotation on a rotator at 4 °C. The next day, 10 µl Dyna beads Protein A (Thermo Fisher Scientific) was added to each sample and incubated 2 h up to overnight at 4 °C. Subsequently, the beads were washed twice with low salt washing buffer, twice high salt washing buffer. For each sample, 100 µl elution buffer was added to resuspend beads and incubated at 65 °C for 2 h to elute DNA from the beads. The DNA was purified by phenol:chloroform:isoamyl alcohol method. The sample was centrifugated at 13,000 × *g* at room temperature, the supernatant was transferred to a new 1.5 ml tube, followed by the addition of 3 M NaOAc and LPA (Roche No. 10901393001, and finally ice-cold 100% EtOH was added. The above was mixed well by vertex, and the tube was put at −20 °C to precipitate for 30 min up to overnight; the DNA was centrifugated at 13,000 × *g* for 30 min at 4 °C, the supernatant was removed, and 70% EtOH was finally added. The tube was kept for 5 min to allow the salt to dissolve, centrifuged at 13,000 × *g* for 5 min at 4 °C to attach the pellet to the bottom, and the EtOH was removed to let the pellet dry at room temperature. Finally, 30 µl DNA elution buffer was added. Then samples were subjected to the ULI- NChIP library preparation. ULI-NChIP library was generated using the KAPA Hyper Prep Kit according to the manufacturer’s protocol. Paired-end 150-bp sequencing was performed on a HiSeq2500.

### ULI-NChIP-seq analysis

The raw reads were processed by Trimmomatic (version 0.38) to cut adapters and to trim low-quality reads with the minimal length 90 bp and minimal quality of bases 20. Clean reads were mapped to the mouse mm10 genome by Bowtie2 with parameters as described previously and the default parameters in zygotes and MII ChIP-sequencing data, respectively. High-quality unique mapping was performed by Samtools with MAPQ more than 30 and self-coded Perl scripts. The duplication reads were removed by Picard to obtain the non-redundant reads. SNP split was utilized to split the maternal and paternal reads alignment with the allele-specific C57BL6 and PWK mm10 genome. SICER was utilized to call peaks (windows 200, Gap 600, *q* value 1e−5) with SICER.sh module and the differential peaks were found with SICER-df.sh module^30^. The peaks filtered by length more than 1000 bp and fold change more than 5 were annotated by Chip Seeker for gene category analysis and Cluster profiler for gene function annotation such as KEGG and GO analysis. The depth and coverage of ChIP-sequencing data were calculated by Bed tools with the 5 kb windows and self-coded python scripts, respectively, which were visualized by ggpubr and ggplot packages in R with the Wilcoxon rank test.

### Reporting summary

Further information on research design is available in the Nature Research Reporting Summary linked to this article.

## Supplementary information

Supplementary Information

Peer Review File

Reporting Summary

## Data Availability

The generated and analyzed datasets in the current study are available in the Gene Expression Omnibus with an accession number GSE134592. The processing codes are following the documentations of each software, and all other relevant materials are available on request. Source data are provided with this paper.

## Source data

Source Data

## Code availability

All code related to this article are available in https://github.com/Daisyzhouqian/ChipSeqAnalysisForNC.
