# Supplementary material for: PRC2 and EHMT1 regulate H3K27me2 and H3K27me3 establishment across the zygote genome
Source: Nat Commun. 2020 Dec 11;11:6354. doi: 10.1038/s41467-020-20242-9 (PMC7733509; doi:10.1038/s41467-020-20242-9)
Supplement: Supplementary file 1 — Supplementary Information [file 41467_2020_20242_MOESM1_ESM.pdf]

Supplementary Information for

PRC2 and EHMT1 regulate H3K27me2 and H3K27me3 establishment across the zygote genome

(Meng et al.)

Includes:

Supplementary Figure. 1-7

Supplementary Table. 1-3

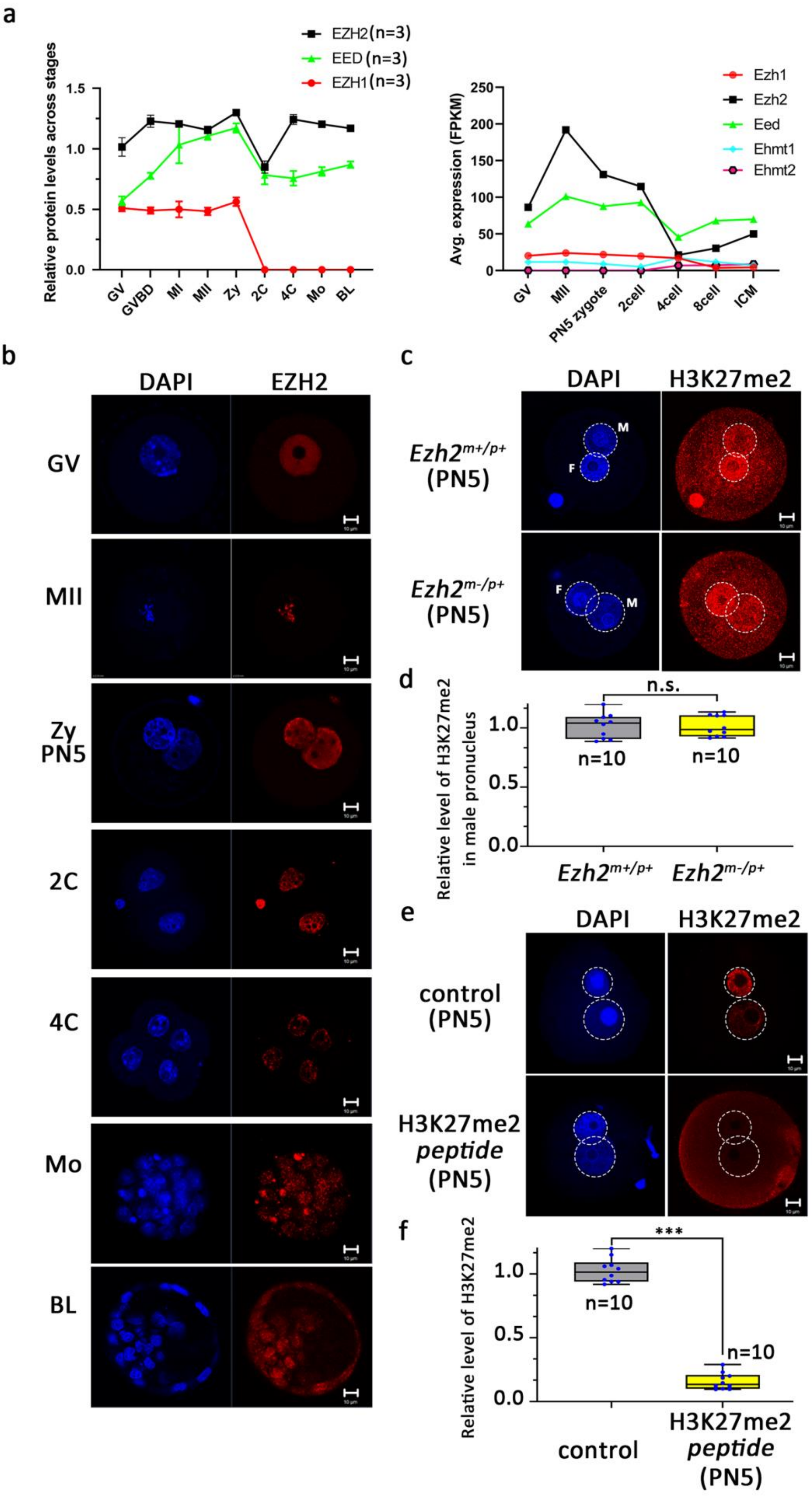
a
EZH2(n=3)
EED (n=3)
EZH1(n=3)
Relative protein levels across stages
GV GVBD MI MII Zy 2C 4C Mo BL
Ezh1
Ezh2
Eed
Ehmt1
Ehmt2
Avg. expression (FPKM)
GV MII PN5 zygote 2cell 4cell 8cell ICM
b
DAPI
EZH2
GV
MII
Zy PN5
2C
4C
Mo
BL
c
DAPI
H3K27me2
Ezh2m+/p+ (PN5)
Ezh2m-/p+ (PN5)
M
F
d
n.s.
Relative level of H3K27me2 in male pronucleus
n=10
n=10
Ezh2m+/p+
Ezh2m-/p+
e
DAPI
H3K27me2
control (PN5)
H3K27me2 peptide (PN5)
f
***
Relative level of H3K27me2
n=10
n=10
control
H3K27me2 peptide (PN5)

Supplementary figure 1. The expression pattern of several genes and H3K27me2 antibody specificity. (a) Left: Quantitated the levels of related proteins. n=3 biologically independent experiments. Error bars, mean±S.E.M.; Right: Average expression of genes during oocyte maturation and early embryonic development Analysis is based on RNA-seq data from Zhang et al.[1] (b) Immunostaining for EZH2 during oocyte maturation and early embryonic development. Zy PN5: PN5 stage zygote. 2C: 2-cell stage. 4C: 4-cell stage. Mo: Morula. BL: Blastocyst. Scale bar, 10 μm. (c) The H3K27me2 state of zygotes at 13h of IVF after maternal loss of EZH2. M indicates male pronucleus and F indicates female pronucleus in zygote. Scale bar, 10 μm. (d) Relative fluorescence intensity of H3K27me2 in PN5 stage Ezh2m+/p+ and Ezh2m-/p+ zygote pronucleus. Error bars, S.E.M. n.s. represents nonsignificant difference. $P > 0.7637$ by two-tailed Student's t tests. (e) The H3K27me2 state of zygotes at 13h of IVF after H3K27me2 peptide microinjection. Scale bar, 10 μm. (f) Relative fluorescence intensity of H3K27me2 in PN5 stage zygote pronucleus after H3K27me2 peptide microinjection. Error bars, S.E.M. ***$P < 4.66711E-15$ by two-tailed Student's t tests. The median line of box plot represents the median, and the top and bottom of the box represent the upper and lower quartile, respectively.

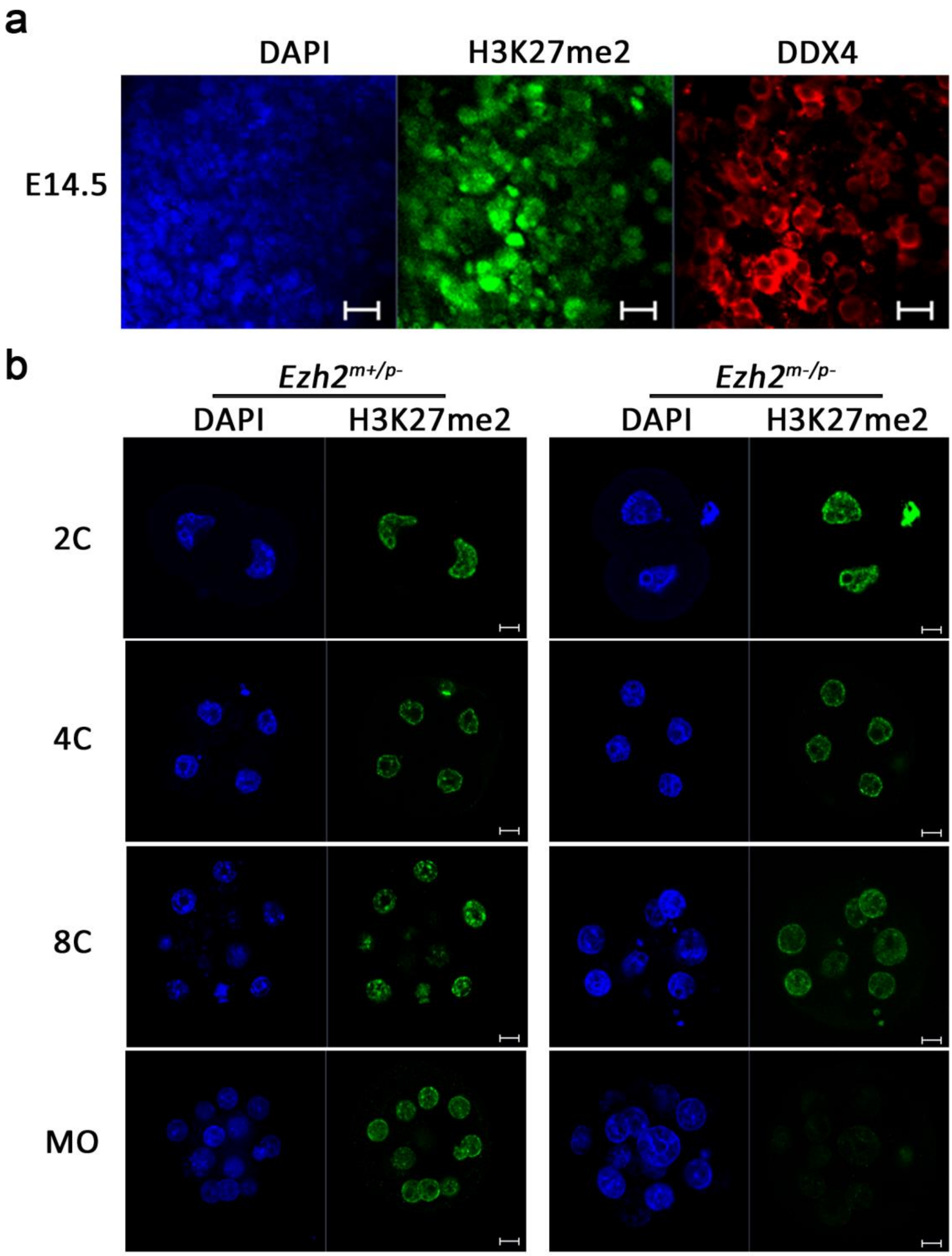

Supplementary figure 2. Immunostaining for H3K27me2 in E14.5 ovary germ cells and Ezh2m-/p- embryos. (a) Immunostaining for H3K27me2 in E14.5 ovary germ cells. Scale bar, 10 μm. (b) The H3K27me2 state in Ezh2m+/p- embryos and Ezh2m-/p- embryos from two-cell stage to morula stage. 2C: 2-cell stage. 4C: 4-cell stage. Mo: Morula. BL: Blastocyst. Scale bar, 10 μm.

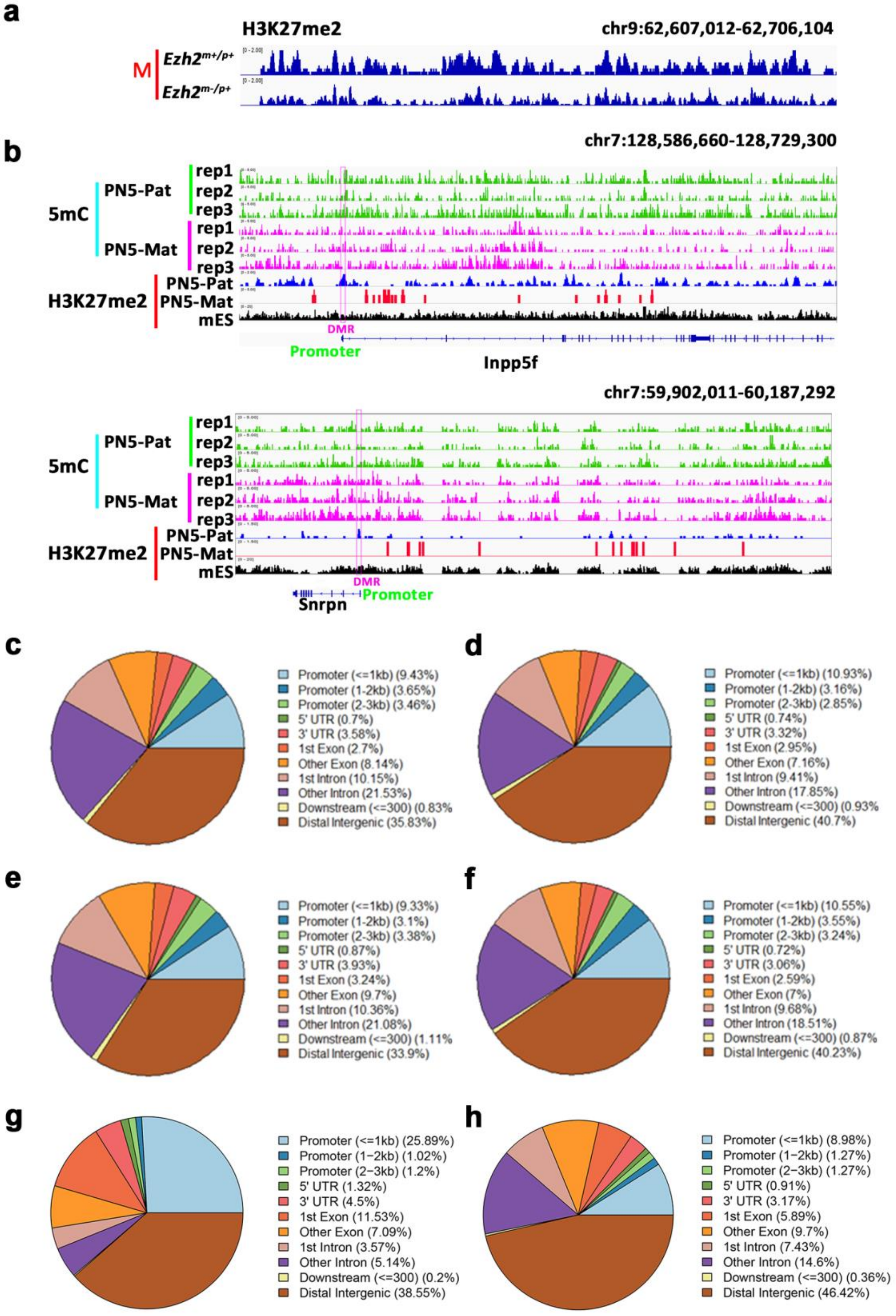
a
H3K27me2
chr9:62,607,012-62,706,104
M
Ezh2m+/p+
Ezh2m-/p+
b
chr7:128,586,660-128,729,300
5mC
PN5-Pat
PN5-Mat
rep1
rep2
rep3
H3K27me2
PN5-Pat
PN5-Mat
mES
DMR
Promoter
Inpp5f
chr7:59,902,011-60,187,292
5mC
PN5-Pat
PN5-Mat
rep1
rep2
rep3
H3K27me2
PN5-Pat
PN5-Mat
mES
DMR
Promoter
Snrpn
c
Promoter (<=1kb) (9.43%)
Promoter (1-2kb) (3.65%)
Promoter (2-3kb) (3.46%)
5' UTR (0.7%)
3' UTR (3.58%)
1st Exon (2.7%)
Other Exon (8.14%)
1st Intron (10.15%)
Other Intron (21.53%)
Downstream (<=300) (0.83%
Distal Intergenic (35.83%)
d
Promoter (<=1kb) (10.93%)
Promoter (1-2kb) (3.16%)
Promoter (2-3kb) (2.85%)
5' UTR (0.74%)
3' UTR (3.32%)
1st Exon (2.95%)
Other Exon (7.16%)
1st Intron (9.41%)
Other Intron (17.85%)
Downstream (<=300) (0.93%
Distal Intergenic (40.7%)
e
Promoter (<=1kb) (9.33%)
Promoter (1-2kb) (3.1%)
Promoter (2-3kb) (3.38%)
5' UTR (0.87%)
3' UTR (3.93%)
1st Exon (3.24%)
Other Exon (9.7%)
1st Intron (10.36%)
Other Intron (21.08%)
Downstream (<=300) (1.11%
Distal Intergenic (33.9%)
f
Promoter (<=1kb) (10.55%)
Promoter (1-2kb) (3.55%)
Promoter (2-3kb) (3.24%)
5' UTR (0.72%)
3' UTR (3.06%)
1st Exon (2.59%)
Other Exon (7%)
1st Intron (9.68%)
Other Intron (18.51%)
Downstream (<=300) (0.87%
Distal Intergenic (40.23%)
g
Promoter (<=1kb) (25.89%)
Promoter (1−2kb) (1.02%)
Promoter (2−3kb) (1.2%)
5' UTR (1.32%)
3' UTR (4.5%)
1st Exon (11.53%)
Other Exon (7.09%)
1st Intron (3.57%)
Other Intron (5.14%)
Downstream (<=300) (0.2%)
Distal Intergenic (38.55%)
h
Promoter (<=1kb) (8.98%)
Promoter (1−2kb) (1.27%)
Promoter (2−3kb) (1.27%)
5' UTR (0.91%)
3' UTR (3.17%)
1st Exon (5.89%)
Other Exon (9.7%)
1st Intron (7.43%)
Other Intron (14.6%)
Downstream (<=300) (0.36%)
Distal Intergenic (46.42%)

Supplementary figure 3. Genome-wide profiling of H3K27me2 in mouse zygotes. (a)The UCSC genome browser view showing allelic H3K27me2 enrichment in maternal genome of PN5 stage zygote. M, maternal. (b) The UCSC genome browser view showing H3K27me2 and 5mC signals in parental genome of PN5 stage zygote and mESC near imprinting genes Inpp5f and Snrpn. Pat, paternal. M, maternal. (c) The distribution of H3K27me2 in Ezh2m+/p+ maternal genomes. (d) The distribution of H3K27me2 in Ezh2m+/p+ paternal genomes. (e) The distribution of H3K27me2 in Ezh2m-/p+ maternal genomes. (f) The distribution of H3K27me2 in Ezh2m-/p+ paternal genomes. (g) The distribution of H3K27me3 in maternal genomes. (h) The distribution of H3K27me3 in paternal genomes. Shown is STAR ChIP-seq data from Zheng et al.[1]

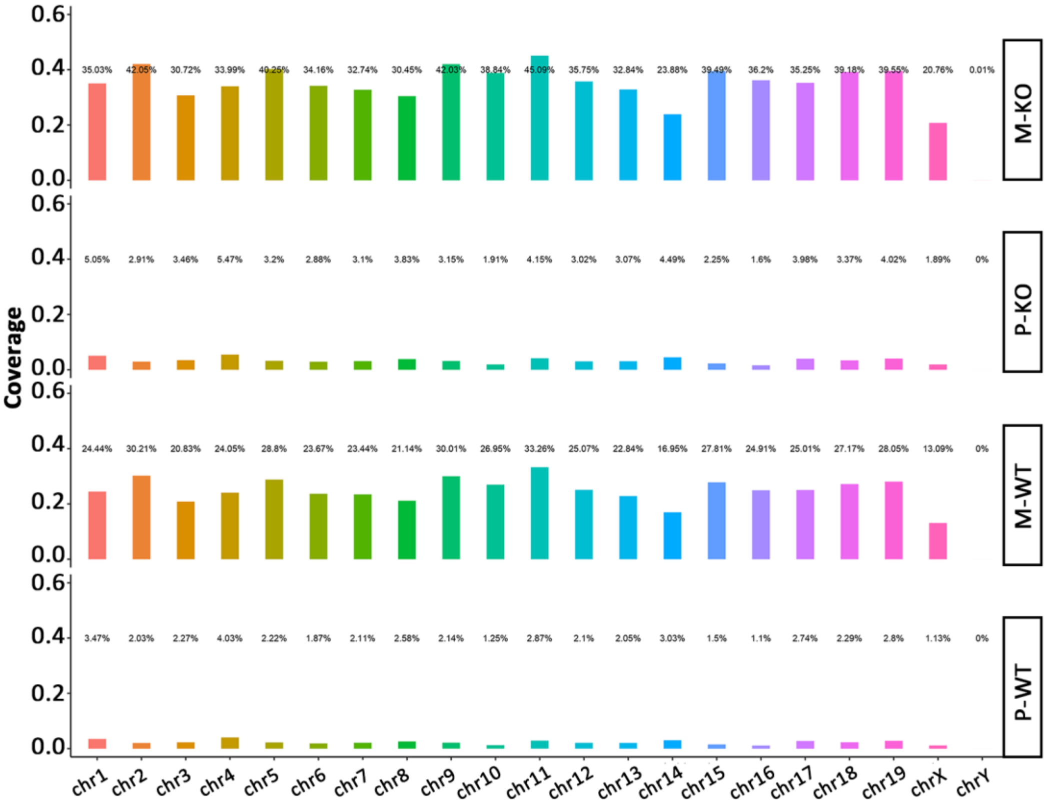

Supplementary figure 4. The level of de novo H3K27me2 in the paternal pronucleus and in the maternal pronucleus. The coverage of H3K27me2 in *Ezh2*$^{m-/p+}$ and *Ezh2*$^{m+/p+}$ parental genomes.

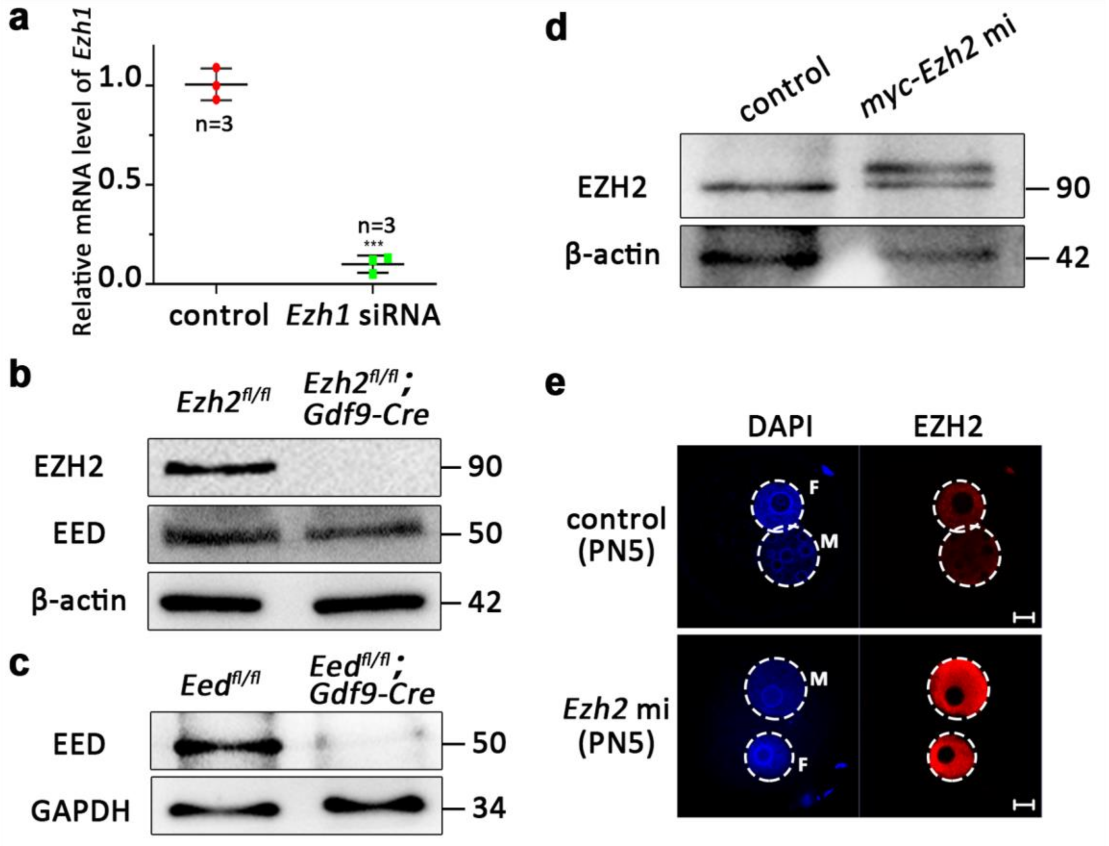

Supplementary figure 5. Analysis of knockout efficiency and overexpression of several genes. (a) Relative expression of ezh1 after ezh1 siRNA treatment in oocytes. Error bars, S.E.M. ***P < 6.82313E-05 by two-tailed Student's t tests. The median line of box plot represents the median, and the top and bottom of the box represent the upper and lower quartile, respectively. (b) The expression of EZH2 and EED in zygotes lack of maternal EZH2. (c) The expression of EED in GV oocytes after maternal deletion of EED. (d&e) Immunostaining for EZH2 in PN5 stage zygote after Ezh2 mRNA microinjection. M indicates male pronucleus and F indicates female pronucleus in zygote. Scale bar, 10 µm.

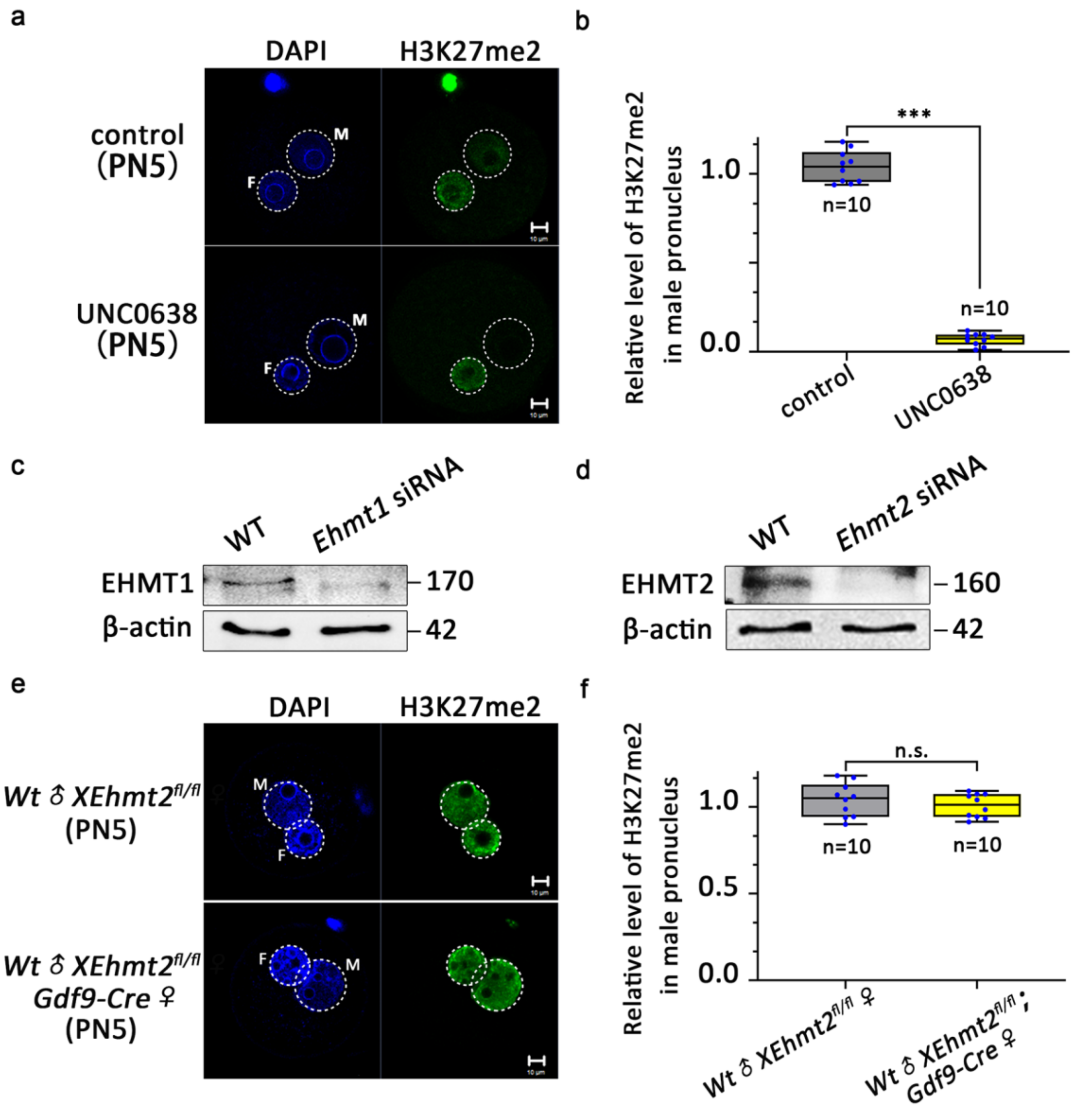

Supplementary figure 6. EHMT1 rather than EHMT2 is involved in de novo H3K27me2 in the paternal pronucleus of mouse zygote. (a) Immunostaining for H3K27me2 in PN5 stage zygote after UNC0638 treatment. M indicates male pronucleus and F indicates female pronucleus in zygote. Scale bar, 10 μm. (b) Relative fluorescence intensity of H3K27me2 in PN5 stage zygote pronucleus after UNC0638 treatment. Error bars, S.E.M. ***P < 2.59128E-17 by two-tailed Student's t tests. (c) The expression of EHMT1 in zygotes after Ehmt1 siRNA microinjection. (d) The expression of EHMT2 in zygotes after Ehmt2 siRNA microinjection. (e) Immunostaining for H3K27me2 in PN5 stage zygote after maternal loss of Ehmt2. M indicates male pronucleus and F indicates female pronucleus in zygote. Scale bar, 10 μm. (f) Relative fluorescence intensity of H3K27me2 in PN5 stage zygote pronucleus after maternal loss of Ehmt2. Error bars, S.E.M. n.s. represents nonsignificant difference. P >0.3860 by two-tailed Student's t tests. The median line of box plot represents the median, and the top and bottom of the box represent the upper and lower quartile, respectively.

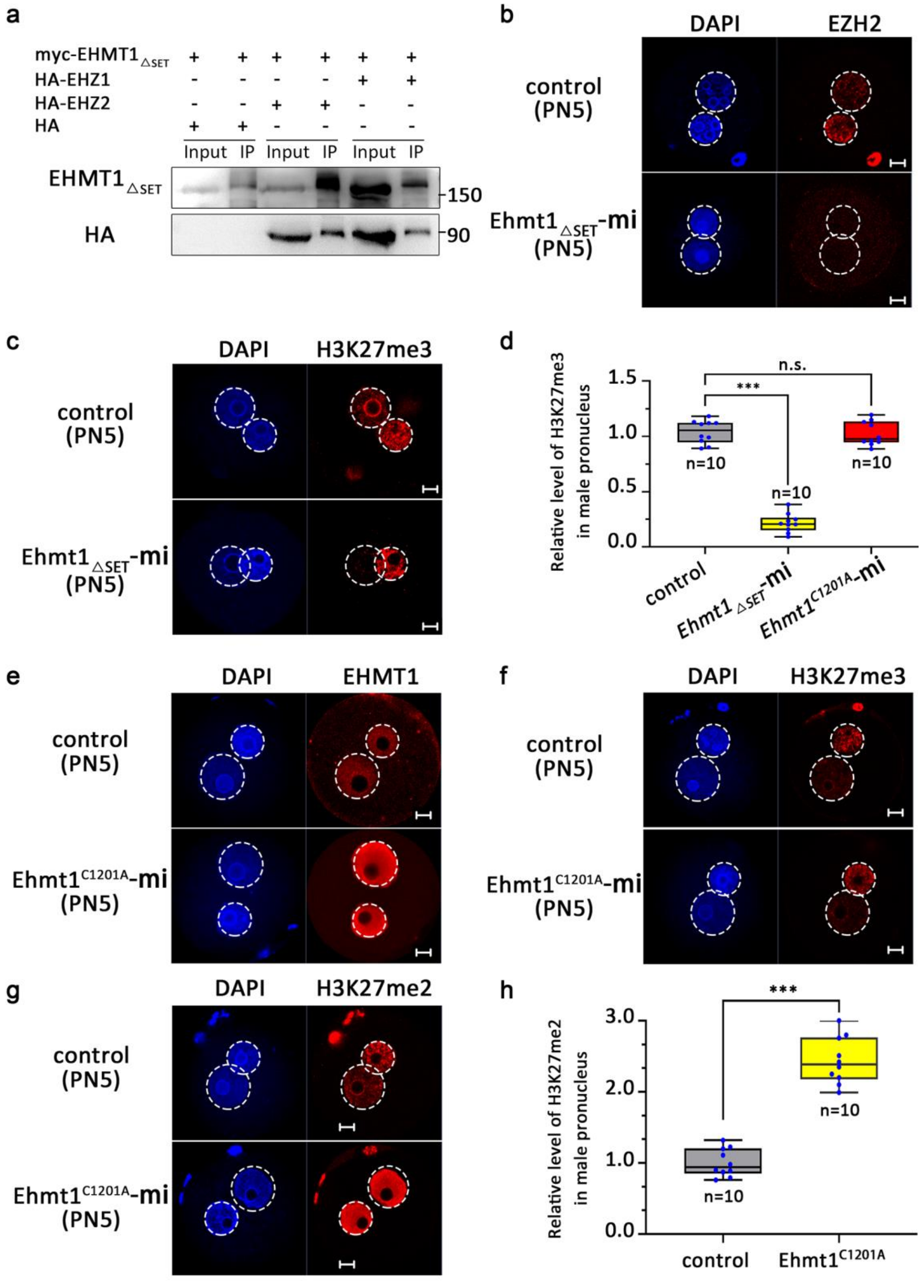
a
myc-EHMT1△SET
HA-EHZ1
HA-EHZ2
HA
Input
IP
EHMT1△SET
HA
150
90
b
DAPI
EZH2
control (PN5)
Ehmt1△SET-mi (PN5)
c
DAPI
H3K27me3
control (PN5)
Ehmt1△SET-mi (PN5)
d
Relative level of H3K27me3 in male pronucleus
n.s.
***
n=10
control
Ehmt1△SET-mi
Ehmt1C1201A-mi
e
DAPI
EHMT1
control (PN5)
Ehmt1C1201A-mi (PN5)
f
DAPI
H3K27me3
control (PN5)
Ehmt1C1201A-mi (PN5)
g
DAPI
H3K27me2
control (PN5)
Ehmt1C1201A-mi (PN5)
h
Relative level of H3K27me2 in male pronucleus
***
n=10
control
Ehmt1C1201A

Supplementary figure 7. EHMT1 function investigation. (a) Interaction of EHMT1ΔSET with EZH1 and EZH2 proteins were examined by Co-IP. EHMT1ΔSET -myc was co-transfected into HEK293T cells with EZH1-HA, EZH2-HA or HA-Vector, respectively. Anti-MYC was used to precipitate EHMT1ΔSET and associated proteins. Immunoblots were probed with EHMT1 and HA antibodies to detect the pull-down proteins (EZH1, EZH2) and EHMT1ΔSET, respectively. (b) Immunostaining for EZH2 in PN5 stage zygote after Ehmt1ΔSET mRNA microinjection. Scale bar, 10 μm. (c) Immunostaining for H3K27me3 in PN5 stage zygote after Ehmt1ΔSET mRNA microinjection. Scale bar, 10 μm. (d) Relative fluorescence intensity of H3K27me3 in male pronucleus of PN5 stage zygote after Ehmt1ΔSET (in C) and Ehmt1C1201A (in F) mRNA microinjection. Error bars, S.E.M. ***P < 1.6995E-13. n.s. represents nonsignificant difference. P >0.7442 by two-tailed Student's t tests. (e) Immunostaining for EHMT1 in PN5 stage zygote after Ehmt1C1201A mRNA microinjection. Scale bar, 10 μm. (f) Immunostaining for H3K27me3 in PN5 stage zygote after Ehmt1C1201A mRNA microinjection. Scale bar, 10 μm. (g) Immunostaining for H3K27me2 in PN5 stage zygote after Ehmt1C1201A mRNA microinjection. Scale bar, 10 μm. (h) Relative fluorescence intensity of H3K27me2 in male pronucleus of PN5 stage zygote after Ehmt1C1201A (in G) mRNA microinjection. Error bars, S.E.M. ***P < 6.07641E-10 by two-tailed Student's t tests. The median line of box plot represents the median, and the top and bottom of the box represent the upper and lower quartile, respectively.

Supplementary Table 1. Breeding assay of *Eed*$^{fl/fl}$ and *Eed*$^{fl/fl}$*;Gdf9-Cre* female mice （n=5）

Genotype of female	pups per litter (mean±SEM)
Eed$^{fl/fl}$	8.000 ± 0.3568
Eed$^{fl/fl}$*; Gdf9-Cre*	3.182 ± 0.2960

Supplementary Table 2. Primers used for PCR

primer	sequence (5'-3')
Ezh2 flox forward	CATGTGCAGCTTTCTGTTCA
Ezh2 flox reverse	CACAGCCTTTCTGCTCACTG
Eed flox forward	TGGAGAGGTGGCTGAGAGTT
Eed flox reverse	TCCTTCTCTCCCCCAAATTC
Ezh1-qF	AATATGGGAGCAAAGGCTCTGTATGTG
Ezh1-qR	CACGAAGTTTCTTCCACTCTTCATTGAG
Gdf9-Cre-F	AGGCATGCTTGAGGTCTGAT
Gdf9-Cre-R	CACAGTCAGCAGGTTGGAGA
Ehmt2 flox forward	GCTGAGGGAACAGACCCATAACTTT
Ehmt2 flox reverse	GCATGTCATCACTCATGCGGAAATG
Ehmt1-qF	CAGATGGAGAAACAAATGGGTCT
Ehmt1-qR	TTTGCTTCCCCACTTCTGTGT
Ehmt2-qF	TGGGAACTTGGAAATGGTCAG
Ehmt2-qR	GGGTCAGCAGCATACGAATCAC

Supplementary Table 3. siRNA sequence for RNAi

siRNA	sequence (5’-3’)
Ezh1 siRNA#1 sense	GGACUGUAAGGUGGUGUCUUGCAAA
Ezh1 siRNA#1 antisense	UUUGCAAGACACCACCUUACAGUCC
Ezh1 siRNA#2sense	CCAUUGCGUCCAUGUUUCCUGAGAA
Ezh1 siRNA#2 antisense	UUCUCAGGAAACAUGGACGCAAUGG
Ehmt1 siRNA sense	UGGAGCUGAGCAAGCGGCUGCAUUU
Ehmt1 siRNA antisense	AAAUGCAGCCGCUUGCUCAGCUCCA
Ehmt2 siRNA sense	ACAAGCACAUCGAUGUGAUUCGUAU
Ehmt2 siRNA antisense	AUACGAAUCACAUCGAUGUGCUUGU

Reference：

1. Zheng, H. *et al.* Resetting Epigenetic Memory by Reprogramming of Histone Modifications in Mammals. *Mol Cell* **63**, 1066-79 (2016).
