# Supplementary material for: PRC2 and EHMT1 regulate H3K27me2 and H3K27me3 establishment across the zygote genome
Source: Nat Commun. 2020 Dec 11;11:6354. doi: 10.1038/s41467-020-20242-9 (PMC7733509; doi:10.1038/s41467-020-20242-9)
Supplement: Supplementary file 3 — Reporting Summary [file 41467_2020_20242_MOESM3_ESM.pdf]

nature research

Corresponding author(s): Qing-Yuan Sun

Last updated by author(s): Sep 21, 2020

Reporting Summary

Nature Research wishes to improve the reproducibility of the work that we publish. This form provides structure for consistency and transparency in reporting. For further information on Nature Research policies, see our Editorial Policies and the Editorial Policy Checklist.

Statistics

For all statistical analyses, confirm that the following items are present in the figure legend, table legend, main text, or Methods section.

n/a	Confirmed	
☐	☒	The exact sample size (*n*) for each experimental group/condition, given as a discrete number and unit of measurement
☐	☒	A statement on whether measurements were taken from distinct samples or whether the same sample was measured repeatedly
☐	☒	The statistical test(s) used AND whether they are one- or two-sided<br>*Only common tests should be described solely by name; describe more complex techniques in the Methods section.*
☐	☒	A description of all covariates tested
☒	☐	A description of any assumptions or corrections, such as tests of normality and adjustment for multiple comparisons
☐	☒	A full description of the statistical parameters including central tendency (e.g. means) or other basic estimates (e.g. regression coefficient) AND variation (e.g. standard deviation) or associated estimates of uncertainty (e.g. confidence intervals)
☐	☒	For null hypothesis testing, the test statistic (e.g. *F*, *t*, *r*) with confidence intervals, effect sizes, degrees of freedom and *P* value noted<br>*Give P values as exact values whenever suitable.*
☒	☐	For Bayesian analysis, information on the choice of priors and Markov chain Monte Carlo settings
☒	☐	For hierarchical and complex designs, identification of the appropriate level for tests and full reporting of outcomes
☒	☐	Estimates of effect sizes (e.g. Cohen's *d*, Pearson's *r*), indicating how they were calculated

Our web collection on statistics for biologists contains articles on many of the points above.

Software and code

Policy information about availability of computer code

Data collection	ULI-NChIP library was generated using the KAPA Hyper Prep Kit (KK8504) according to the manufacturer's protocol. Paired-end 150-bp sequencing was performed on a HiSeq2500.No software was used in this analysis.
Data analysis	High quality unique mapping was performed by Samtools (version 1.6) with MAPQ more than 30 and self-coded Perl scripts. The duplication reads were removed by Picard (version 2.0.1) to obtain the non-redundant reads. SNP split was utilized to split the maternal and paternal reads alignment with the allele-specific C57BL6 and PWK mm10 genome (buit-in SNPsplit). SICER was utilized to call peaks (windows 200, Gap 600, q value 1e-5) with SICER.sh module and the differential peaks were found with SICER-df.sh module32. The peaks filtered by length more than 1000bp and fold change more than 5 were annotated by Chip Seeker for gene category analysis and Cluster profiler for gene function annotation such as KEGG and GO analysis. The depth and coverage of ChIP sequencing data were calculated by Bed tools with the 5kb windows and selfcoded python scripts, respectively, which were visualized by ggpubr and ggplot packages in R with the Wilcoxon rank test.

For manuscripts utilizing custom algorithms or software that are central to the research but not yet described in published literature, software must be made available to editors and reviewers. We strongly encourage code deposition in a community repository (e.g. GitHub). See the Nature Research guidelines for submitting code & software for further information.

Data

Policy information about availability of data

All manuscripts must include a data availability statement. This statement should provide the following information, where applicable:

- Accession codes, unique identifiers, or web links for publicly available datasets
- A list of figures that have associated raw data
- A description of any restrictions on data availability

GEO accession GSE134592 Go to https://www.ncbi.nlm.nih.gov/geo/query/acc.cgi?acc=GSE134592 Enter token clmlqyckddwrfud into the box

Figure 1K&L Figure S3 Figure S4
The ULI-NChIP data are released on July, 2020.
The processing codes are following the documentations of each software, and all other relevant materials are available on request.

Field-specific reporting

Please select the one below that is the best fit for your research. If you are not sure, read the appropriate sections before making your selection.

- [x] Life sciences
- [ ] Behavioural & social sciences
- [ ] Ecological, evolutionary & environmental sciences

For a reference copy of the document with all sections, see nature.com/documents/nr-reporting-summary-flat.pdf

Life sciences study design

All studies must disclose on these points even when the disclosure is negative.

Sample size
For ULI-NChIP library, 300 zygotes were collected for each group. Determined by counting under the microscope

Data exclusions
The raw reads were processed by Trimmomatic (version 0.38) to cut adapters and to trim low quality reads with the minimal length 90bp and minimal quality of bases 20. Clean reads were mapped to the mouse mm10 genome by Bowtie2 with parameters as described previously and the default parameters in zygotes and MII ChIP sequencing data, respectively.

Replication
The raw reads were processed by Trimmomatic (version 0.38) to cut adapters and to trim low quality reads with the minimal length 90bp and minimal quality of bases 20. Clean reads were mapped to the mouse mm10 genome by Bowtie2 with parameters as described previously and the default parameters in zygotes and MII ChIP sequencing data, respectively. For experiments other that ULI-NChIP, all experimental results have been conducted at least three independent experiments using different batches of samples

Randomization
Repeat three times at least for experiment. Samples for each experiment are collected randomly and independently.

Blinding
No, the conditional knockout mice have been genotyped by PCR. For experiments other than those involving conditional knockout mice, the relevant experimental results were verified and confirmed by independent experiments by X.S. Ma, T.G. Meng and Q. Zhou.

Reporting for specific materials, systems and methods

We require information from authors about some types of materials, experimental systems and methods used in many studies. Here, indicate whether each material, system or method listed is relevant to your study. If you are not sure if a list item applies to your research, read the appropriate section before selecting a response.

Materials & experimental systems

n/a	Involved in the study
[ ]	[x] Antibodies
[x]	[ ] Eukaryotic cell lines
[x]	[ ] Palaeontology and archaeology
[ ]	[x] Animals and other organisms
[x]	[ ] Human research participants
[x]	[ ] Clinical data
[x]	[ ] Dual use research of concern

Methods

n/a	Involved in the study
[ ]	[x] ChIP-seq
[x]	[ ] Flow cytometry
[x]	[ ] MRI-based neuroimaging

Antibodies

Antibodies used
Mouse anti-Ezh2 antibody (BD Bioscience; 1:200), rabbit anti-Ezh1 antibody (Abcam; ab176115), rabbit anti-EHMT1 antibody (Abcam; ab41969;1:100), rabbit anti-H3K27me2 antibody (Cell Signaling, 9728S;1:200;lot NO:0011), rabbit anti-H3K27me3 antibody (Cell Signaling, 9733S;1:200,lot NO:0008). anti-H3K27me2 (for ULI-NChIP, Actif Motif;61435). Accordingly, the following secondary antibodies were used: Goat anti-mouse IgG(H+L) Alexa Fluor 488 (Invitrogen;A-11001; 1:1000); goat anti-rabbit IgG(H+L) Alexa Fluor 488 (Invitrogen; A-11008; 1:1000); Goat anti-mouse IgG(H+L) Alexa Fluor 594 (Invitrogen;R37121;1:1000); goat anti-rabbit IgG(H+L) Alexa Fluor 594 (Invitrogen; A-11012; 1:1000)
We have also stated the dilutions used for the antibodies.

Validation
Each primary antibody was confirmed, each experiment was repeated at least three times.
All the antibodies are from commercial source and have been validated by the vendors and their validation data are available on the manufacturers' website (BD Bioscience, Abcam, Cell Signaling, Actif Motif, Invitrogen).

Animals and other organisms

Policy information about studies involving animals; ARRIVE guidelines recommended for reporting animal research

Laboratory animals	Conditional Ezh2Flox/Flox. Ezh2flox/flox;Gdf9-Cre female mice are used in our study. These mice are maintained on a C57BL/6J genetic background at the age of 6 week at least. The mating strategy for maternal knockout of G9a and EED is the same as that of EZH2.<br>6 weeks old ICR mice, also called CD-1 mice, were used in the experiment, which were all purchased fromSPF (Beijing) Biotechnology.Co.,Ltd.<br>We also provide information on housing conditions for the mice, describing ambient temperature and humidity in the manuscript
Wild animals	No wild animals were used in the study
Field-collected samples	No field collected samples were used in the study.
Ethics oversight	Mice have free access to water and food supplies, as approved by the ethics committee for animal care of the Institute of Zoology, Chinese Academy of Sciences.

Note that full information on the approval of the study protocol must also be provided in the manuscript.

ChIP-seq

Data deposition

☒ Confirm that both raw and final processed data have been deposited in a public database such as GEO.

☒ Confirm that you have deposited or provided access to graph files (e.g. BED files) for the called peaks.

Data access links<br>*May remain private before publication.*	https://www.ncbi.nlm.nih.gov/geo/query/acc.cgi?acc=GSE134592 Enter token clmlqyckddwrfud into the box
Files in database submission	EZH2m+p+ chip.genome1-W200-G600-FDR1e-5-islandfiltered-normalized<br>EZH2m+p+ chip.genome2-W200-G600-FDR1e-5-islandfiltered-normalized<br>EZH2m-p+ chip.genome1-W200-G600-FDR1e-5-islandfiltered-normalized<br>EZH2m-p+ chip.genome2-W200-G600-FDR1e-5-islandfiltered-normalized<br>depth<br>H3K27me2_ZygoteEZH2_Maternal<br>H3K27me2_ZygoteEZH2_Paternal<br>H3K27me2_ZygoteWT_Maternal<br>H3K27me2_ZygoteWT_Paternal<br>EZH2m+p+_genome1_broad_treat_pileup<br>EZH2m+p+_genome2_broad_treat_pileup<br>EZH2m-p+_genome1_broad_treat_pileup<br>EZH2m-p+_genome2_broad_treat_pileup<br>EZH2m+p+ zygote H3K27me2_ChIPSeq_1<br>EZH2m+p+ zygote H3K27me2_ChIPSeq input_1<br>EZH2m-p+ zygote H3K27me2_ChIPSeq_1<br>EZH2m-p+ zygote H3K27me2_ChIPSeq input_1<br>EZH2m+p+ zygote H3K27me2_ChIPSeq_2<br>EZH2m+p+ zygote H3K27me2_ChIPSeq input_2<br>EZH2m-p+ zygote H3K27me2_ChIPSeq_2<br>EZH2m-p+ zygote H3K27me2_ChIPSeq input_2
Genome browser session<br>(e.g. UCSC)	https://www.ncbi.nlm.nih.gov/geo/query/acc.cgi?acc=GSE134592

Methodology

Replicates	For ULI-NChIP library, the conditional EZH2 mice is not enough, so only one repeat for each group was collected.
Sequencing depth	We used mm10 total genome length to evaluate the depth of sequencing. All reads length are 150bp, paired-end.<br>Sample name, sequencing depth, total number of clean reads and uniquely mapped reads are listed below.<br>EZH2m+p+ chip: 2.96, 26934491,35449394.<br>EZH2m+p+ input: 1.93, 17530634, 13752250.<br>EZH2m-p+ chip: 1.93, 17541699, 23049654.<br>EZH2m-p+ input: 1.09, 9927983, 9058694.
Antibodies	anti-H3K27me2 (for ULI-NChIP, Actif Motif; 61435).
Peak calling parameters	Command line:<br>SICER.sh [InputDirectory] [Chip.bed] [Input.bed] [OutputDir] mm10 1 200 150 0.74 600 1e-5

Parameters used for peak calling: GAP 600, Windows 200, FDR 1e-5.

Data quality

Keep FDR less than 1e-5, peaks more than 1kb and fold more than 5. Peak number are listed below.
EZH2m+p+ genome1 5744
EZH2m+p+ genome2 17863
EZH2m-p+ genome1 9606
EZH2m-p+ genome2 22823

Software

The raw reads were processed by Trimmomatic (version 0.38) to cut adapters and to trim low quality reads with the minimal length 90bp and minimal quality of bases 20. Clean reads were mapped to the mouse mm10 genome by Bowtie2 with parameters as described previously and the default parameters in zygote ChIP sequencing data, respectively. High quality unique mapping was performed by Samtools with MAPQ more than 30 and self-coded Perl scripts. The duplication reads were removed by Picard to obtain the non-redundant reads. SNP split was utilized to split the maternal and paternal reads alignment with the allele-specific C57BL6 and PWK mm10 genome. SICER was utilized to call peaks (windows 200, Gap 600, q value 1e-5) with SICER.sh module and the differential peaks were found with SICER-df.sh module29. The peaks filtered by length more than 1000bp and fold change more than 5 were annotated by Chip Seeker for gene category analysis. The depth and coverage of ChIP sequencing data were calculated by Bed tools with the 5kb windows and self-coded python scripts, respectively, which were visualized by ggpubr and ggplot packages in R with the Wilcoxon rank test.
