# Supplementary figures and images for: PRC2 and EHMT1 regulate H3K27me2 and H3K27me3 establishment across the zygote genome
Source: Nat Commun. 2020 Dec 11;11:6354. doi: 10.1038/s41467-020-20242-9 (PMC7733509; doi:10.1038/s41467-020-20242-9)

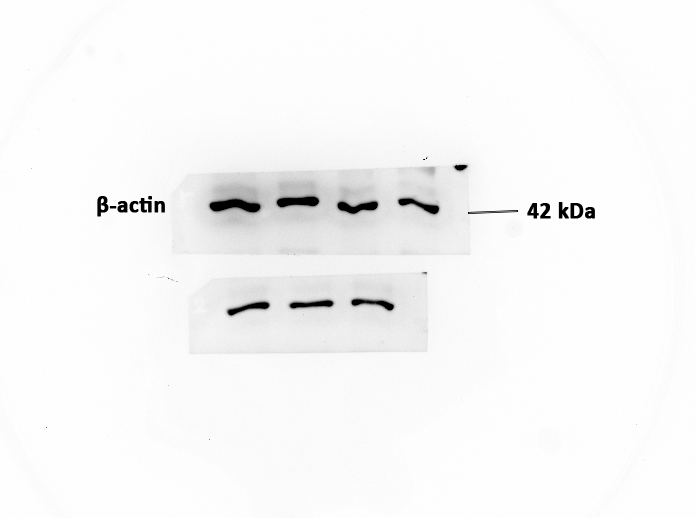

Supplement: Supplementary file 4 — Source Data [file 41467_2020_20242_MOESM4_ESM.zip › source data/Figure 1/Figure 1A actin during oocyte maturation.tif]

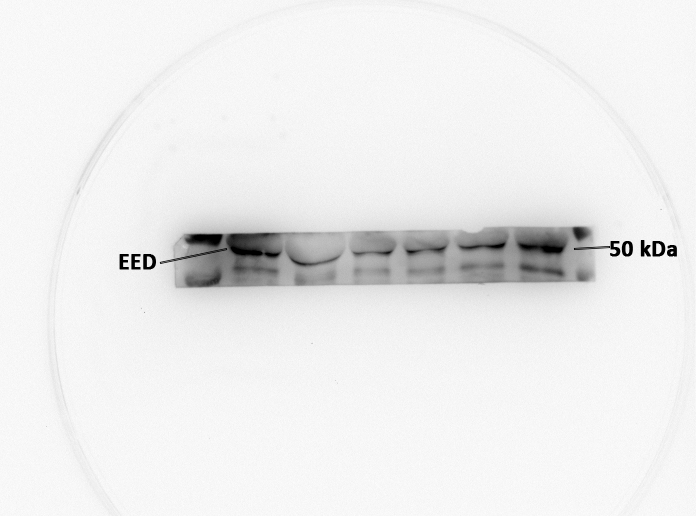

Supplement: Supplementary file 4 — Source Data [file 41467_2020_20242_MOESM4_ESM.zip › source data/Figure 1/Figure 1A EED during early embryo development.tif]

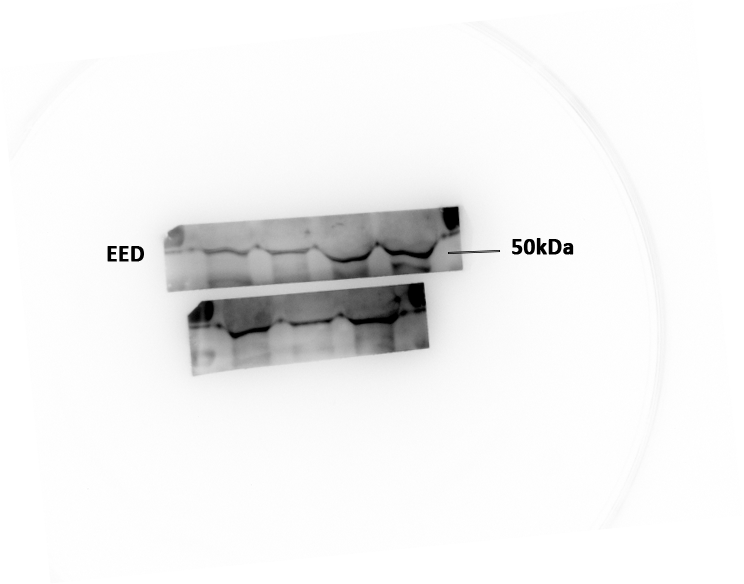

Supplement: Supplementary file 4 — Source Data [file 41467_2020_20242_MOESM4_ESM.zip › source data/Figure 1/Figure 1A EED during oocyte maturation.tif]

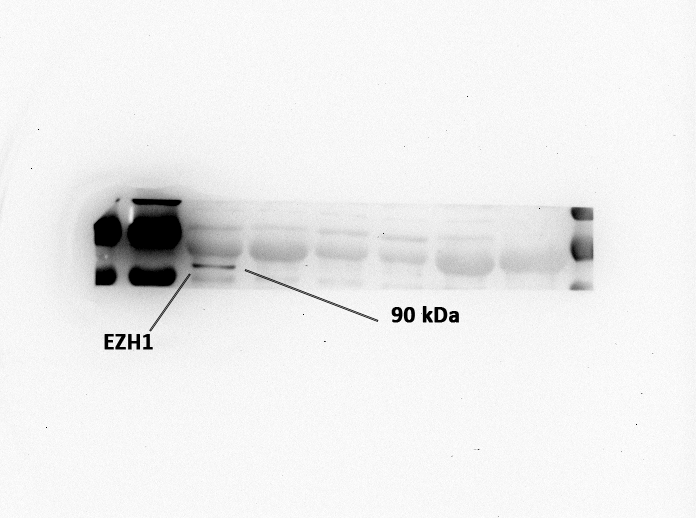

Supplement: Supplementary file 4 — Source Data [file 41467_2020_20242_MOESM4_ESM.zip › source data/Figure 1/Figure 1A EZH1 during early embryo development.tif]

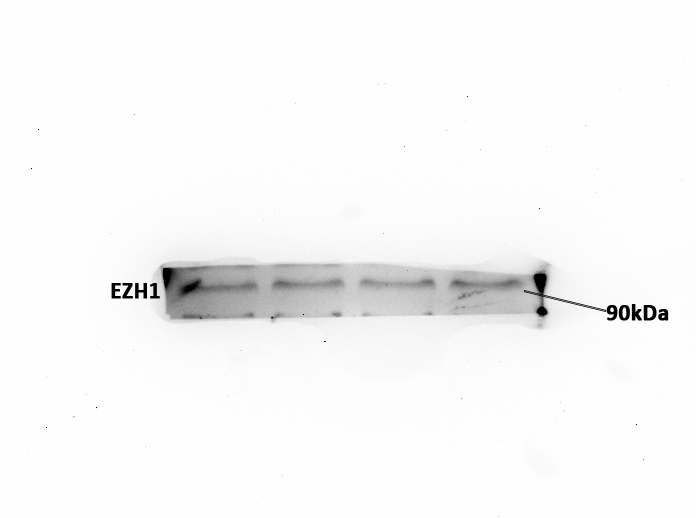

Supplement: Supplementary file 4 — Source Data [file 41467_2020_20242_MOESM4_ESM.zip › source data/Figure 1/Figure 1A EZH1 during oocyte maturation.tif]

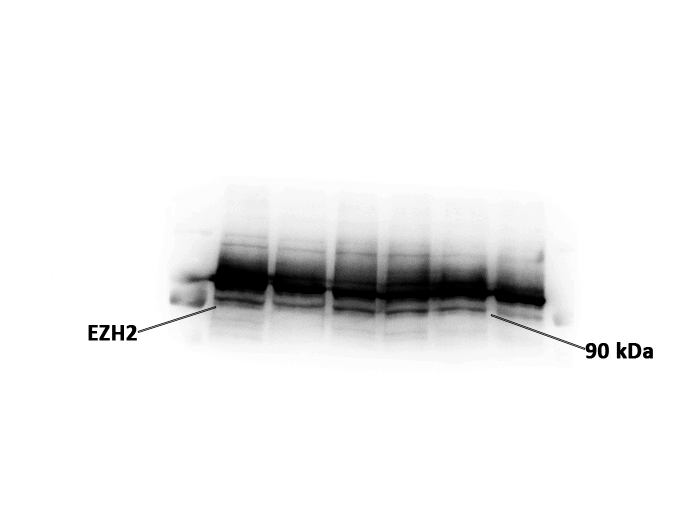

Supplement: Supplementary file 4 — Source Data [file 41467_2020_20242_MOESM4_ESM.zip › source data/Figure 1/Figure 1A EZH2 during early embryo development.tif]

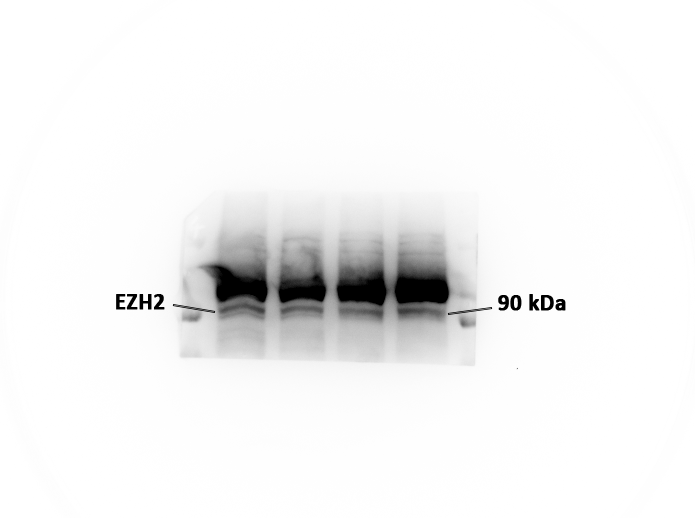

Supplement: Supplementary file 4 — Source Data [file 41467_2020_20242_MOESM4_ESM.zip › source data/Figure 1/Figure 1A EZH2 during oocyte maturation.tif]

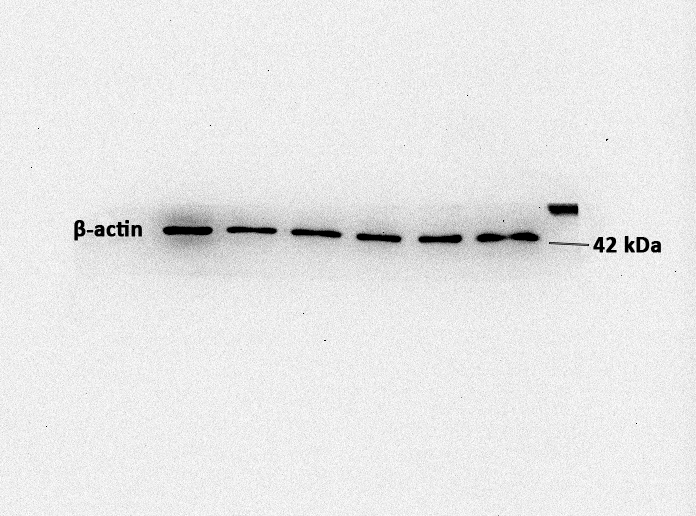

Supplement: Supplementary file 4 — Source Data [file 41467_2020_20242_MOESM4_ESM.zip › source data/Figure 1/Figure 1A β-actin during early embryo development.tif]

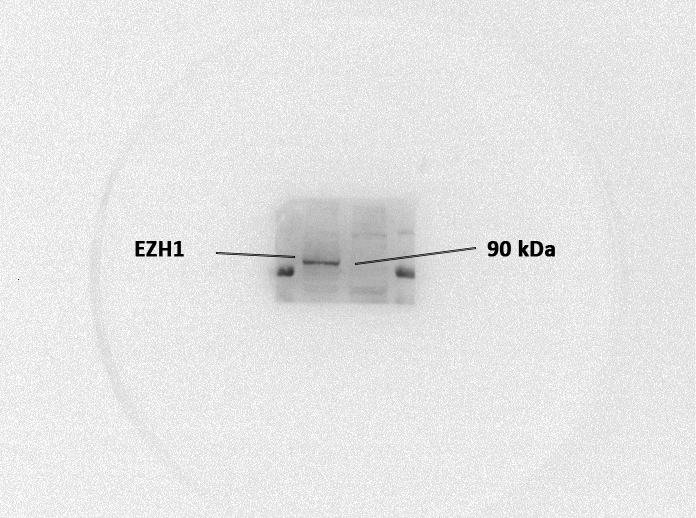

Supplement: Supplementary file 4 — Source Data [file 41467_2020_20242_MOESM4_ESM.zip › source data/Figure 2/Figure 2A EZH1.tif]

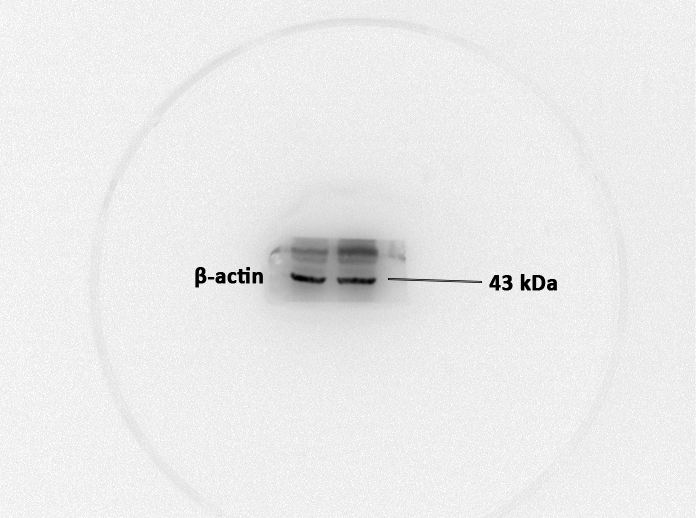

Supplement: Supplementary file 4 — Source Data [file 41467_2020_20242_MOESM4_ESM.zip › source data/Figure 2/Figure 2A β-actin.tif]

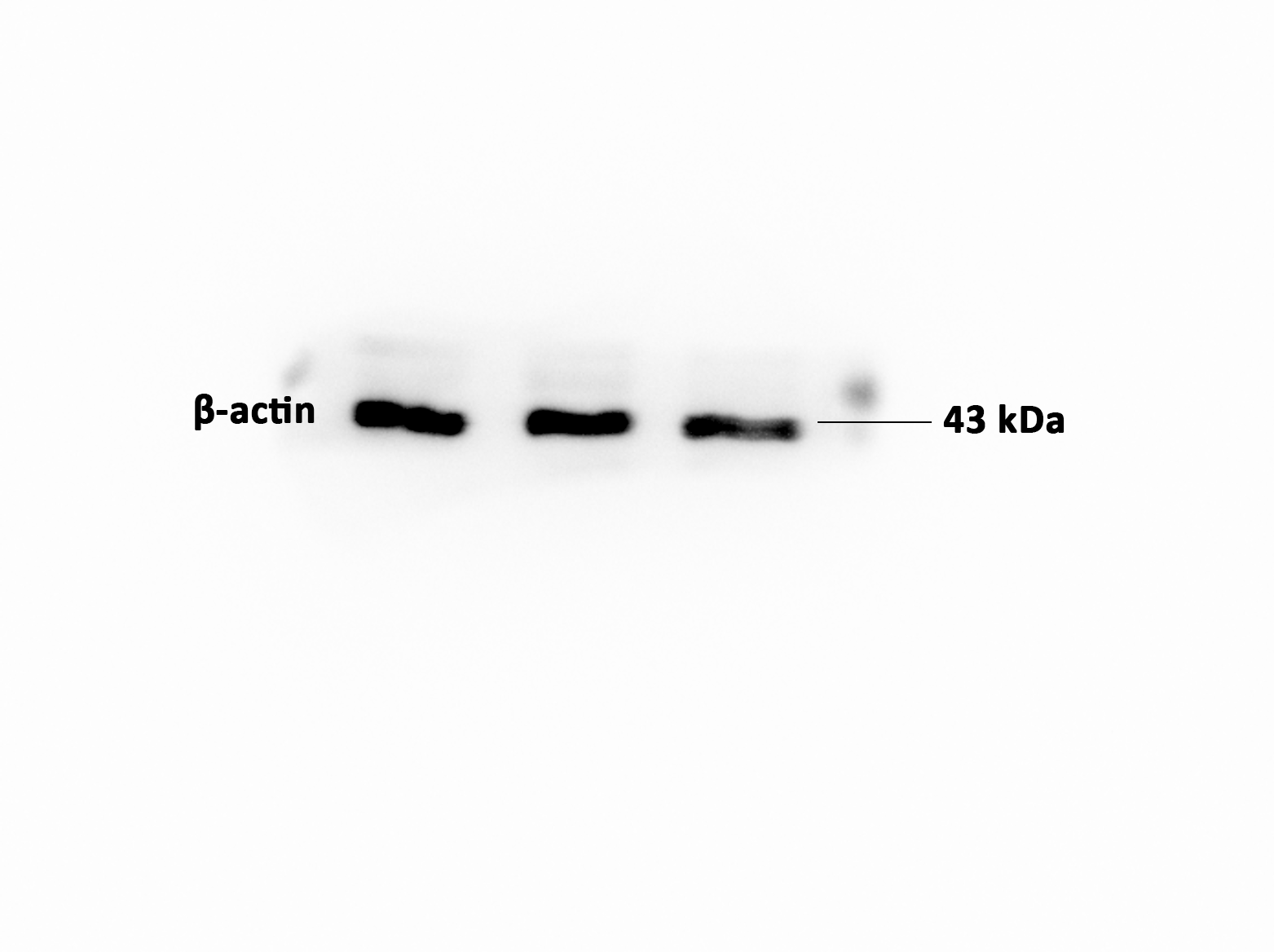

Supplement: Supplementary file 4 — Source Data [file 41467_2020_20242_MOESM4_ESM.zip › source data/Figure 3/Figure 3G actin.tif]

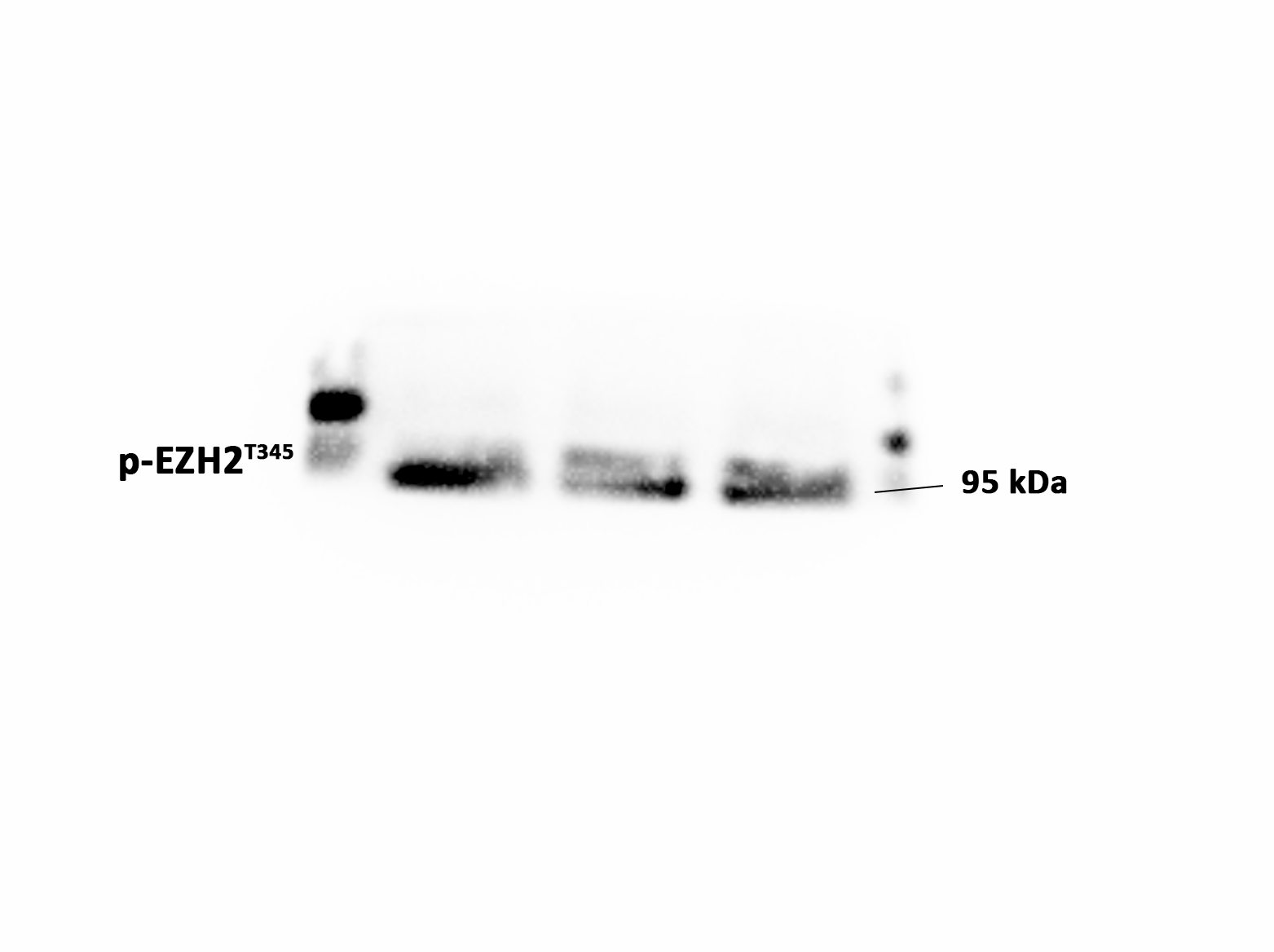

Supplement: Supplementary file 4 — Source Data [file 41467_2020_20242_MOESM4_ESM.zip › source data/Figure 3/Figure 3G p-EZH2.tif]

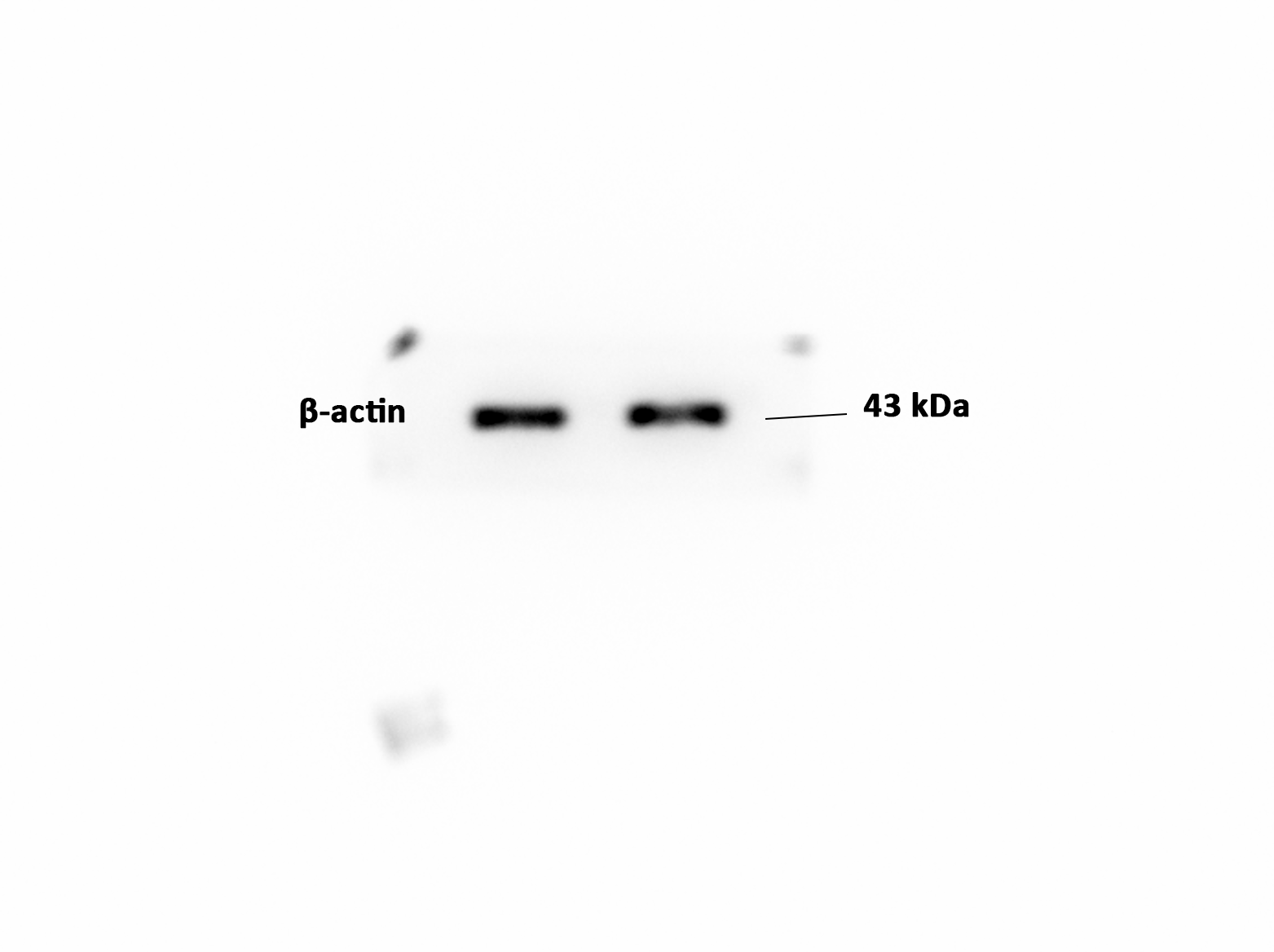

Supplement: Supplementary file 4 — Source Data [file 41467_2020_20242_MOESM4_ESM.zip › source data/Figure 5/Figure 5B down-panel β-actin.tif]

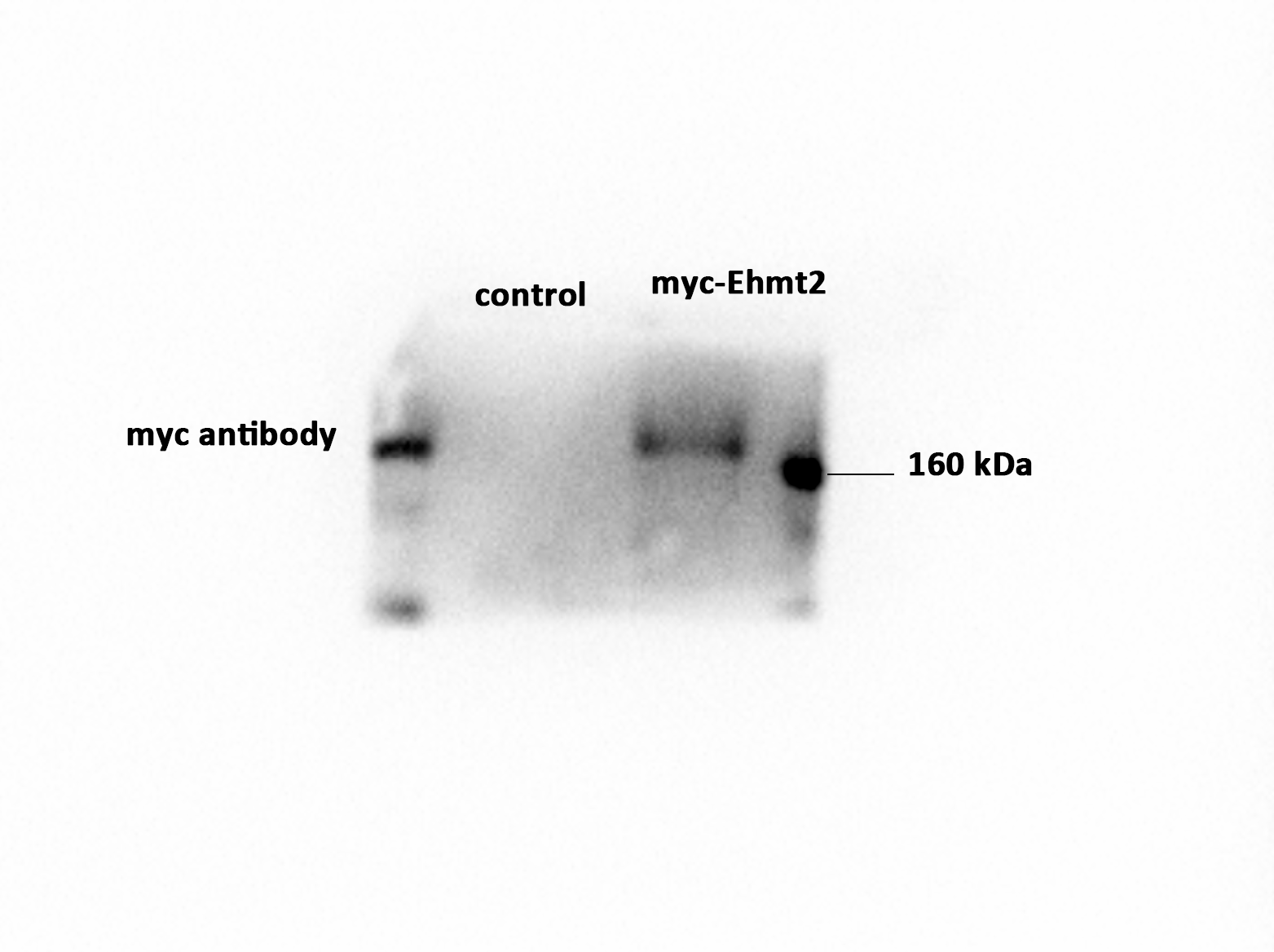

Supplement: Supplementary file 4 — Source Data [file 41467_2020_20242_MOESM4_ESM.zip › source data/Figure 5/Figure 5B down-panel-myc.tif]

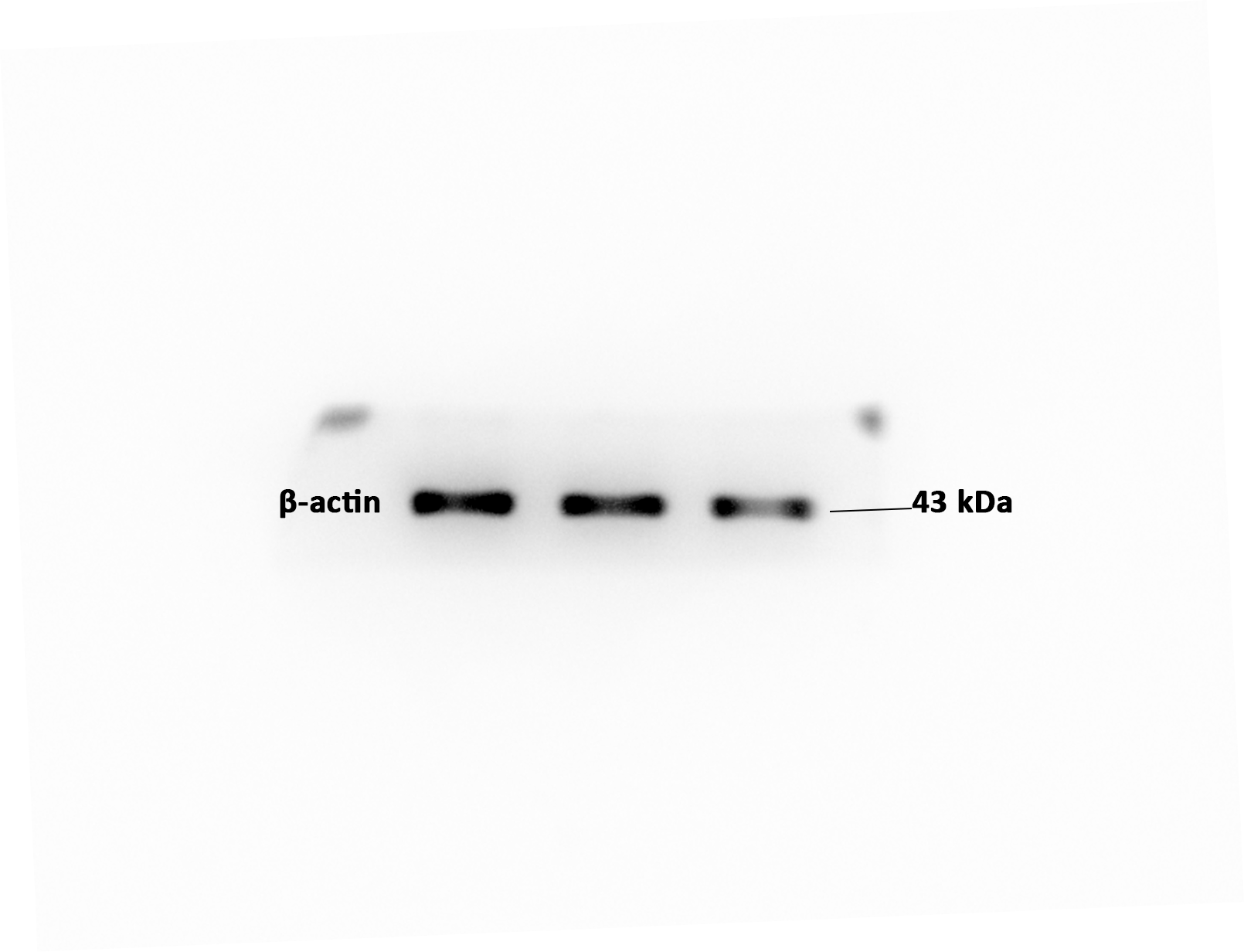

Supplement: Supplementary file 4 — Source Data [file 41467_2020_20242_MOESM4_ESM.zip › source data/Figure 5/Figure 5B up-panel actin.tif]

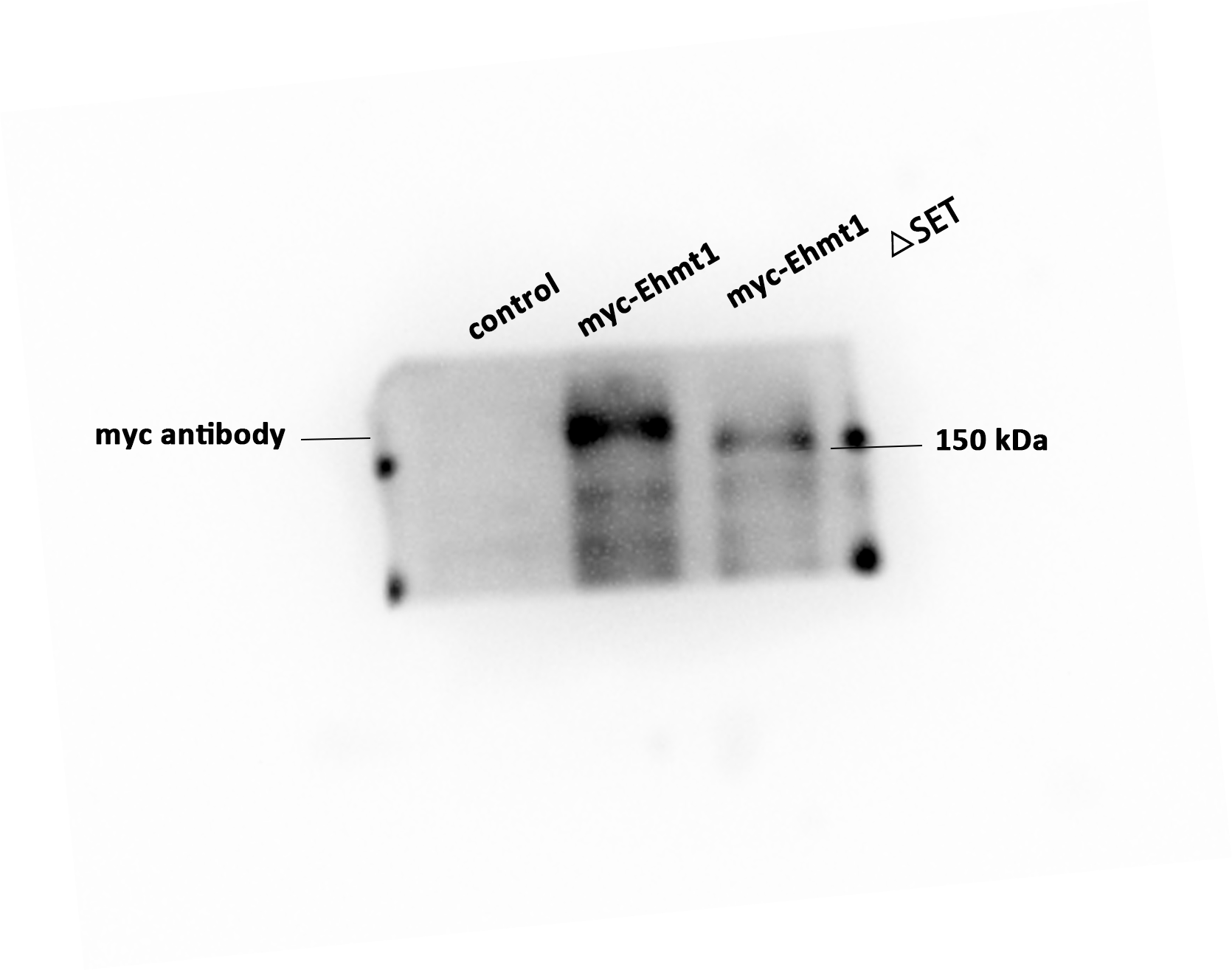

Supplement: Supplementary file 4 — Source Data [file 41467_2020_20242_MOESM4_ESM.zip › source data/Figure 5/Figure 5B up-panel myc.tif]

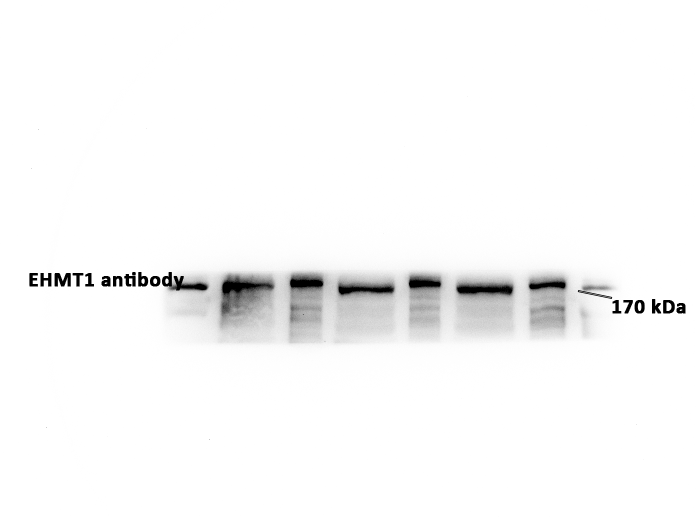

Supplement: Supplementary file 4 — Source Data [file 41467_2020_20242_MOESM4_ESM.zip › source data/Figure 6B/Figure 6B EHMT1.tif]

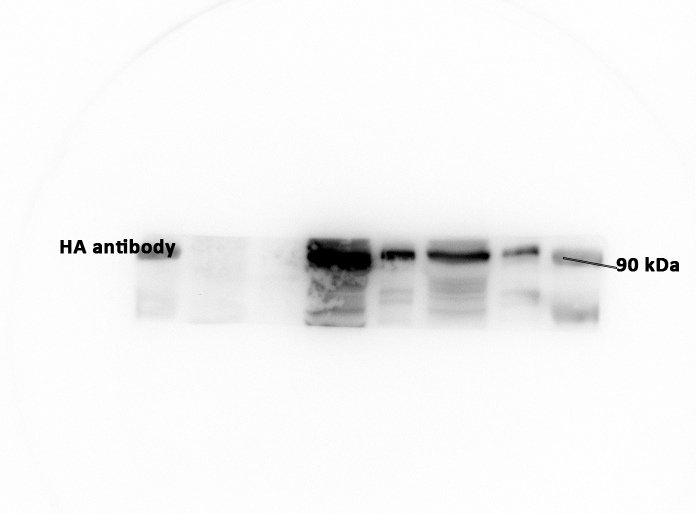

Supplement: Supplementary file 4 — Source Data [file 41467_2020_20242_MOESM4_ESM.zip › source data/Figure 6B/Figure 6B HA antibody.tif]

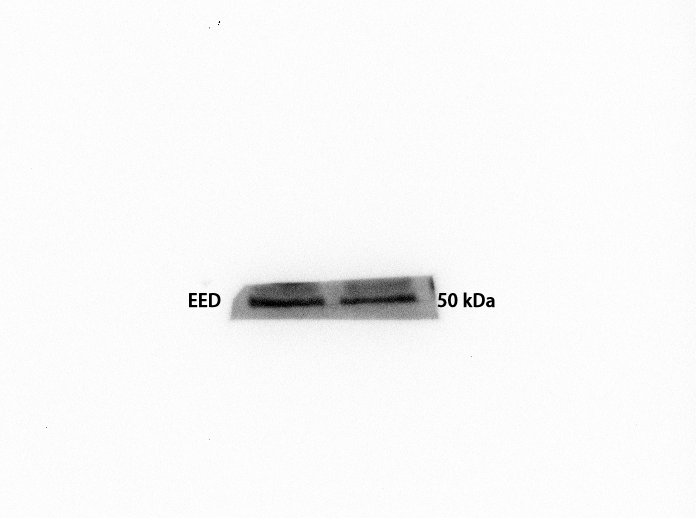

Supplement: Supplementary file 4 — Source Data [file 41467_2020_20242_MOESM4_ESM.zip › source data/Figure S5/Figure S5B EED.tif]

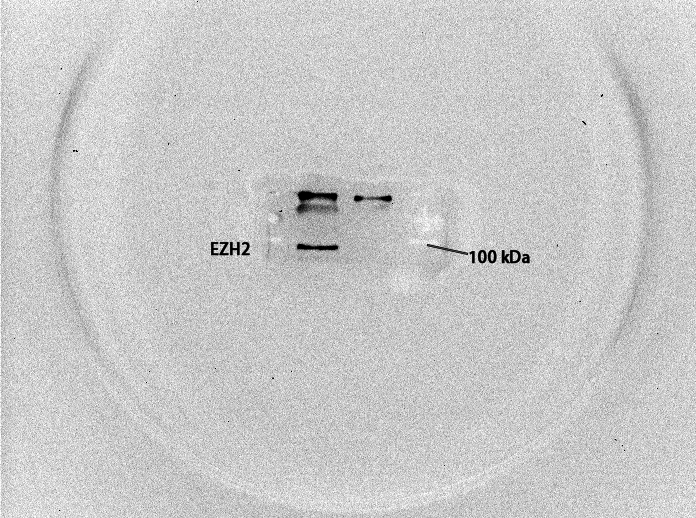

Supplement: Supplementary file 4 — Source Data [file 41467_2020_20242_MOESM4_ESM.zip › source data/Figure S5/Figure S5B EZH2.tif]

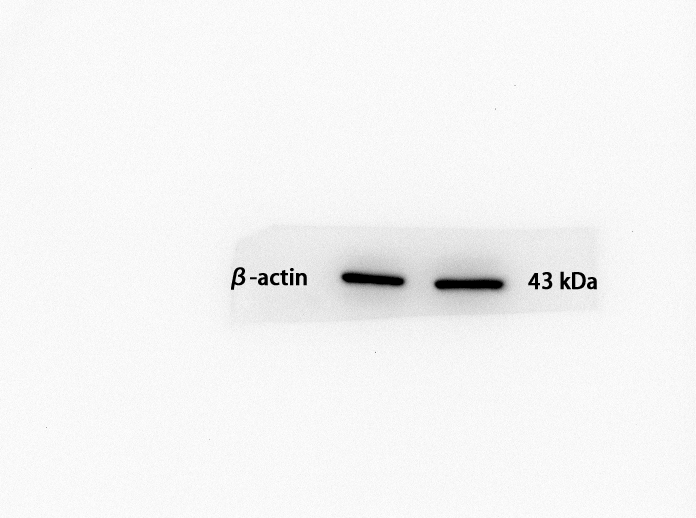

Supplement: Supplementary file 4 — Source Data [file 41467_2020_20242_MOESM4_ESM.zip › source data/Figure S5/Figure S5B β-actin.tif]

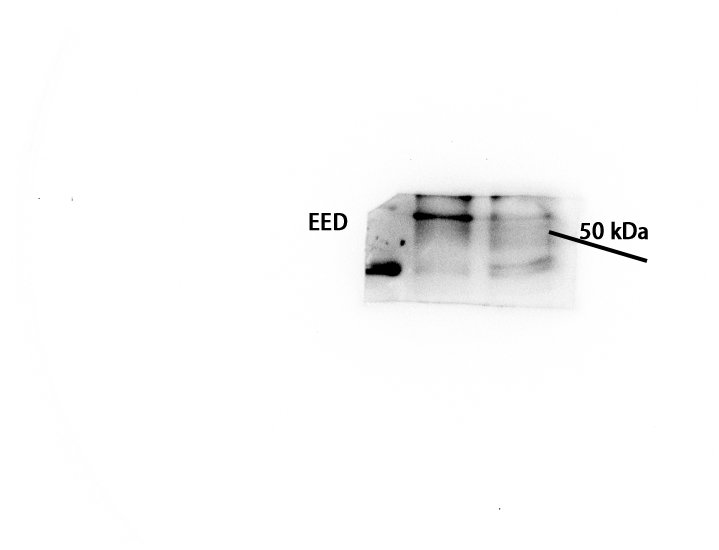

Supplement: Supplementary file 4 — Source Data [file 41467_2020_20242_MOESM4_ESM.zip › source data/Figure S5/Figure S5C EED.tif]

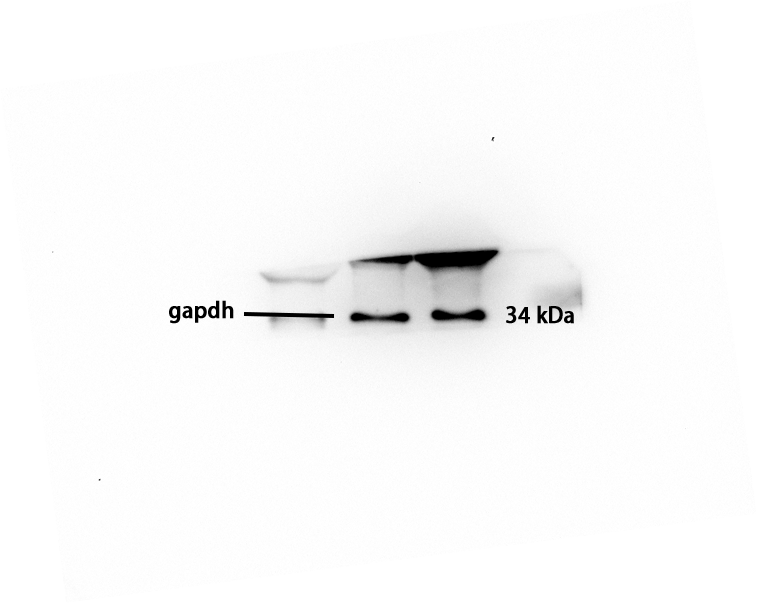

Supplement: Supplementary file 4 — Source Data [file 41467_2020_20242_MOESM4_ESM.zip › source data/Figure S5/Figure S5C gapdh.tif]

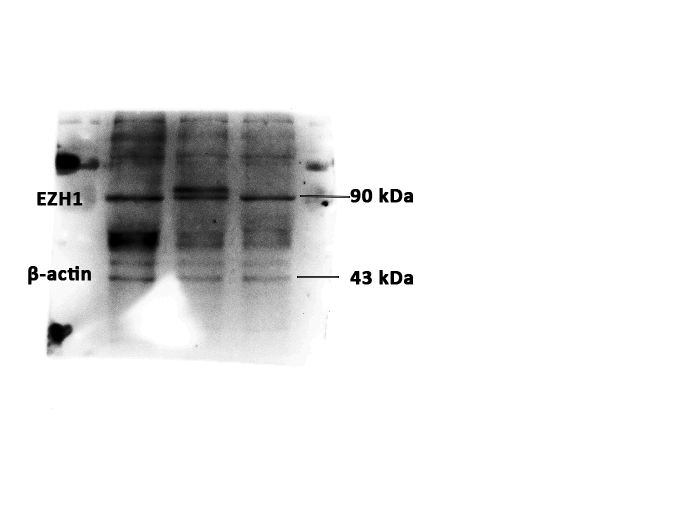

Supplement: Supplementary file 4 — Source Data [file 41467_2020_20242_MOESM4_ESM.zip › source data/Figure S5/Figure S5D .tif]

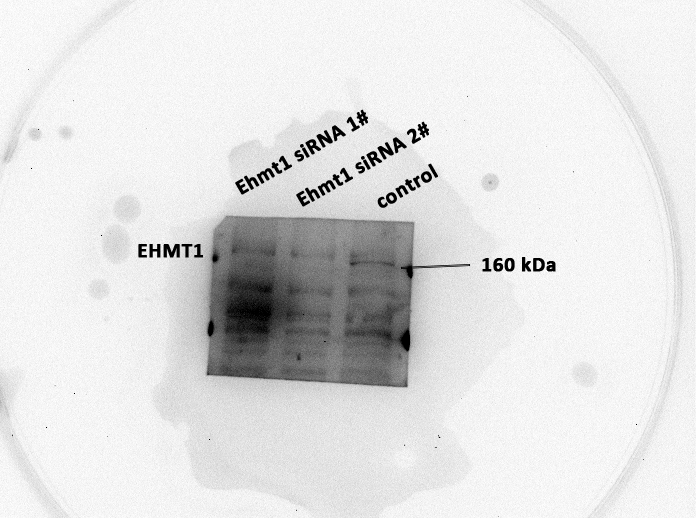

Supplement: Supplementary file 4 — Source Data [file 41467_2020_20242_MOESM4_ESM.zip › source data/Figure S6/Figure S6C EHMT1.tif]

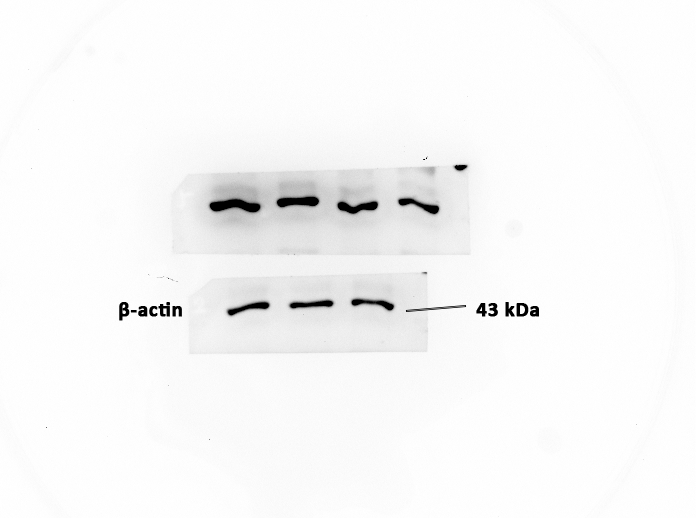

Supplement: Supplementary file 4 — Source Data [file 41467_2020_20242_MOESM4_ESM.zip › source data/Figure S6/Figure S6C β-actin.tif]

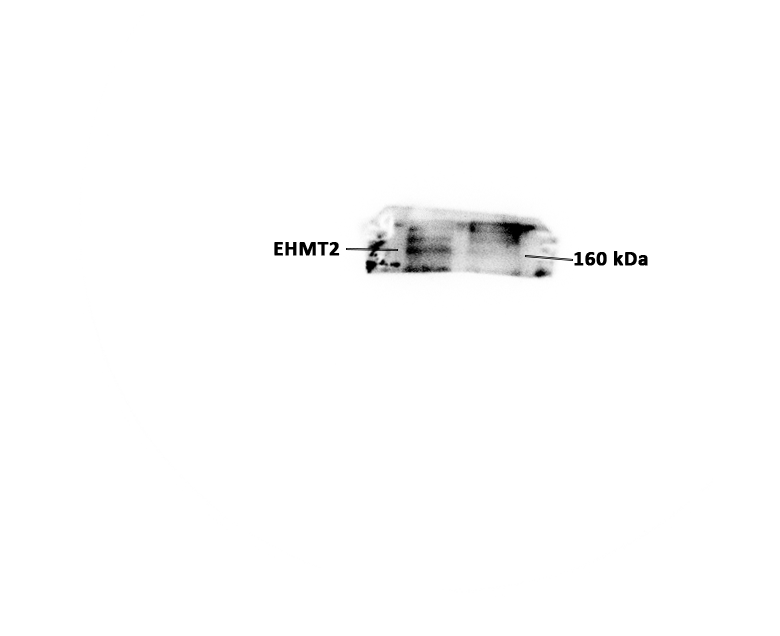

Supplement: Supplementary file 4 — Source Data [file 41467_2020_20242_MOESM4_ESM.zip › source data/Figure S6/Figure S6D EHMT2.tif]

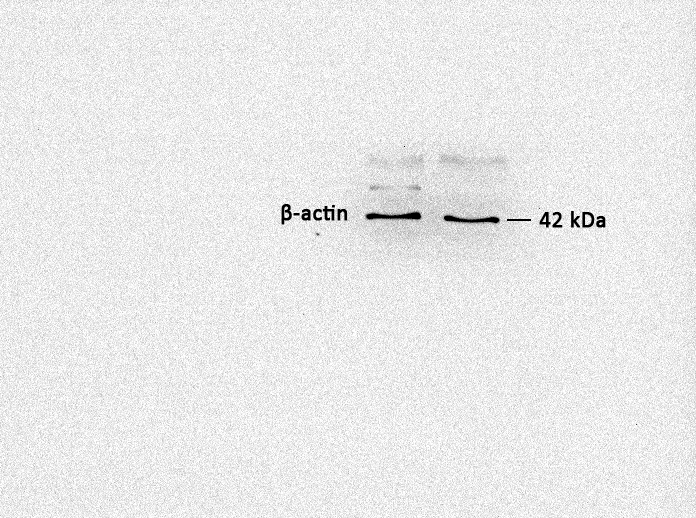

Supplement: Supplementary file 4 — Source Data [file 41467_2020_20242_MOESM4_ESM.zip › source data/Figure S6/Figure S6D β-actin.tif]

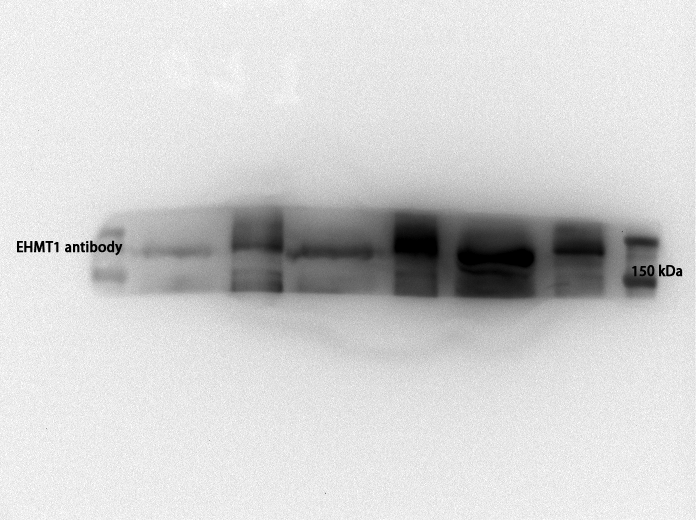

Supplement: Supplementary file 4 — Source Data [file 41467_2020_20242_MOESM4_ESM.zip › source data/Figure S7/Figure S7A EHMT1 antibody.tif]

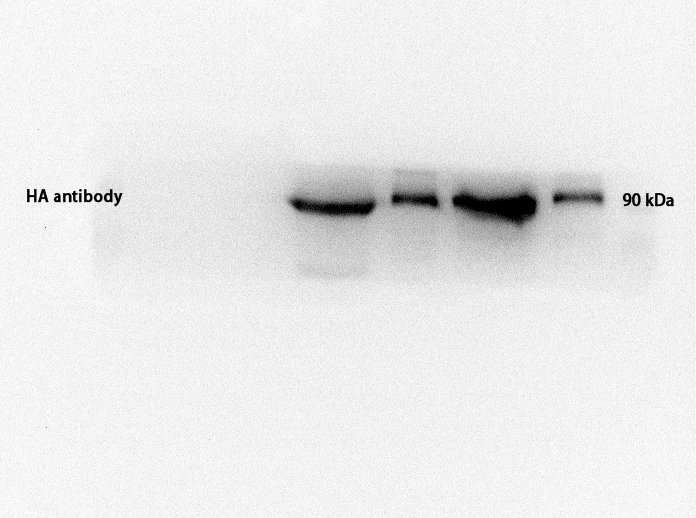

Supplement: Supplementary file 4 — Source Data [file 41467_2020_20242_MOESM4_ESM.zip › source data/Figure S7/Figure S7A HA antibody.tif]
