# Supplementary material for: PRC2 and EHMT1 regulate H3K27me2 and H3K27me3 establishment across the zygote genome
Source: Nat Commun. 2020 Dec 11;11:6354. doi: 10.1038/s41467-020-20242-9 (PMC7733509; doi:10.1038/s41467-020-20242-9)
Supplement: Supplementary file 2 — Peer Review File [file 41467_2020_20242_MOESM2_ESM.pdf]

Editorial Note: Parts of this peer review file have been redacted as indicated to remove third-party material where no permission to publish could be obtained.

Reviewers' comments:

Reviewer #1 (Remarks to the Author):

In the manuscript entitled, “Regulatory mechanisms of H3K27me2 and H3K27me3 establishment in mouse zygotes”, Meng and colleagues investigate the mechanism for catalyzing H3K27me2 and H3K27me3 in zygotes”. Their surprising finding that EZH2 does not confer H3K27me2, leads to the pursuit and identification of other proteins, involved in H3K27me2, including EZH1 and GLP, but not G9A. The authors employ many technologies to investigate the mechanism of H3K27me2, at a stage that is often prohibitive due to it being at the single-cell level. However, there are many issues that must be addressed.

Major Comments
1. The manuscript needs a complete rewrite. The introduction is too long (4.5 pages), in some cases repetitive, needs to focus on preimplantation development in mouse (including relevant data from Erhardt Development 2003; Inoue Nature 2017; Inoue Gene Dev 2018; Wang Epigenetics 2019) and then describe the outstanding question in the field that they will address. The results section does not provide enough rationale for the various experiments, making it difficult to understand the data. Furthermore, the discussion is mostly a repetition of the introduction without a specific focus on preimplantation development and restates the results. The discussion does not place the author's data in relationship to the literature nor the future experiments that are needed.

2. The authors have identified an EZH2-independent mechanism regulation H3K27me2 in zygotes. Yet, in contrast to their data, they still attribute a role for EZH2 via redundancy. I don’t think “redundant/redundancy” is the correct term to use (Page 8, line 192, 197, 218, and elsewhere). The word “redundant” means that another enzyme in addition to EZH2 catalyzes H3K27me2. Instead, the author's data show that EZH2 does not confer this activity in zygotes. Additionally, EZH1 cannot compensate for EZH2, if EZH2 is not responsible for the catalytic activity (lines 209 and 214). Fig 7 needs to be revised to reflect the author's data.

3. What was the rationale for assessing H3K27me2 prior to primordial follicle formation? Why not examine MII oocytes to determine whether zygotes potential inherit H3K27me2 from oocytes? The data from blastocysts in Fig 2D would be better suited here with the primordial follicle data with the rationale something like “This surprising result that EZH2 was not responsible for H3K27me2 in zygotes lead us to question whether H3K27me2 at other development stages was dependent on the catalytic activity of EZH2, such as prior to primordial follicle formation and in blastocyst.”

4. Page 8, line 203-204, the authors state that “there was no significant change in the level of overall H2K27me2 landscape in mouse zygotes”. However, in Fig 1D, the maternal genome H3K27me2 in Ezh2m- sample appears to be half the amount compared to the controls, and was more similar to levels of enrichment on the paternal chromosomal region. It would be helpful to have another image at a greater resolution of this region. In contrast, in Fig. 1F and Fig. S2E, the maternal genome in the Ezh2m- sample exhibited greater levels of methylation. The question is whether there are regions with lower and regions with higher methylation at specific genomic regions in the Ezh2m- sample? Furthermore, in Fig 1F, the authors need to perform allelic comparison/statistics for M-WT to M-KO, which appear to have different distribution and means, as well as for P-WT and P-KO. Finally, since Inoue et al (Nature 2017) reported changes in H3K27me3 methylation at H3K27me3-DNA methylation-independent imprinting regions, the authors should specifically report on H3K27me2 at these regions as well as the DNA methylation-dependent imprinting regions.

5. Page 9, lines 219-228, I disagree with the interpretation of EZH1 data. The authors need to explain

the difference in antibody and siRNA results. I would suggest that the antibody treatment removed all EZH1 protein, while siRNA would remove Ezh1-sequestered maternal RNA in zygotes but would not eliminate maternal-stored EZH1 protein. In the former treatment, loss of H3K27me2 in both pronuclei cannot be attributed to EZH2, but instead to another protein. In the siRNA treatment, maternal Ezh1 mRNA would be depleted but not stored maternal EZH1 protein. Here, loss of some EZH1, may lead to loss of H3K27me2 only in the paternal pronucleus. At the least, the authors need to provide evidence for knock-down of EZH1 protein in zygotes.

6. Also, there is also no evidence that "EZH1 coordinates with EZH2 to establish de novo H3K27me2". The authors go on to state that "We suggested that EZH1 antibody that binds whole PRC2-EZH1 complex hindered other subunits' (EED, SUZ12 et al) dynamic assembly and disassembly with PRC2-EZH2" (page 9, lines 219-221), and that "These results suggest that EZH1 coordinates with EZH2 to establish de novo H3K27 di-methylation on paternal pronuclei" (page 9, lines 227-228). However, the authors show that H3K27me2 in maternal and paternal pronuclei was unaffected by maternal EZH2 deletion, and in a previous study (Erhardt et al Development 2003), maternal EED protein was absent in EZH2m- female pronucleus, indicating that EED required EZH2 for localization to pronuclei. If EED is absent from pronuclei upon EZH2 m- deletion, how can EED then interact with EZH1 to carry out H2K27me2 in the PRC2 complex?

7. Page 10, line 249, the authors state that "there was no H3K27me2 and H3K27me3 before PN5 in mouse zygotes", do they mean in the paternal pronucleus?
8. The authors also need to provide evidence for knock-down of G9A and GLP protein in zygotes, since siRNAs may not be sufficient to deplete maternal stored proteins in the zygote.

9. Where is the data for UNC0638 for H3K27me2 (lines 268-271)? How do the authors explain that the G9A inhibitor depleted H3K27me2 (reported, data not shown), while G9A antibody and siRNA do not?

10. In Fig 6, the authors set out to determine whether there is interplay between GLP and EZH1 and EZH2. It is reasonable to determine whether H3K27me3 can be acquired by EZH2/1 in the absence of functional GLP. However, this aim was not accomplished. The authors show that H3K27me2 was catalyzed via the GLP-SET domain. However, the authors only show interactions between the proteins of interest via Co-IP. If the author really wanted to determine the interplay between GLP and EZH proteins, they should have examined H3K27me3, EZH1, EZH2 in GLP-SET deleted zygotes.

11. Figures: (1) Similar to Figure 4C, the authors should provide qualitative data (relative levels of intensity) for their fluorescent images, with statistical analysis. (2) For the fluorescent images, nuclei should be at a similar size/resolution as nuclei in Fig 5, to allow readers to better see data. (3) Since only one fluorophore is shown in figures, do the authors need to present the "merge" panel? (4) The image of blastocysts in Fig. 2D, needs to larger.

12. Since embryonic EZH2 protein is not present until the 4-cell stage and embryonic EED protein not likely present until the 8-cell stage, it would have been interesting to determine when there was a switch from EZH2/EED-independent H3K27me2 to EZH2/EED-dependent H3K27me2.

Minor comments
1. Correct terminology: Please introduce/use correct gene names- "G9A" is "EHMT2". "GLP" is EHMT1". In mouse, gene names are first letter capital (i.e. Ezh2) and protein names are all capitals (i.e. EZH2).

2. The authors use "H3K27 di-methylation" and "H3K27me2" interchangeably. Please use only one term (H3K27me2 is easier to read).

3. Page 5, line 130, I'm not sure what the authors mean by "agglutinated".

4. Page 5, line 130, I'm not sure what the authors mean by "newborn". De novo?

5. Page 6, line 35, state full names for the acronyms "ULI-NChIP", "EHMT2/G9A", EHMT1/GLP", "WIZ".

6. Page 7, line 184, after "(Fig.1A)", add "indicating that maternal EZH2 is present in both maternal and paternal pronuclei".

7. Page 8, line 201, it would be more accurate to state "H3K27me3 enrichment was observed at distinct features". Also, where is the H3K27me3 data from? Produced by the authors? From a database? Add "(Sup. Fig.2A-D)" for this data.

8. Page 9, line 223, "both of what?"

9. Page 9, lines 232-234, provide (supplementary) evidence that zygotes lack EED in maternal Eed deletion.

10. Page 10, lines 251-252, provide (supplementary) evidence that zygotes have EZH2 overexpression.

11. Page 11, lines 288-289, to the end of the title add "through EZH2".

12. Page 12, line 309, include maps of the constructs with domains indicated, or reference for constructs; were they made the authors?

13. Fig 3B, need WT zygotes controls.

14. Fig 4E. Place GLP data above G9a data, as was done in other panels.

Reviewer #2 (Remarks to the Author):

H3K27 methylation is an important repressive histone modification and dynamically regulated through the oocyte and embryo development. The deletion of maternal H3K27 methyltransferase results in growth defect in embryo, which indicates the H3K27 methylation plays an important role in embryo development. Recently, the maternal H3K27 methylation is reported to be inherited to the blastocyst and sever as an imprinting mark to regulate gene expression. However, how the histone modification enzyme cooperates to regulate H3K27 methylation is poorly studied. Authors try to investigate the role of these enzymes in establishing the H3K27 methylation in the early embryo.

Authors have examined the level of k27me3 and k27me2 in the oocyte and embryo from maternal conditional mice as well as by in vitro siRNA KD, antibody block and inhibitor treatment. The overall discovery is not surprising as the general mechanism is described before but would be still important for the researchers studying the epigenetic regulation in early embryos. However, there are several errors in the manuscript and make it hard to understand. The labels are not clear for the figures. Here are some major and minor comments:

Major comments:
1. As the author perturbed lots of epigenetic enzymes, including EZH2, EZH1, EED, and GLP, during

the oocyte and early embryo development. It would be great that the author can have a figure showing the expression pattern of these enzymes during the normal oocyte and early embryo development. Authors can either find them from published RNA-seq data or do staining for them. This should be a figure 1 before all the results.

2. For figure 1D, it is better there is staining for EZH2 for the same condition to confirm EZH2 is completely gone.
33. For the manuscript line 205-206, The statement "But the level of H3K27me2 on the paternal pronucleus was lower than that on the maternal (Fig.1F, Sup.Fig.2E)". Is it also supported by staining results? Does the KO show even lower than WT.

3. Figure 3A and manuscript line 252, the author mentioned it is figure k27me2 in the manuscript but labeled k27me3 in the figure. Also, as the paternal k27me methylation is present since PN5, authors should specify the stage in the manuscript and figure. Otherwise, it is hard to interpret the results.

4. It is clear the GLP is required for the K27me3 and K27me2 at the same time but there is still a possibility that the effect for K27me3 is not through K27me2 but other function from GLP, like the k9me3. So the author should discuss this possibility in the discussion and adjust the statement that "
33. H3K27me2 is an essential prerequisite for the following de novo H3K27me3 modification on the male pronucleus" into "H3K27me2 might be an essential prerequisite for the following de novo H3K27me3 modification on the male pronucleus".

Minor comments,

1. For manuscript line 135, that should be a different method other than ULI-NChIP.

2. For the manuscript line 178, in the title "Maternal EZH2 is indispensable for H3K27 trimethylation rather than demethylation in mouse zygotes". The demethylation should be dimethylation?

3. Please label the stage of zygote and oocyte for all the figures.

4. Figure S2A-D, please label the name of the sample

Reviewer #3 (Remarks to the Author):

Meng et al demonstrate that PRC2, through its catalytic subunit Ezh2, requires the histone methyltransferases GLP and Ezh1 (but not G9a) to catalyze efficient H3K27me3 in the male pronucleus. The authors demonstrate that the synergy between PRC2 and GLP requires the catalytic activity of GLP. The situation in the male pronucleus is different from that which has been described in mouse ESCs, where efficient H3K27me3 requires both GLP and G9a (Mozzetta et al Mol Cell 2014). The male pronucleus also appears to differ from ESCs, in that loss of Ezh2 or EED causes loss of H3K27me3, but does not appear to reduce levels of H3K27me2 (whereas in ESCs, loss of either EZH2 or EED causes dramatic loss of H3K27me 1/2/3). Certain controls and methodological details were lacking. If substantiated during revision, the submitted manuscript would provide another example where in different cell types, different proteins are involved in establishing H3K27me3 domains (as noted in the introduction, prior studies have demonstrated differential requirements for Ezh1 and Ezh2 in maintenance of H3K27me3 in different tissues). The general findings would be of interest to researchers studying Polycomb biology as well as biology associated with early embryogenesis. It remains an open question on what substrate GLP acts to promote H3K27me2.

1) Figure 1 – the authors suggest Ezh2 is not required for H3K27me2 in the male pronucleus of PN5 zygotes. More controls and quantitation are needed.

a) For all panels throughout the paper, it would be useful to have a sense of how many times a given IF was performed. Only 3 times? 10 times? How many embryos? There is a difference in quality between sparsely collected data and deeply sampled data.

b) Panels 1C and D: What is the H3K27me2 antibody being used? The product # and supplier are not given in the methods section. How can the authors be sure the antibody is specific for H3K27me2 and not cross reacting with other epitopes? As a reviewer, I cannot be sure.

c) Panel 1C: The authors do H3K27me2 IF Ezh2-maternal deletion zygotes and claim no reduction in H3K27me2 signal. The authors ought to quantify H3K27me2 fluorescence intensity in the Ezh2-maternal-KO embryos relative to WT.

d) Panel 1D: missing key controls. Please show Ezh2 and H3K27me3 IF in EZH2 KO oocytes that would confirm deletion.

e) Panel 1C and 1D: Again concerns about the specificity of the H3K27me2 antibody being used. Could the authors obtain EED KO ESCs and show that the H3K27me2 antibody being used is capable of detecting loss of H3K27me2 in a situation where it is known to be lost? The authors could also obtain H3K27me2 peptide and observe that the signal in EZH2 KO embryos can be competed off. These concerns are partially mitigated by later figures that show loss of H3K27me2 signal in EZH2 mutant blastocysts and GLP siRNA cells – still more controls would be welcomed.

f) Panel 1E/F: I understand the ULI-ChIP was a significant investment, but unfortunately, as it was performed, ULI-ChIP is not an assay that is quantitative enough to detect a reduction in H3K27me2 signal. It could distinguish between total absence and presence of signal. However, in order to claim no reduction in signal, the authors would have had to use spike-in control to normalize for differences between genotypes in terms of number of PCR amplification cycles that were used to generate the different libraries. A global decrease of 4-fold could easily be missed in the ChIP data. The mutant samples would have required more PCR amplification to obtain enough data for sequencing. The difference in PCR amplification would prevent a global decrease from being detected.

g) In regards to the ULI-ChIP assay, please cite the original study that developed the method. PMID: 25607992

Remaining figures – for the knockdowns, the over expression experiments, every result that is being depicted by IF – I would feel better as a reviewer if exact numbers of embryos measured were given, and statistics were used to determine differences. As the data were presented, the authors are asking reviewers to trust their statistical rigor, when it is a better practice to demonstrate the rigor by reporting numbers and replicates analyzed.

In the introduction, the authors should be aware of several recent studies published by the Reinberg lab which imply H3K27me activity of PRC2 is essential for its function. Beyond the work from the Reinberg lab, other recent and relevant PRC2 citations are missing.

In the conclusion, the authors need to update their citations. Every sentence that references prior

work should be concluded with citations to the work being referenced. Multiple works are implicitly referenced in the first paragraph of the discussion and none are cited. This should be fixed.

To Reviewer 1: We would like to thank you for your insightful comments and respond to the questions as follows.

Comment: In the manuscript entitled, "Regulatory mechanisms of H3K27me2 and H3K27me3 establishment in mouse zygotes", Meng and colleagues investigate the mechanism for catalyzing H3K27me2 and H3K27me3 in zygotes". Their surprising finding that EZH2 does not confer H3K27me2, leads to the pursuit and identification of other proteins, involved in H3K27me2, including EZH1 and GLP, but not G9A. The authors employ many technologies to investigate the mechanism of H3K27me2, at a stage that is often prohibitive due to it being at the single-cell level. However, there are many issues that must be addressed.

Major Comments

1. The manuscript needs a complete rewrite. The introduction is too long (4.5 pages), in some cases repetitive, needs to focus on preimplantation development in mouse (including relevant data from Erhardt Development 2003; Inoue Nature 2017; Inoue Gene Dev 2018; Wang Epigenetics 2019) and then describe the outstanding question in the field that they will address. The results section does not provide enough rationale for the various experiments, making it difficult to understand the data. Furthermore, the discussion is mostly a repetition of the introduction without a specific focus on preimplantation development and restates the results. The discussion does not place the author's data in relationship to the literature nor the future experiments that are needed.

Response: We thank the Reviewer for the careful review. As suggested, we have rewritten our manuscript. In brief, we shortened the introduction section, and highlighted some of the

recently published studies. At the same time, the results section was reorganized to communicate its logic as reasonable as possible. The Discussion section was focused on comparing our work with previous work, and analyzing the problems that need to be solved in the future.

2. The authors have identified an EZH2-independent mechanism regulation H3K27me2 in zygotes. Yet, in contrast to their data, they still attribute a role for EZH2 via redundancy. I don't think "redundant/redundancy" is the correct term to use (Page 8, line 192, 197, 218, and elsewhere). The word "redundant" means that another enzyme in addition to EZH2 catalyzes H3K27me2. Instead, the author's data show that EZH2 does not confer this activity in zygotes. Additionally, EZH1 cannot compensate for EZH2, if EZH2 is not responsible for the catalytic activity (lines 209 and 214). Fig 7 needs to be revised to reflect the author's data.

Response: We thank the Reviewer for this constructive advice. Many studies have proved that EZH is responsible for H3K27me2 and H3K27me3, and overexpression of EZH2 in mouse zygotes can indeed increase the modification level of H3K27me2. As mentioned by the Reviewer, it is not clear whether EZH2 or EHMT1 catalyzes H2K27me2 in mouse zygotes. To solve the Reviewer's concern, we have employed a dominant-negative *Ehmt1* mutant $Ehmt1^{C1201A}$ (containing the mutant SET-domain (C1201A) showing no enzymatic activity) to investigate whether Ehmt1 confers the catalytic activity of H3K27me2 in zygotes. Surprisingly, $Ehmt1^{C1201A}$ can still increase the level of zygote H3K27me2, which suggests that the methyltransferase activity of EHMT1 is dispensable to the establishment of H3K27me2 in zygotes. Therefore, we suggest that EZH2 or EZH1 confer the catalytic activity during the establishment of zygote H3K27me2. Of course, it cannot be ruled out that

the presence of other factors in the mouse zygote can catalyze H3K27me2. For logical rigor, we have modified "redundant / redundancy/compensate" to "partially safeguard", as suggested. Finally, following the Reviewer's suggestion we have revised the schematic figure in Fig. 7 to reflect our results.

3. What was the rationale for assessing H3K27me2 prior to primordial follicle formation? Why not examine MII oocytes to determine whether zygotes potential inherit H3K27me2 from oocytes? The data from blastocysts in Fig 2D would be better suited here with the primordial follicle data with the rationale something like "This surprising result that EZH2 was not responsible for H3K27me2 in zygotes lead us to question whether H3K27me2 at other development stages was dependent on the catalytic activity of EZH2, such as prior to primordial follicle formation and in blastocyst."

Response: We thank the Reviewer for the careful review. The reason why we detected H3K27me2 before the formation of the primordial follicles is that H3K27me2 still exists in *Ezh2*$^{fl/fl}$ *; Gdf9-Cre* oocytes, and Gdf9-Cre starts to be expressed in oocytes of primordial follicles and in later developmental stages since PD3. In addition, H3K27me2 was already present in oocytes at E14.5 (Sup. Fig.2A). To investigate whether the establishment of H3K27me2 at an earlier stage depends on the catalytic activity of EZH2, we used Vasa-Cre to delete *Ezh2* at an earlier stage. Furthermore, we thank the Reviewer for this excellent point of view and we agree with the Reviewer. Indeed, it is more logical to combine data from the blastocyst stage with primordial follicles. Following the Reviewer's suggestion, we have modified the relevant test in the revised text.

4. Page 8, line 203-204, the authors state that "there was no significant change in the level of

overall H2K27me2 landscape in mouse zygotes”. However, in Fig 1D, the maternal genome H3K27me2 in Ezh2m- sample appears to be half the amount compared to the controls, and was more similar to levels of enrichment on the paternal chromosomal region. It would be helpful to have another image at a greater resolution of this region. In contrast, in Fig. 1F and Fig. S2E, the maternal genome in the Ezh2m- sample exhibited greater levels of methylation. The question is whether there are regions with lower and regions with higher methylation at specific genomic regions in the Ezh2m- sample? Furthermore, in Fig 1F, the authors need to perform allelic comparison/statistics for M-WT to M-KO, which appear to have different distribution and means, as well as for P-WT and P-KO. Finally, since Inoue et al (Nature 2017) reported changes in H3K27me3 methylation at H3K27me3-DNA methylation-independent imprinting regions, the authors should specifically report on H3K27me2 at these regions as well as the DNA methylation-dependent imprinting regions.

Response: We thank the Reviewer for this constructive advice. The landscape of H3K27me2 is not changed dramatically after maternal EZH2 deletion; this is illustrated in Sup. Fig. 3C&F, which shows the distribution of H3K27me2 in different regions of the genome. After EZH2 knockout, H3K27me2 is still mainly distributed on the distal intergenic region without obvious pattern change. However, when comparing the distribution of H3K27me2 at each region, we find that in the maternal genome, the H2K37me2 signal at the promoter and the distal intergenic region was slightly decreased, while at UTR and exon regions, H3K27me2 was slightly increased. In the paternal genome, the H2K37me2 signal at the promoter (<=1kb), UTR, exon and distal intergenic region was slightly decreased, while at intron regions, H3K27me2 was slightly increased. This suggests that EZH2 knockout has different effects on different regions of the parental genome. When comparing the amount of the H3K27me2 in maternal and paternal genomes, they do differ quantitatively, which is shown

in Fig. 1L. We performed the allelic comparison according to the suggestion. As shown in Fig. 1L, after Ezh2 knockout the H3K27me2 signal in the maternal and paternal genome decreased significantly from the wild type. In accordance with the total H3K27me2 signal, our study of parts of the genome also showed the same trend. In two regions on chromosome 1&9, the RPKM of H3K27me2 was decreased in Mat and Pat genomes after EZH2 knockout (Fig.1K, Sup. Fig.3A). As shown in Sup. Fig. 3B, H3K27me2 is not significantly associated with DNA methylation-dependent imprinting regions in mouse zygotes, taking Inpp5f and Snrpn as examples.

5. Page 9, lines 219-228, I disagree with the interpretation of EZH1 data. The authors need to explain the difference in antibody and siRNA results. I would suggest that the antibody treatment removed all EZH1 protein, while siRNA would remove Ezh1-sequestered maternal RNA in zygotes but would not eliminate maternal-stored EZH1 protein. In the former treatment, loss of H3K27me2 in both pronuclei cannot be attributed to EZH2, but instead to another protein. In the siRNA treatment, maternal Ezh1 mRNA would be depleted but not stored maternal EZH1 protein. Here, loss of some EZH1, may lead to loss of H3K27me2 only in the paternal pronucleus. At the least, the authors need to provide evidence for knock-down of EZH1 protein in zygotes.

Response: We thank the Reviewer for the careful review. Actually, we know little about the difference between Ezh1 siRNA and EZH1 antibody. In siRNA treatment, maternal EZH1 protein has been depleted in mouse zygotes (Figure 2A). In regard to the difference in antibody and siRNA in the manuscript of the previous version, we speculated that EZH1 antibody might bind the whole PRC2-EZH1 complex and hinder other subunits' (EED, SUZ12 et al.) dynamic assembly and disassembly with PRC2-EZH2. To confirm this we

analyzed the protein level of EED in zygotes microinjected with EZH1 antibody. It turned out that the protein level of EED was obviously decreased in contrast to the positive control. Please see the results as follows.

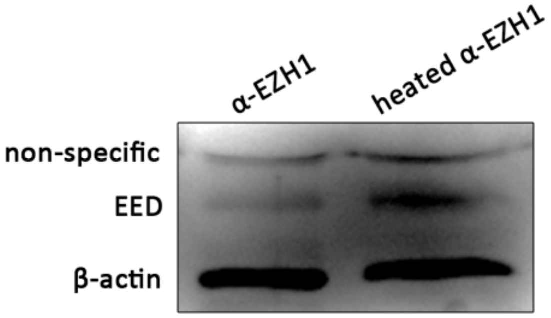

However, it should be noted that a small amount of EED can still be detected even after the antibody injection. Probably, other relevant factors may have crosstalk with EZH1 antibody. Following the Reviewer ʻs suggestion, we have removed the data of EZH1 antibody to ensure rigor.

6. Also, there is also no evidence that "EZH1 coordinates with EZH2 to establish de novo H3K27me2". The authors go on to state that "We suggested that EZH1 antibody that binds whole PRC2-EZH1 complex hindered other subunits' (EED, SUZ12 et al) dynamic assembly and disassembly with PRC2-EZH2" (page 9, lines 219-221), and that "These results suggest that EZH1 coordinates with EZH2 to establish de novo H3K27 di-methylation on paternal pronuclei" (page 9, lines 227-228). However, the authors show that H3K27me2 in maternal and paternal pronuclei was unaffected by maternal EZH2 deletion, and in a previous study (Erhardt et al Development 2003), maternal EED protein was absent in EZH2m- female pronucleus, indicating that EED required EZH2 for localization to pronuclei. If EED is absent from pronuclei upon EZH2 m- deletion, how can EED then interact with EZH1 to carry out H2K27me2 in the PRC2 complex?

Response: We thank the Reviewer for this constructive advice. It is indeed an interesting

question that the phenotypes of *Ezh2* and *Eed* maternal knockout are inconsistent. The former displays a severe growth retardation, while the latter shows significant reduced litter size and growth with a significant overgrowth. If EED required EZH2 for localization to pronuclei in *Ezh2*$^{m-/p-}$ embryos, the phenotypic difference between the two knockout mice cannot be determined. Here we would like to include some explanation regarding this issue. We tested the protein level of EED in oocytes of maternal knockout EZH2 and found that it did not significantly decrease (Sup. Fig. 5B). Considering that compared to maternal knockout EZH2, H3K27me2 was significantly decreased in the zygotes with maternal knockout EED (Fig. 2F&G). We speculated that there may be other factors in the mouse zygotes to functionally compensate for EZH2. Microinjection of *Ezh1* siRNA in oocytes with maternally knocked out EZH2 could indeed prevent the de novo H3K27me2 in the male pronucleus of zygotes (Fig. 2B&C). As for Erhardt et al reporting that EZH2 affected distribution of EED, we assumed that it may because they adopted parthenogenetic embryos lacking maternal EZH2. Indeed, there are reports that the depletion of *Ezh2* in highly proliferating fetal stem cells resulted in failure of hematopoiesis and cardiogenesis even in the presence of EZH1, indicating the complementation between EZH1 and EZH2 is cellular context-dependent. To fully uncover the underlying mechanisms controlling this important event, there is still a long way to go for complete clarification in the field.

7. Page 10, line 249, the authors state that “there was no H3K27me2 and H3K27me3 before PN5 in mouse zygotes”, do they mean in the paternal pronucleus?

Response: We thank the Reviewer for the careful review. We have modified our text in the revision and added “in the paternal pronucleus(Page8, line204).

8. The authors also need to provide evidence for knock-down of G9A and GLP protein in zygotes, since siRNAs may not be sufficient to deplete maternal stored proteins in the zygote.

Response: Following the Reviewer's suggestion, we have performed all the Western blots and updated the figures (revised Sup. Fig. 6C&D).

9. Where is the data for UNC0638 for H3K27me2 (lines 268-271)? How do the authors explain that the G9A inhibitor depleted H3K27me2 (reported, data not shown), while G9A antibody and siRNA do not?

Response: We thank the Reviewer for the careful review. We apologize for missing the data for UNC0638 for H3K27me2 icon. We have corrected this and added the data icon and updated the figures (revised Sup. Fig. 6A). UNC0638 is a potent, selective and cell-penetrant chemical probe for EHMT1 (GLP) and EHMT2 (G9a) by competing with the peptide substrate but not with the cofactor SAM. In other words, the inhibitory effect of inhibitors on de novo H3K27me2 is possible through EHMT1 and/or EHMT2. To further confirm whether EHMT1 or EHMT2 is responsible for this, we used EHMT1 and EHMT2 specific antibodies and siRNA for screening and verification, respectively. It was finally confirmed that only EHMT1 participated in this process.

10. In Fig 6, the authors set out to determine whether there is interplay between GLP and EZH1 and EZH2. It is reasonable to determine whether H3K27me3 can be acquired by EZH2/1 in the absence of functional GLP. However, this aim was not accomplished. The authors show that H3K27me2 was catalyzed via the GLP-SET domain. However, the authors only show interactions between the proteins of interest via Co-IP. If the author really wanted

to determine the interplay between GLP and EZH proteins, they should have examined H3K27me3, EZH1, EZH2 in GLP-SET deleted zygotes.

Response: We thank the Reviewer for this excellent point and we agree with the Reviewer. Through Co-IP experiments, we found that EHMT1$_{\triangle SET}$ can still interplay with EZH proteins (revised Sup. Fig. 7A). However, in the zygotes of EHMT1$_{\triangle SET}$, EZH2 could not be located to the male pronucleus normally (revised Sup. Fig. 7B). In addition, there was a near loss of de novo H3K27me2 in the paternal pronucleus after EHMT1$_{\triangle SET}$ overexpression (Fig. 6D&E). In addition, in order to further analyze whether the catalytic activity of EHMT1 is necessary for the de novo H3K27me2 in zygotes, we constructed a point mutation EHMT1^{C1201A}, which contains the mutant SET-domain (C1201A) showing no enzymatic activity, but it still can form a heterodimeric complex with EHMT2. Consistent with the full-length EHMT1 zygotes, the level of H3K27me2 in the zygotes of EHMT1^{C1201A} increased significantly, while H3K27me3 did not change significantly (revised Sup. Fig. 7F&G). Therefore, we suggest that the SET domain of EHMT1 rather than its catalytic activity is essential to de novo H3K27me2.

11. Figures: (1) Similar to Figure 4C, the authors should provide qualitative data (relative levels of intensity) for their fluorescent images, with statistical analysis. (2) For the fluorescent images, nuclei should be at a similar size/resolution as nuclei in Fig 5, to allow readers to better see data. (3) Since only one fluorophore is shown in figures, do the authors need to present the “merge” panel? (4) The image of blastocysts in Fig. 2D, needs to larger.

Response: We thank the Reviewer for the careful review. (1) We have performed all the quantification of all the fluorescent images with statistical analysis (Student’s t test). (2) We have modified the fluorescent images to make nuclei size similar in the revision (Fig.5A,

C&E). (3) We agree with the Reviewer and we have removed the "merge" panel in all the fluorescent images. (4) We have enlarged the image of blastocysts in the revision (Fig.1I).

12. Since embryonic EZH2 protein is not present until the 4-cell stage and embryonic EED protein not likely present until the 8-cell stage, it would have been interesting to determine when there was a switch from EZH2/EED-independent H3K27me2 to EZH2/EED-dependent H3K27me2.

Response: We thank the Reviewer for this excellent point. According to the existing RNA-seq data of the preimplantation embryo stages (Zhang et al, 2016, Nature), we found that the mRNA of *Ezh2* and *Eed* is not present until the 4-cell stage (revised Sup. Fig. 1A). The levels between mRNA and protein are often inconsistent for some genes. To solve the Reviewer 's concern, we have performed Western blot to analyze the expression profiles of EZH1, EZH2, and EED during the pre-implantation embryo stages, and found that EZH2 and EED were expressed at all stages, while EZH1 disappeared rapidly after the zygote stage (Fig. 1A). As mentioned by the Reviewer, this is indeed an interesting question that when there was a transition from EZH2/EED-independent H3K27me2 to EZH2/EED-dependent H3K27me2. We crossed Ezh2fl/fl;Gdf9-Cre female mice with Ezh2fl/fl;Vasa-Cre male mice to generate $Ezh2^{m-/p-}$ embryos and compared the H3K27me2 level between $Ezh2^{m+/p-}$ embryos and $Ezh2^{m-/p-}$ embryos during pre-implantation. The $Ezh2^{m-/p-}$ embryos began to show significant decrease of H3K27me2 from the morula stage (Fig. 1I&J, Sup. Fig. 2B). Based on our results, this transition might occur around the morula stage. Future investigations of H3K27 methylation during pre-implantation will likely elucidate the accurate time occurring for this switch and the mechanisms controlling this important physiological event through optimized technologies.

Minor comments

1. Correct terminology: Please introduce/use correct gene names- “G9A” is “EHMT2”. “GLP” is EHMT1”. In mouse, gene names are first letter capital (i.e. Ezh2) and protein names are all capitals (i.e. EZH2).

Response: We apologize for causing this misunderstanding. We agree with the reviewer’s point to unify the term “GLP” to “EHMT1”, and “G9A” to “EHMT2”. In addition, we unified all the gene names (first letter capital) and protein names (all capitals), as suggested.

2. The authors use “H3K27 di-methylation” and “H3K27me2” interchangeably. Please use only one term (H3K27me2 is easier to read).

Response: Following the Reviewer’s suggestion, we have unified “H3K27 di-methylation”to “H3K27me2”. In addition, “H3K27 tri-methylation” was unified to “H3K27me3” in revised manuscript.

3. Page 5, line 130, I’m not sure what the authors mean by “agglutinated”.

Response: We thank the Reviewer for the careful review. We want to express it as “decondensation”. In order to make the "Introduction section description more rational, we deleted this content in the revised manuscript.

4. Page 5, line 130, I’m not sure what the authors mean by “newborn”. De novo?

Response: We thank the Reviewer for the careful review. In order to make the "Introduction

section description more rational, we deleted this content in the revised manuscript. Besides, we unified the description of "de novo H3K27... " as "de novo" in revised manuscript.

5. Page 6, line 35, state full names for the acronyms “ULI-NChIP”, “EHMT2/G9A”, EHMT1/GLP”, “WIZ”.

Response: Following the Reviewer’s suggestion we wrote the acronym first, followed by the full name in the revision.

6. Page 7, line 184, after “(Fig.1A)”, add “indicating that maternal EZH2 is present in both maternal and paternal pronuclei”.

Response: We have added “which also indicates that maternal EZH2 is present in both maternal and paternal pronuclei” after “(Fig.1B)”in the revised manuscript (Page 5, line 128-129).

7. Page 8, line 201, it would be more accurate to state “H3K27me3 enrichment was observed at distinct features”. Also, where is the H3K27me3 data from? Produced by the authors? From a database? Add “(Sup. Fig.2A-D)” for this data.

Response: We thank the Reviewer for the careful review and agree We have changed the description in the revised manuscript. As for H3K27me3 data, we used data about H3K27me3 in mouse zygotes reported by Hui Zheng et al. (Molecular Cell,2016). In addition, we have added pie charts to show the proportion of H3K27me3 peaks distribution across the genome in the revised manuscript (Sup. Fig. 3G&H).

8. Page 9, line 223, “both of what?”

Response: We thank the reviewer for the careful review and we apologize for missing the description of EZH1 siRNA Western blot. We have added it in the revised manuscript. (Page 7 line 180-181)

9. Page 9, lines 232-234, provide (supplementary) evidence that zygotes lack EED in maternal Eed deletion.

Response: Following the reviewer's suggestion we have added Western blot image showing zygotes lacking EED in maternal *Eed* deletion (Sup. Fig. 5C).

10. Page 10, lines 251-252, provide (supplementary) evidence that zygotes have EZH2 overexpression.

Response: Following the reviewer's suggestion we have added western blot image and fluorescence images showing the zygotes having EZH2 overexpression (Sup. Fig. 5D&E).

11. Page 11, lines 288-289, to the end of the title add "through EZH2".

Response: Following the reviewer's suggestion we have added "through EZH2" in the revised manuscript (Page 10, line 248).

12. Page 12, line 309, include maps of the constructs with domains indicated, or reference for constructs; were they made the authors?

Response: Following the reviewer's suggestion we have added schematic representation of *myc-Ehmt1*, *myc-Ehmt1* $_{\triangle SET}$ and *myc-Ehmt1* ^{C1201A} with domains indicated in the revised manuscript (Fig. 6A), which are constructed by us.

13. Fig 3B, need WT zygotes controls.

Response: We apologize for causing this misunderstanding. Actually, Fig.3C (Figure3B in

the unrevised vision) shows the positioning of EZH2 in WT zygotes during PN1 to PN5. To be clear, we add “WT” in Fig.3C.

14. Fig 4E. Place GLP data above G9a data, as was done in other panels.

Response: We thank the reviewer for this constructive advice. Following the reviewer’s suggestion, we have placed EHMT1 data above EHMT2 data in all figures.

To Reviewer #2: We thank and appreciate your careful review of our manuscript and the helpful suggestions.

Reviewer #2 (Remarks to the Author):

H3K27 methylation is an important repressive histone modification and dynamically regulated through the oocyte and embryo development. The deletion of maternal H3K27 methyltransferase results in growth defect in embryo, which indicates the H3K27 methylation plays an important role in embryo development. Recently, the maternal H3K27 methylation is reported to be inherited to the blastocyst and sever as an imprinting mark to regulate gene expression. However, how the histone modification enzyme cooperates to regulate H3K27 methylation is poorly studied. Authors try to investigate the role of these enzymes in establishing the H3K27 methylation in the early embryo.

Authors have examined the level of k27me3 and k27me2 in the oocyte and embryo from maternal conditional mice as well as by in vitro siRNA KD, antibody block and inhibitor treatment. The overall discovery is not surprising as the general mechanism is described before but would be still important for the researchers studying the epigenetic regulation in

early embryos. However, there are several errors in the manuscript and make it hard to understand. The labels are not clear for the figures. Here are some major and minor comments:

Major comments:

1. As the author perturbed lots of epigenetic enzymes, including EZH2, EZH1, EED, and GLP, during the oocyte and early embryo development. It would be great that the author can have a figure showing the expression pattern of these enzymes during the normal oocyte and early embryo development. Authors can either find them from published RNA-seq data or do staining for them. This should be a figure 1 before all the results.

Response: We thank the Reviewer for this important point. As suggested, we analyzed the mRNA levels of genes such as EZH2 at various stages according to the existing RNA-seq data of the preimplantation embryo stages (Zhang et al. 2016, Nature). In addition, we also provided the protein level of EZH2, EZH1, and EED at various stages (Fig. 1A, Sup. Fig 1A&B). We previously reported that EHMT1 protein was detected in the nucleus of mouse germinal vesicle (GV) oocytes throughout differentiation to the blastocyst stage.

2. For figure 1D, it is better there is staining for EZH2 for the same condition to confirm EZH2 is completely gone.

Response: We totally understand the concern of the reviewer about the specificity of H3K27me2 antibody. Since both H3K27me2 and EZH2 antibodies are anti-rabbit, they cannot be incubated at the same time during the immunofluorescence procedure. To solve the reviewer's concern, we ordered the H3K27me2 peptide (Diagenode, C16000046-50) and injected it into the WT zygotes. Using the H3K27me2 antibody (Cell Signaling, 9728S) did not

detect a fluorescent signal, which indicates that the H3K27me2 antibody (Cell Signaling, 9728S) is specific. Combined with the data of *Eed* maternal knockout that results in the loss of H3K27me2, we believe that the H3K27me2 antibody (Cell Signaling, 9728S) is specific for the H3K27me2 site. At the same time, to further verify the reliability of our results, we used another H3K27me2 antibody (Mybiosource, MBS126234), and the results were consistent with the staining results of H3K27me2 (Cell Signaling, 9728S).

33. For the manuscript line 205-206, The statement "But the level of H3K27me2 on the paternal pronucleus was lower than that on the maternal (Fig.1F, Sup.Fig.2E)". Is it also supported by staining results? Does the KO show even lower than WT.

Response: We thank the Reviewer for the careful review. The conclusion that the level of H3K27me2 on the paternal pronucleus was lower than that on the maternal was drawn based on both statistical analysis of fluorescence intensity and chip data analysis (Fig. 1E, F&K). As for KO versus WT, the KO did not show even lower than WT according to the statistical analysis of fluorescence intensity. Considering the low resolution of immunofluorescence detection, we performed analysis of the chip data. The landscape of H3K27me2 has not changed dramatically after maternal *Ezh2* deletion; this result is illustrated in Sup.Fig.3C-F, which shows the distribution of H3K27me2 in different regions of the genome. After *Ezh2* knockout, H3K27me2 is still mainly distributed on the distal intergenic region without obvious pattern change. However, when comparing the distribution of H3K27me2 at each region, we find that in the maternal genome, the H2K37me2 signal at promoter and distal intergenic region was slightly decreased, while at UTR and exon regions, H3K27me2 was slightly increased. In the paternal genome, the H2K37me2 signal at promoter (<=1kb), UTR, exon and distal intergenic region was slightly decreased, while at intron regions, H3K27me2

was slightly increased. This suggests that *Ezh2* knockout has different effects on different regions of the parental genome.

When comparing the amount of the H3K27me2 in maternal and paternal genomes, they do differ quantitatively, which is shown in Fig.1L. We performed the allelic comparison according to the suggestion. As shown in Fig.1L, after *Ezh2* knockout the H3K27me2 signal in the maternal and paternal genome decreased significantly compared to the wild type.

3. Figure 3A and manuscript line 252, the author mentioned it is figure k27me2 in the manuscript but labeled k27me3 in the figure. Also, as the paternal k27me methylation is present since PN5, authors should specify the stage in the manuscript and figure. Otherwise, it is hard to interpret the results.

Response: We apologize for causing this misunderstanding. We have corrected this word as "H3K27me3" (Page 8, line206). Following the reviewer's suggestion, we have specified all the stages in the revised manuscript and figures.

4. It is clear the GLP is required for the K27me3 and K27me2 at the same time but there is still a possibility that the effect for K27me3 is not through K27me2 but other function from GLP, like the k9me3. So the author should discuss this possibility in the discussion and adjust the statement that "H3K27me2 is an essential prerequisite for the following de novo H3K27me3 modification on the male pronucleus" into "H3K27me2 might be an essential prerequisite for the following de novo H3K27me3 modification on the male pronucleus".

Response: We thank the reviewer for this important point and we agree. We have added related discussions in the Discussion part and adjusted the statement to "H3K27me2 might be an essential prerequisite for the following de novo H3K27me3 modification on the male

pronucleus”, as suggested by the reviewer. In addition, in order to further analyze whether the catalytic activity of EHMT1 is necessary for the de novo H3K27me2 in zygotes, we constructed a point mutation $EHMT1^{C1201A}$, which contains the mutant SET-domain (C1201A) showing no enzymatic activity, but it still can form a heterodimeric complex with EHMT1. Consistent with the full-length EHMT1 zygotes, the level of H3K27me2 in the zygotes overexpressing $EHMT1^{C1201A}$ increased significantly, while H3K27me3 did not change significantly. Therefore, we suggest that de novo H3K27me2 was catalyzed via the SET domain of EHMT1 rather than its catalytic activity.

Minor comments,

1. For manuscript line 135, that should be a different method other than ULI-NChIP.

Response: We thank the reviewer for the careful review. We have corrected this method as “STAR ChIP-seq” in the revised manuscript (Page 4, line89).

2. For the manuscript line 178, in the title “Maternal EZH2 is indispensable for H3K27 trimethylation rather than demethylation in mouse zygotes”. The demethylation should be dimethylation?

Response: We apologize for the mistake. We agree with the suggestion of the reviewer, and unified the term “H3K27 trimethylation” to “H3K27me3”, while “H3K27 dimethylation” to “H3K27me2”.

3. Please label the stage of zygote and oocyte for all the figures.

Response: We agree with the reviewer. We have labeled the stage of zygote and oocyte for all the figures.

4. Figure S2A-D, please label the name of the sample

Response: We agree with the reviewer. We have labeled the name of the sample

First of all, we would like to thank you very much for your critical review of our manuscript and respond to the questions as follows.

Reviewer #3 (Remarks to the Author):

Meng et al demonstrate that PRC2, through its catalytic subunit Ezh2, requires the histone methyltransferases GLP and Ezh1 (but not G9a) to catalyze efficient H3K27me3 in the male pronucleus. The authors demonstrate that the synergy between PRC2 and GLP requires the catalytic activity of GLP. The situation in the male pronucleus is different from that which has been described in mouse ESCs, where efficient H3K27me3 requires both GLP and G9a (Mozzetta et al Mol Cell 2014). The male pronucleus also appears to differ from ESCs, in that loss of Ezh2 or EED causes loss of H3K27me3, but does not appear to reduce levels of H3K27me2 (whereas in ESCs, loss of either EZH2 or EED causes dramatic loss of H3K27me 1/2/3). Certain controls and methodological details were lacking. If substantiated during revision, the submitted manuscript would provide another example where in different cell types, different proteins are involved in establishing H3K27me3 domains (as noted in the introduction, prior studies have demonstrated differential requirements for Ezh1 and Ezh2 in maintenance of H3K27me3 in different tissues). The general findings would be of interest to researchers studying Polycomb biology as well as biology associated with early

embryogenesis. It remains an open question on what substrate GLP acts to promote H3K27me2.

1) Figure 1 – the authors suggest Ezh2 is not required for H3K27me2 in the male pronucleus of PN5 zygotes. More controls and quantitation are needed.

Response: We thank the reviewer for the careful review. We have repeated all experiments at least 3 times and identified the number of tests with n. In addition, we have performed all the quantification of all the fluorescent images with statistical analysis (Student’s t test).

a) For all panels throughout the paper, it would be useful to have a sense of how many times a given IF was performed. Only 3 times? 10 times? How many embryos? There is a difference in quality between sparsely collected data and deeply sampled data.

Response: We thank the reviewer for the careful review. In order to ensure the reproducibility of the experimental results, each IF experiment was repeated at least 3 times, and at least 10 embryos were used for detection each time.

b) Panels 1C and D: What is the H3K27me2 antibody being used? The product # and supplier are not given in the methods section. How can the authors be sure the antibody is specific for H3K27me2 and not cross reacting with other epitopes? As a Reviewer, I cannot be sure.

Response: Thank you very much for your comments. We totally understand the concerns of the reviewers, and we have detailed the H3K27me2 antibody (Mybiosource, MBS126234) information used in the methods section. To solve the reviewer’s concern, we ordered the H3K27me2 peptide and injected it into the WT zygotes. Using the H3K27me2 antibody (Cell

Signaling, 9728S) did not detect a fluorescent signal, which indicates that the H3K27me2 antibody (Cell Signaling, 9728S) is specific. Combined with the data of Eed maternal knockout that results in the loss of H3K27me2, we believe that the H3K27me2 antibody (Cell Signaling, 9728S) is specific for the H3K27me2 site. At the same time, to further verify the reliability of our results, we used another H3K27me2 antibody (another), and the results were consistent with the staining results of H3K27me2 (Cell Signaling, 9728S).

c) Panel 1C: The authors do H3K27me2 IF Ezh2-maternal deletion zygotes and claim no reduction in H3K27me2 signal. The authors ought to quantify H3K27me2 fluorescence intensity in the Ezh2-maternal-KO embryos relative to WT.

Response: We thank the reviewer for the careful review. We have performed the quantification of the H3K27me2 fluorescent images with statistical analysis (Student's t test). The KO did not show even lower than WT according to the statistical analysis of fluorescence intensity. Considering the low resolution of immunofluorescence detection, we performed analysis of the chip data.

The landscape of H3K27me2 has not changed dramatically after maternal *Ezh2* deletion; this is illustrated in Sup.Fig.3C-F, which shows the distribution of H3K27me2 in different regions of the genome. After Ezh2 knockout, H3K27me2 is still mainly distributed on the distal intergenic region without obvious pattern change. However, when comparing the distribution of H3K27me2 at each region, we find that in the maternal genome, the H2K37me2 signal at the promoter, and distal intergenic region was slightly decreased, while at UTR and exon regions, H3K27me2 was slightly increased. In the paternal genome, the H2K37me2 signal at the promoter (<=1kb), UTR, exon and distal intergenic region was slightly decreased, while at intron regions, H3K27me2 was slightly increased. This suggests

that Ezh2 knockout has different effects on different regions of the parental genome. When comparing the amount of the H3K27me2 in maternal and paternal genomes, they do differ quantitatively, which is shown in Fig.1L. We performed the allelic comparison according to the reviewer's suggestion. As shown in Fig.1L, after maternal *Ezh2* knockout the H3K27me2 signal in the maternal and paternal genome decreased significantly compared to the wild type.

d) Panel 1D: missing key controls. Please show Ezh2 and H3K27me3 IF in EZH2 KO oocytes that would confirm deletion.

Response: We totally understand the concern of the reviewer about the specificity of H3K27me2 antibody. Since both H3K27me2 and EZH2 antibodies are anti-rabbit, they cannot be incubated at the same time during the immunofluorescence procedure. To solve the reviewer's concern, we ordered the H3K27me2 peptide and injected it into the WT zygotes. Using the H3K27me2 antibody (Cell Signaling, 9728S) we did not detect a fluorescent signal, which indicates that the H3K27me2 antibody (Cell Signaling, 9728S) is specific. Combined with the data of EED maternal knockout that results in the loss of H3K27me2, we believe that the H3K27me2 antibody (Cell Signaling, 9728S) is specific for the H3K27me2 site. At the same time, to further verify the reliability of our results, we used another H3K27me2 antibody (Mybiosource, MBS126234), and the results were consistent with the staining results of H3K27me2 (Cell Signaling, 9728S).

e) Panel 1C and 1D: Again concerns about the specificity of the H3K27me2 antibody being used. Could the authors obtain EED KO ESCs and show that the H3K27me2 antibody being used is capable of detecting loss of H3K27me2 in a situation where it is known to be lost?

The authors could also obtain H3K27me2 peptide and observe that the signal in EZH2 KO embryos can be competed off. These concerns are partially mitigated by later figures that show loss of H3K27me2 signal in EZH2 mutant blastocysts and GLP siRNA cells – still more controls would be welcomed.

Response: We thank the reviewer for the careful review. Wang et al reported the automethylation of PRC2 using the H3K27me2 antibody (Cell Signaling, 9728S), the same as we used。Although we did not obtain EED KO ESCs, we crossed conditional *Eed*$^{fl/fl}$ mice with *Gdf9-Cre* transgenic lines to generate embryos that were maternally deficient for EED in oocytes and zygotes, using the H3K27me2 antibody (Cell Signaling, 9728S) to find the loss of H3K27me2 fluorescence signal. In order to further solve the concern of the reviewer, we ordered the H3K27me2 peptide (Diagenode,C16000046-50), and the loss of the fluorescent signal was found after microinjection in EZH2 KO embryos, which further proved the specificity of the H3K27me2 antibody.

f) Panel 1E/F: I understand the ULI-ChIP was a significant investment, but unfortunately, as it was performed, ULI-ChIP is not an assay that is quantitative enough to detect a reduction in H3K27me2 signal. It could distinguish between total absence and presence of signal. However, in order to claim no reduction in signal, the authors would have had to use spike-in control to normalize for differences between genotypes in terms of number of PCR amplification cycles that were used to generate the different libraries. A global decrease of 4-fold could easily be missed in the ChIP data. The mutant samples would have required more PCR amplification to obtain enough data for sequencing. The difference in PCR amplification would prevent a global decrease from being detected.

Response: We thank reviewer very much for your understanding. Indeed, ULI-ChIP is not an

ideal analytical method sufficient to quantify the decrease in H3K27me2 signal. Unfortunately, there is currently no optimal way to achieve quantitative detection without signal loss. What we can do is to first detect the signal level of H3K27me2 by using immunofluorescence and draw a preliminary conclusion. Then use ULI-ChIP, a relatively high-resolution detection method, to further verify our conclusions.

g) In regards to the ULI-ChIP assay, please cite the original study that developed the method. PMID: 25607992

Response: We thank the reviewer for the careful review. We have added the original reference in the revised manuscript (Page4, line87).

Remaining figures – for the knockdowns, the over expression experiments, every result that is being depicted by IF – I would feel better as a Reviewer if exact numbers of embryos measured were given, and statistics were used to determine differences. As the data were presented, the authors are asking Reviewers to trust their statistical rigor, when it is a better practice to demonstrate the rigor by reporting numbers and replicates analyzed.

Response:

We thank the reviewer for the careful review. We have added the numbers of embryos and oocytes measured.

In the introduction, the authors should be aware of several recent studies published by the Reinberg lab which imply H3K27me activity of PRC2 is essential for its function. Beyond the work from the Reinberg lab, other recent and relevant PRC2 citations are missing.

Response: We thank the reviewer for the careful review. We have rewritten our manuscript and have added the recent study published by the Reinberg lab which implies that H3K27me

activity of PRC2 is essential for its function and we included recently published studies on H3K27 methylation.

In the conclusion, the authors need to update their citations. Every sentence that references prior work should be concluded with citations to the work being referenced. Multiple works are implicitly referenced in the first paragraph of the discussion and none are cited. This should be fixed.

Response: We thank the reviewer for the careful review. We have rewritten our manuscript and corrected reference errors according to the reviewer's suggestion.

REVIEWER COMMENTS

Reviewer #1 (Remarks to the Author):

In the revised manuscript, Meng and colleagues have improved the paper through the re-write, as well as increasing the rationale and clarity of the data.

Major Previous Comments

1. The manuscript needs a complete rewrite. The introduction is too long (4.5 pages), in some cases repetitive, needs to focus on preimplantation development in mouse (including relevant data from Erhardt Development 2003; Inoue Nature 2017; Inoue Gene Dev 2018; Wang Epigenetics 2019) and then describe the outstanding question in the field that they will address. The results section does not provide enough rationale for the various experiments, making it difficult to understand the data. Furthermore, the discussion is mostly a repetition of the introduction without a specific focus on preimplantation development and restates the results. The discussion does not place the author's data in relationship to the literature nor the future experiments that are needed.

Authors Response: The authors have addressed this comment.

Reviewer Response: I would like to ask that the authors consider one additional point that I couldn't discern from their data. Do the genomic H3K27me2 peaks in Fig 1k and Suppl Fig 3, directing correspond with the position and size of the H2K27me3 peaks? Or is there addition specificity for targeting EZH2 to confer H3K27me3 at smaller, larger or only a subset of H3K27me2 peaks? If it is the latter, it may raise additional future questions regarding the function of H3K27me2 and the mechanisms directing EZH2.

2. The authors have identified an EZH2-independent mechanism regulation H3K27me2 in zygotes. Yet, in contrast to their data, they still attribute a role for EZH2 via redundancy. I don't think "redundant/redundancy" is the correct term to use (Page 8, line 192, 197, 218, and elsewhere). The word "redundant" means that another enzyme in addition to EZH2 catalyzes H3K27me2. Instead, the author's data show that EZH2 does not confer this activity in zygotes. Additionally, EZH1 cannot compensate for EZH2, if EZH2 is not responsible for the catalytic activity (lines 209 and 214). Fig 7 needs to be revised to reflect the author's data.

Author Response: We thank the Reviewer for this constructive advice. Many studies have proved that EZH is responsible for H3K27me2 and H3K27me3, and overexpression of EZH2 in mouse zygotes can indeed increase the modification level of H3K27me2. As mentioned by the Reviewer, it is not clear whether EZH2 or EHMT1 catalyzes H2K27me2 in mouse zygotes. To solve the Reviewer's concern, we have employed a dominant-negative Ehmt1 mutant Ehmt1C1201A (containing the mutant SET-domain (C1201A) showing no enzymatic activity) to investigate whether Ehmt1 confers the catalytic activity of H3K27me2 in zygotes. Surprisingly, Ehmt1C1201A can still increase the level of zygote H3K27me2, which suggests that the methyltransferase activity of EHMT1 is dispensable to the establishment of H3K27me2 in zygotes. Therefore, we suggest that EZH2 or EZH1 confer the catalytic activity during the establishment of zygote H3K27me2. Of course, it cannot be ruled out that the presence of other factors in the mouse zygote can catalyze H3K27me2. For logical rigor, we have modified "redundant / redundancy/compensate" to "partially safeguard", as suggested. Finally, following the Reviewer's suggestion we have revised the schematic figure in Fig. 7 to reflect our results.

Authors Response: The terminology "partially safeguard" and "safeguard" are subjective. I would say it is a 2-step process with EZH1 (with EHMT1) conferring H3K27me2 and EZH2 conferring H3K27me3.

Furthermore, H3K27me2 may have a function beyond becoming H3K27me3 (as suggested in profiles in Fig S3.). Putting emphasis such as "safeguard for EZH2", indicates a less important role for EHZ1 and for H2K27me2 as an intermediate product. The title of this section may be "Maternal EZH1 is indispensable for H3K27me2 in the paternal pronucleus of mouse zygotes"

Minor Comments
1. Page 3, Line 59, It would be more accurate to state that "H3K27me3 is a hallmark of facultative heterochromatin".
2. Page 5, line 119-129 and Figure legend 1, the term "expression" is more accurately used to describe RNA. For protein, it is more accurate to describe levels or cellular position. Here, the authors show protein levels across stages. Levels should be quantitated (assuming 3 replicates were performed).
3. Figure 3B requires additional details. Where are the genes, promoters and differentially methylated region (for Snrpn)?
4. Page 11, line 278, Change "GLP" to "EHMT1".
5. Page 13, line 337, should it state "zygotes" rather than "oocytes"?

Reviewer #2 (Remarks to the Author):

I appreciate the experiments that the authors did and they did address some of my questions and concerns. However, I think the authors did not fully understand my comments on EZH2 staining in EZH2 KO and the concern of multiple functions of EHMT1.

I asked for EZH2 staining is to confirm the KO efficiency of EZH2, which is the foundation that their conclusion "loss of maternal EZH2 does not affect H3K27me2 signal" build on. The authors do not have to perform the staining together with k27me2 in the same cell.

I asked for a discussion of the possibility of other effects of GLP is because GLP is a known methyltransferase of k9me3. If authors would like to conclude that GLP function through k27me2 to affect k27me3 but not k9me3, an examination of k9me3 is needed. But authors can simply keep it as an open question if no experiment is performed. The set-domain mutation experiment does not answer my question but indicates that GLP might not be a major contributor to k27me2. I do not think "GLP as a novel contributor to the formation of H3K27me2" is a very solid conclusion, especially based on the new data of set-domain mutation. I suggest removing it from the abstract.

Reviewer #3 (Remarks to the Author):

I would like to thank the authors for their attention to my comments, which have all been addressed by their revisions.

To Reviewer #1: We would like to thank you for your insightful comments and respond to the questions as follows.

REVIEWER COMMENTS

Reviewer #1 (Remarks to the Author):

In the revised manuscript, Meng and colleagues have improved the paper through the re-write, as well as increasing the rationale and clarity of the data.

Major Previous Comments

1. *The manuscript needs a complete rewrite. The introduction is too long (4.5 pages), in some cases repetitive, needs to focus on preimplantation development in mouse (including relevant data from Erhardt Development 2003; Inoue Nature 2017; Inoue Gene Dev 2018; Wang Epigenetics 2019) and then describe the outstanding question in the field that they will address. The results section does not provide enough rationale for the various experiments, making it difficult to understand the data. Furthermore, the discussion is mostly a repetition of the introduction without a specific focus on preimplantation development and restates the results. The discussion does not place the author's data in relationship to the literature nor the future experiments that are needed.*

Authors Response: The authors have addressed this comment.

Reviewer Response: I would like to ask that the authors consider one additional point that I couldn't discern from their data. Do the genomic H3K27me2 peaks in Fig 1k and Suppl Fig 3, directing correspond with the position and size of the H2K27me3 peaks? Or is there addition specificity for targeting EZH2 to confer H3K27me3 at smaller, larger or only a subset of H3K27me2 peaks? If it is the latter, it may raise additional future questions regarding the function of H3K27me2 and the mechanisms directing EZH2.

Response: We appreciate the reviewer's insightful comments and explain as follows: we have screened the peaks between H3K27me2 and H3K27me3, and found that there were genomic H3K27me2 peaks directing correspond with the position and size of the H3K27me3 peaks. But there were H3K27me2 peaks not directing correspond with the H3K27me3. We must admit that ULI-NChIP-seq technology has certain limitations (including resolution and suitable antibodies), and cannot cover the entire genome with H3K27me2 and H3K27me3. With the future improvement of micro-sequencing technology and selection of more suitable antibodies, further studies will further verify our conclusions. We fully understand Reviewer's concern on the function of H3K27me2. The current manuscript is already enough lengthy and focuses on the establishment and regulatory mechanism of H3K27me2 and H3K27me3 in mouse zygotes. Instead, we are continuing another study focusing on the functions of H3K27me2.

2. *The authors have identified an EZH2-independent mechanism regulation H3K27me2 in zygotes. Yet, in contrast to their data, they still attribute a role for EZH2 via redundancy. I don't think "redundant/redundancy" is the correct term to use (Page 8, line 192, 197, 218, and elsewhere). The word "redundant" means that another enzyme in addition to EZH2 catalyzes H3K27me2. Instead, the author's data show that EZH2 does not confer this activity in zygotes. Additionally, EZH1 cannot compensate for EZH2, if EZH2 is not responsible for the catalytic activity (lines 209 and 214). Fig 7 needs to be revised to reflect the author's data.*

Author Response: We thank the Reviewer for this constructive advice. Many studies have proved that EZH is responsible for H3K27me2 and H3K27me3, and overexpression of EZH2 in mouse zygotes can indeed increase the modification level of H3K27me2. As mentioned by the Reviewer, it is not clear whether EZH2 or EHMT1

catalyzes H2K27me2 in mouse zygotes. To solve the Reviewer's concern, we have employed a dominant-negative Ehmt1 mutant Ehmt1C1201A (containing the mutant SET-domain (C1201A) showing no enzymatic activity) to investigate whether Ehmt1 confers the catalytic activity of H3K27me2 in zygotes. Surprisingly, Ehmt1C1201A can still increase the level of zygote H3K27me2, which suggests that the methyltransferase activity of EHMT1 is dispensable to the establishment of H3K27me2 in zygotes. Therefore, we suggest that EZH2 or EZH1 confer the catalytic activity during the establishment of zygote H3K27me2. Of course, it cannot be ruled out that the presence of other factors in the mouse zygote can catalyze H3K27me2. For logical rigor, we have modified "redundant / redundancy/compensate" to "partially safeguard", as suggested. Finally, following the Reviewer's suggestion we have revised the schematic figure in Fig. 7 to reflect our results.

Authors Response: The terminology "partially safeguard" and "safeguard" are subjective. I would say it is a 2-step process with EZH1 (with EHMT1) conferring H3K27me2 and EZH2 conferring H3K27me3. Furthermore, H3K27me2 may have a function beyond becoming H3K27me3 (as suggested in profiles in Fig S3.). Putting emphasis such as "safeguard for EZH2", indicates a less important role for EHZ1 and for H2K27me2 as an intermediate product. The title of this section may be "Maternal EZH1 is indispensable for H3K27me2 in the paternal pronucleus of mouse zygotes"

Response: We appreciate the reviewer's valuable suggestions for improving the rationality of our manuscript. **What needs to be explained is that for the Reviewer's suggestion** "I would say it is a 2-step process with EZH1 (with EHMT1) conferring H3K27me2 and EZH2 conferring H3K27me3." is not rigorous. In fact, there was normal de novo H3K27me2 in paternal pronuclei of $EZH2^{m+/p+}$ zygotes after *Ezh1* siRNA microinjection (Fig. 2B&C). However, there was complete absence of de novo H3K27me2 in paternal pronuclei of zygotes lacking maternal EZH2 after *Ezh1* siRNA microinjection (Fig. 2B&C).
We appreciate the Reviewer's suggestion: H3K27me2 may have a function beyond becoming H3K27me3 (as suggested in profiles in Fig S3.). Indeed, not all H3K27me2 will become H3K27me3. To ensure rigor and respect to the reviewer's opinion, we suggest "Maternal EZH1 is indispensable for H3K27me2 in the paternal pronucleus of $Ezh2^{m-/p+}$ mouse zygotes" is more reasonable.

Minor Comments
1. Page 3, Line 59, It would be more accurate to state that "H3K27me3 is a hallmark of facultative heterochromatin".

Response: Following the Reviewer's suggestion, we have modified the statement in the revised text.

2. Page 5, line 119-129 and Figure legend 1, the term "expression" is more accurately used to describe RNA. For protein, it is more accurate to describe levels or cellular position. Here, the authors show protein levels across stages. Levels should be quantitated (assuming 3 replicates were performed).

Response: Following the Reviewer's suggestion, we have modified the description of related results and quantitated the levels of related proteins.

3. Figure 3B requires additional details. Where are the genes, promoters and differentially methylated region (for Snrpn)?

Response: Following the reviewer's suggestion we have added additional details for Inpp5f and Snrpn (Sup.Fig. 3B).

4. Page 11, line 278, Change "GLP" to "EHMT1".

Response: We thank the reviewer for the careful review and apologies for missing this change. We have replaced "GLP" with "EHMT1".

5. Page 13, line 337, should it state "zygotes" rather than "oocytes"?
Response: We thank the reviewer for the careful review and we have replaced "oocytes" with "zygotes".

To Reviewer #2: We would like to thank you for your positive comments and respond to the questions as follows.

Reviewer #2 (Remarks to the Author):

I appreciate the experiments that the authors did and they did address some of my questions and concerns. However, I think the authors did not fully understand my comments on EZH2 staining in EZH2 KO and the concern of multiple functions of EHMT1.

I asked for EZH2 staining is to confirm the KO efficiency of EZH2, which is the foundation that their conclusion "loss of maternal EZH2 does not affect H3K27me2 signal" build on. The authors do not have to perform the staining together with k27me2 in the same cell.

I asked for a discussion of the possibility of other effects of GLP is because GLP is a known methyltransferase of k9me3. If authors would like to conclude that GLP function through k27me2 to affect k27me3 but not k9me3, an examination of k9me3 is needed. But authors can simply keep it as an open question if no experiment is performed. The set-domain mutation experiment does not answer my question but indicates that GLP might not be a major contributor to k27me2. I do not think "GLP as a novel contributor to the formation of H3K27me2" is a very solid conclusion, especially based on the new data of set-domain mutation. I suggest removing it from the abstract.

Response: We thank the Reviewer for this constructive advice. In fact, EHMT1 (GLP) is the H3K9me2 methyltransferase in vivo. As Tachibana et al. reported that the *Ehmt1*$^{-/-}$ ES cells exhibited a drastic reduction in mono- and di-methylated H3-K9 but no alteration in H3- K9 trimethylation. In fact, we did consider the possibility of EHMT1 function through H3K9me2 to affect H3K27me3. But we did not continue to explore this possibility for the following reasons: （1）H3K9me2 (as well as H3K9me3) has an asymmetric modification in mouse zygotes, which only exists on the female pronucleus in mouse zygotes[1]；

[Redacted]

（2）We previously demonstrated that the H3K9me2 level in mouse zygotes treated with BIX01294 (a selective inhibitor of G9a and GLP) showed no significant difference compared with the control zygotes[2].

[Redacted]

Of course, Kubicek et al reported that EHMT1 is capable of catalyzing formation H3K9me3 in an ***in vitro*** methyltransferase assay[3].

[Redacted]

Lu et al. recently reported that GLP catalyzes H4K16me1 in response to DNA damage in an ATM-dependent manner[4], which suggested GLP has a possibile role in regulating H3K27me3 through other functions. We thank the Reviewer for insightful suggestions and appreciate the logical rigor to study, we are pleased to add GLP might affect k27me3 through other function as an open question for further investigation in Discussion section in revised manuscript. Meanwhile, given that there was a virtually complete disappearance of de novo H3K27me2 and H3K27me3 in the paternal pronucleus after EHMT1$_{\Delta SET}$ overexpression, we suggest that the SET domain of EHMT1 is indispensable for de novo H3K27me2 in mouse zygotes. Based on the above statement, we changed the statement" GLP as a novel contributor to the formation of H3K27me2" to "EHMT1 is involved in the formation of H3K27me2"

To Reviewer #3: We would like to thank you for your positive comments on our work in revised manuscript.
Reviewer #3 (Remarks to the Author):

I would like to thank the authors for their attention to my comments, which have all been addressed by their revisions.

Reference

1. Burton, A. & Torres-Padilla, M.E. Epigenetic reprogramming and development: a unique heterochromatin organization in the preimplantation mouse embryo. *Briefings in functional genomics* 9, 444-454 (2010).

2. Ma, X.S. *et al.* The Dynamics and Regulatory Mechanism of Pronuclear H3k9me2 Asymmetry in Mouse

Zygotes. *Sci Rep* **5**, 17924 (2015).

3. Kubicek, S. *et al.* Reversal of H3K9me2 by a Small-Molecule Inhibitor for the G9a Histone Methyltransferase. *Molecular cell* **25**, 473-481 (2007).

4. Lu, X. *et al.* GLP-catalyzed H4K16me1 promotes 53BP1 recruitment to permit DNA damage repair and cell survival. *Nucleic Acids Research* **47**, 10977-10993 (2019).
